# Three-Component Access to Functionalized Spiropyrrolidine Heterocyclic Scaffolds and Their Cholinesterase Inhibitory Activity

**DOI:** 10.3390/molecules25081963

**Published:** 2020-04-23

**Authors:** Sarra Boudriga, Saoussen Haddad, Vikneswaran Murugaiyah, Moheddine Askri, Michael Knorr, Carsten Strohmann, Christopher Golz

**Affiliations:** 1 Department of Chemistry, Laboratory of Heterocyclic Chemistry Natural Product and Reactivity/CHPNR, Faculty of Science of Monastir, Monastir 5000, Tunisia; haddad_saoussen@live.fr; 2 Discipline of Pharmacology, School of Pharmaceutical Sciences, Universiti Sains Malaysia, USM 11800, Penang, Malaysia; vicky@usm.my; 3 Institut UTINAM-UMR CNRS 6213, Université Bourgogne Franche-Comté, 16 Route de Gray, 25030 Besançon, France; 4 Technische Universität Dortmund, Anorganische Chemie Otto-Hahn-Straße 6, 44221 Dortmund, Germany; carsten.strohmann@tu-dortmund.de (C.S.); christopher.golz@tu-dortmund.de (C.G.)

**Keywords:** [3+2]-cycloaddition reaction, azomethine ylides, succinimide, dispiropyrrolidine derivatives

## Abstract

A novel *one-pot* [3+2]-cycloaddition reaction of *(E)-*3-arylidene-1-phenyl-succinimides, cyclic 1,2-diketones (isatin, 5-chloro-isatin and acenaphtenequinone), and diverse *α-*aminoacids such as 2-phenylglycine or sarcosine is reported. The reaction provides succinimide-substituted dispiropyrrolidine derivatives with high regio- and diastereoselectivities under mild reaction conditions. The stereochemistry of these *N-*heterocycles has been confirmed by four X-ray diffraction studies. Several synthetized compounds show higher inhibition on acetylcholinesterase (AChE) than butyrylcholinesterase (BChE). Of the 17 synthesized compounds tested, five exhibit good AChE inhibition with IC_50_ of 11.42 to 22.21 µM. A molecular docking study has also been undertaken for compound **4n** possessing the most potent AChE inhibitory activity, disclosing its binding to the peripheral anionic site of AChE enzymes.

## 1. Introduction

**Figure 1:**
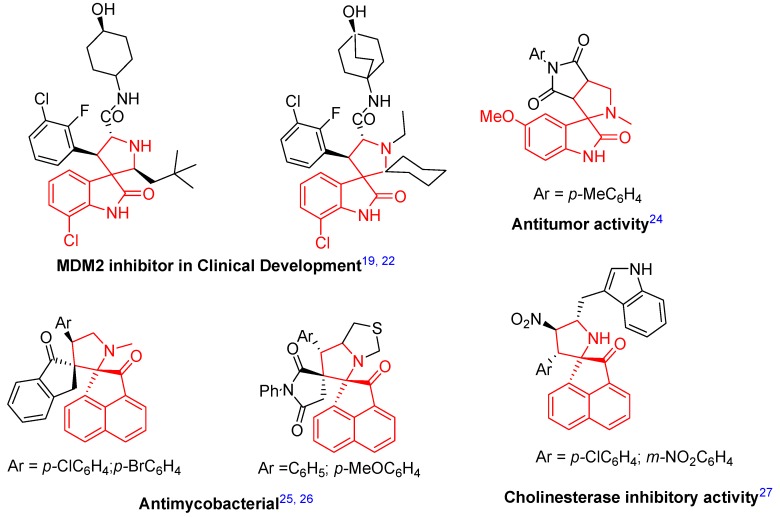
Clinical and biologically important spiropyrrolidines bearing acenaphthylene-1,2-dione or oxindole moieties.

Spiroheterocyclic scaffolds have evoked immense research interest in the area of synthetic organic chemistry and medicinal chemistry [1,2] since they incorporate the ubiquitous substructures present in a broad range of bioactive natural isolates and synthetic compounds [3]. They possess a wide spectrum of useful properties, such as anti-cancer [4,5,6,7], acetylcholinesterase (AChE) inhibition [8,9], anti-proliferative [10,11], antimicrobial [12,13], photochromism [14,15,16], and hole-transporting abilities [17]. One major advantage that spiroheterocycles offer as core structures is their structural rigidity and inherent structural complexity and ability to project functionality in all three dimensions [2,18], which provides an enhanced affinity to biotargets [19,20,21]. Spiropyrrolidines bearing acenaphthylene-1,2-dione or oxindole moieties are particularly relevant spiroheterocycles owing key structural feature of a plethora of bioactive synthetic and natural compounds which often show diverse biological, therapeutic, and physical properties [7,12,13,14,15,16,17,18,19,20,21,22,23,24,25,26,27,28,29,30,31,32,33,34] (Figure 1).

Among the typical synthetic strategies leading to these spiroheterocyclic scaffolds, the multicomponent 1,3-dipolar cycloaddition of electron-deficient exocyclic alkenes with azomethine ylides, generated in situ from *α-*aminoacids and 1,2-diketones, is the most widely reported one [35,36,37,38,39,40,41]. Its process simplicity combined with mild reaction conditions and atomic economy addresses several aspects required for combinatorial chemistry.

**Figure 2:**
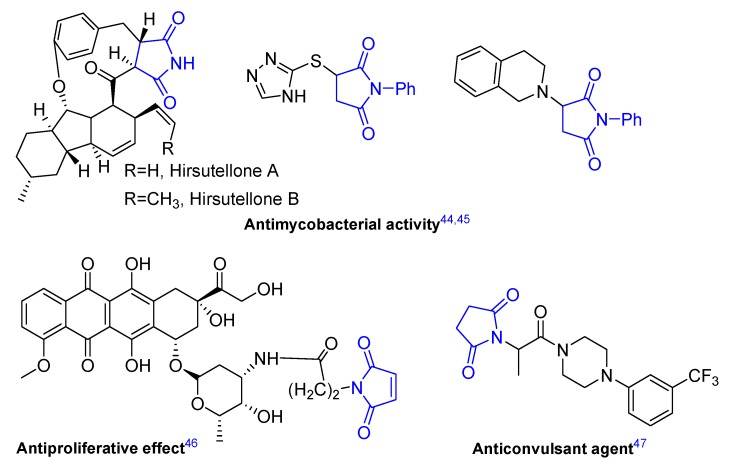
Examples of bioactive molecules containing succinimide unit.

On the other hand, the succinimide moiety is a core constituent of numerous alkaloids including Hirsutellone A and B 7, drug molecules and various synthetic compounds possessing diverse bioactivities [42,43]. Some succinimide derivatives, for instance, are used as antimycobacterial for treatment of Mycobacteria infections, [44,45] to suppress cancer cell proliferation [46] and act as anticonvulsant agents [47,48] (Figure 2).

**Figure 3:**
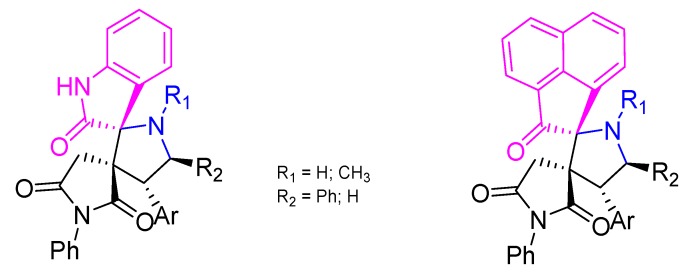
Design of novel succinimide-substituted dispiropyrrolidine derivatives.

Therefore, spiro-compounds that merge spiropyrrolidine and succinimide frameworks (Figure 3) can show intriguing possibilities for biological or other pharmacological properties. Nevertheless, no rational strategy has been developed so far to construct such a motif. 

Herein, and in continuation of our interest in azomethine ylide [3+2]-cycloaddition reaction and in the synthesis of novel spiroheterocycles [26,49,50,51,52,53], we report the synthesis of a novel series of dispiro[pyrrolidine-1-succinimide] derivatives by a *one-pot three*-component [3+2]-cycloaddition reaction of *(E)-*3-arylidene-1-phenyl-succinimides **1**, *α-*aminoacids **2** and the cyclic 1,2-diketones isatin **3a**–**b** or acenaphtenequinone **3c**. Furthermore, some selected heterocyclic compounds were screened in vitro to evaluate their cholinesterase inhibitory propensity. 

## 2. Results and Discussion

### 2.1. Chemistry

**Scheme 1:**
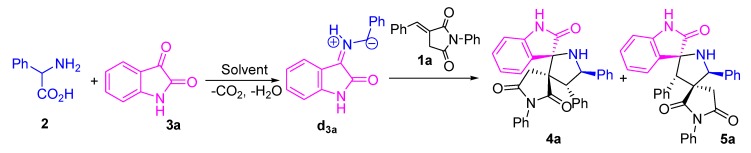
1,3-Dipolar cycloaddition reaction for the synthesis of dispiropyrrolidine **4a**.

The *three-*component 1,3-dipolar cycloaddition reaction of *(E)-*3-benzylidene-1-phenyl-succinimide 1a to azomethine ylide generated in situ from 2-phenylglycine **2** and the cyclic 1,2-diketone isatin **3a** was chosen as a testing system to optimize the synthetic conditions (Scheme 1). The effects of solvents, reaction time, and temperature were examined, and the results are summarized in Table 1.

Unfortunately, dispiropyrrolidine **4a** was obtained only in low yield when carrying out the reaction in water, toluene, or acetonitrile at room temperature (Table 1, entries 1–3). However, in refluxing acetonitrile, the [3+2]-cycloaddition proceeds smoothly affording the targeted dispiropyrrolidine with a significantly improved yield as a mixture of two regioisomers **4a** and **5a** in a 78:22 ratio (Table 1, entry 4). Next, the pilot reaction was examined in various protic solvents, such as EtOH, *i*PrOH, and MeOH (Table 1, entries 5–7). As shown in Table 1, methanol emerges as the solvent of choice, affording the targeted dispiropyrrolidines as a mixture of two regioisomers **4a** and **5a**, as single diastereoisomer*,* in good yield (77%) along with fairly high regiostereoselectivity (Table 1, entry 7). Then, we probed the reaction with MeOH/H_2_O mixtures both in a 1:1 and 2:1 ratio to further explore a more efficient and ecofriendly synthetic strategy (Table 1, entries 8–9). The most optimized reaction conditions for synthesizing dispiropyrrolidine **4a** were achieved by using imide **1a** (0.13 g, 0.5 mmol), 2-phenylglycine **2** (0.09 g, 1.2 eq.), and isatin **3a** (0.073 g, 1 eq.) in MeOH/H_2_O (2:1 *v/v*, 10 mL) under reflux temperature for 6 h. 

**Scheme 2:**
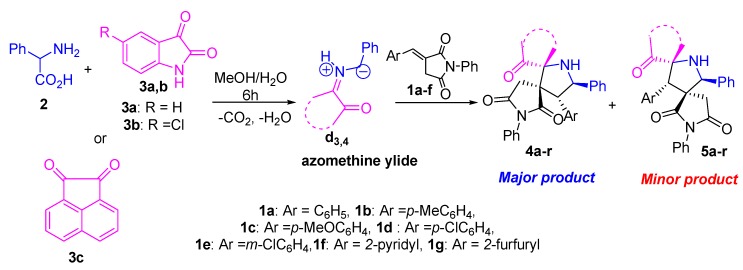
Reaction of *(E)-*3-arylidene-1-phenylpyrrolidine-2,5-dione **1a**–**g** with 2-phenylglycine **2** and cyclic 1,2-diketones **3.**

With the optimized synthetic protocol established (Table 1, entry 9), we extended the scope of this reaction to a wide range of *α-*arylidene-succinimides **1** bearing different substituents at the *p-*position of the aryl group or with heterocyclic group, as well as with various 1,2-diketones **3** (Scheme 2, Table 2). 

As summarized in Table 2, enones **1** bearing an electron-neutral (H), electron-rich (e.g., 4-Me or 4-OMe) group or electron-withdrawing substituent (4-Cl or 3-Cl) at the phenyl ring reacted well yielding the dispiropyrrolidine derivatives **4a**–**4e** in good to excellent yields (Table 2, entries 1–5). The reaction was even feasible with substrate **1** featuring pyridyl and furfuryl moieties, affording spiropyrrolidine **4f** and **4g** in excellent yields (Table 2, entries 6 and 7).

Pleasingly, also 5-chloroisatin **3b** underwent the reaction smoothly providing the final products **4h**–**l** in good yield along with high regioselectivities (Table 2, entries 8–12).

The generality of this protocol was further demonstrated by its compatibility with the cyclic 1,2-diketone acenaphthenequinone **3c** (Table 2, entries 13–18). The reaction delivers the desired products **4m**–**r** in excellent yields with a high level of regio- and diastereoselectivity.

**Scheme 3:**
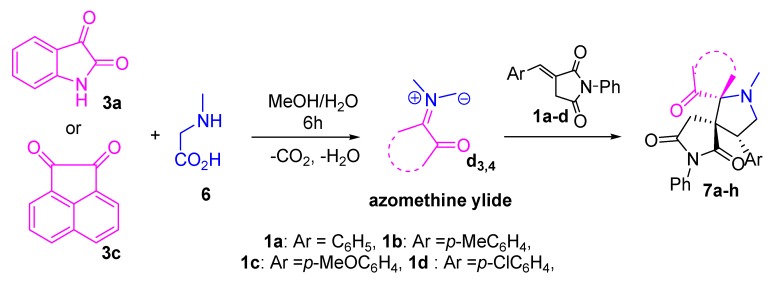
Reaction of *(E)-*3-arylidene-1-phenylpyrrolidine-2,5-diones **1a-f** with sarcosine **6** and cyclic 1,2-diketones **3**.

In order to further demonstrate the synthetic utility of the multicomponent 1,3-dipolar cycloaddition procedure, we further examined the regio- and diastereoselectivity of the reaction of the dipolarophiles **1**, the cyclic 1,2-diketones and sarcosine **6** as alternative *α-*aminoacid instead of 2-phenylglycine (Scheme 3).

The outcome indicated that this α-aminoacid is well compatible with this process, providing the cycloadducts **7a**–**h** in excellent yields with high regio- and diastereoselectivity, irrespective of the electronic property and steric demand of the substituent on the aryl ring of the succinimides (Table 3, entries 1–8).

### 2.2. Spectroscopic and Crystallographic Characterization of the Isomeric Cycloadducts

**Figure 4:**
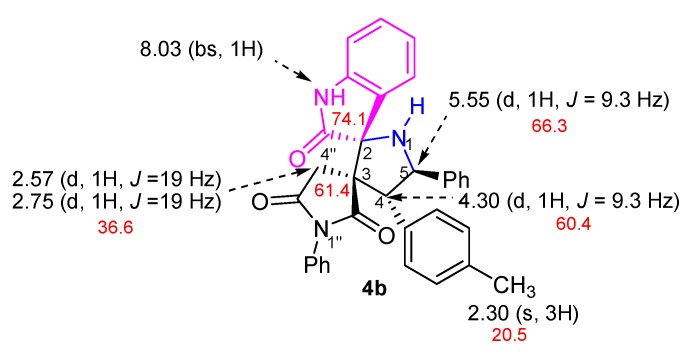
Selected ^1^H and ^13^C-NMR (red) chemical shifts of **4b**.

**Figure 5:**
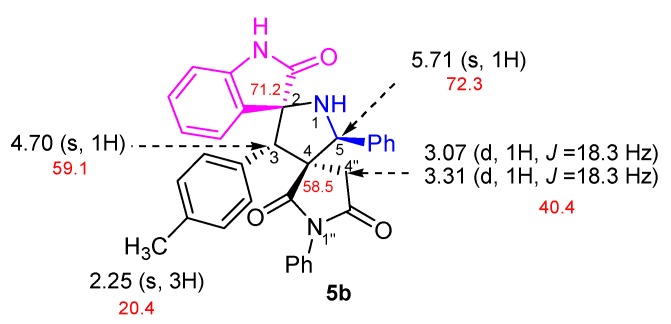
Selected ^1^H and ^13^C-NMR (red) chemical shifts of **5b**.

**Figure 6:**
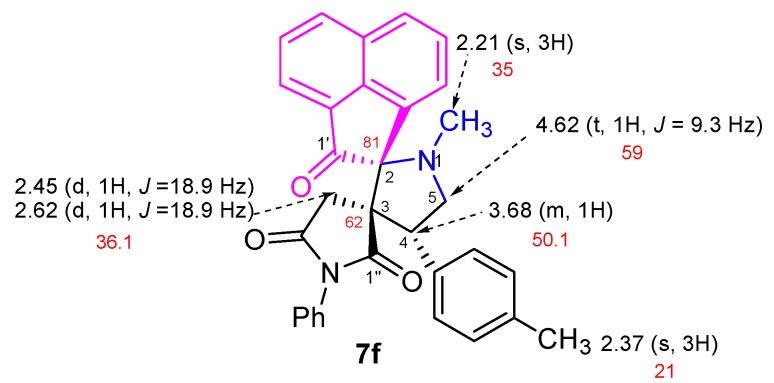
Selected ^1^H and ^13^C-NMR (red) chemical shifts of **7f**.

The structure and the relative configuration of the isomeric spiropyrrolidines, which have been isolated as colorless solids, were deduced both from NMR spectroscopy and from X-ray structure determinations performed on cycloadducts **4b**, **4m**, **5m,** and **7f**. Relevant ^1^H and ^13^C chemical shifts of dispiropyrrolidines **4b**, **5b,** and **7f** are shown in Figure 4, Figure 5 and Figure 6, respectively.

The ^1^H-NMR spectrum of **4b** exhibits two mutually coupled doublets centered at δ 2.57 and 2.75 ppm corresponding to the diastereotopic 4″-CH_2_ groups, as well as two doublets at δ 5.55 and 4.30 ppm assigned to the pyrrolidine H-5 and H-4 protons, respectively. Their coupling constants of approximately 9.3 Hz indicate that these protons are in *trans*-relationship. The occurrence and the multiplicity of these signals clearly prove the regiochemistry of the cycloaddition reaction. In contrast, in the ^1^H-NMR spectrum of regioisomer **5b** (Figure 5) the pyrrolidinyl protons H-5 and H-3 appear as two singlets resonating at δ 5.71 and 4.7 ppm, respectively. The ^13^C-NMR spectrum of the cycloadduct **4b** displays two peaks at δ 66.3 and 60.3 ppm corresponding to C-5 and C-4, respectively. The two spirocarbons C-2 and C-3 resonate at δ 74.1 and 58.5 ppm, respectively. In addition, the presence of three carbonyl groups is confirmed by the occurrence of three low-field peaks at δ 173.4, 178.8 and 179 ppm. 

The ^1^H-NMR spectrum of compounds **7f** shows two characteristic singlets at δ 2.21 and 2.37 ppm corresponding to the -NCH_3_ and -CH_3_ protons, respectively. The presence of these signals proves the incorporation of both dipole and dipolarophile **1b**. The triplet at δ 3.68 ppm and a multiplet at δ 4.62 ppm are assigned to the H-4 proton and the two geminal –CH_2_ protons of the spiropyrrolidine ring, respectively. Furthermore, the ^13^C-NMR spectrum of **7f** displays two signals at δ 62.0 and 81.0 ppm attributed to two spiranic carbons C-3 and C-2, respectively. The resonances at 173.4, 178.6, 207.1 ppm are characteristic of the presence of three carbonyl groups belonging to the succinimide and acenaphthenequinone moieties.

**Figure 7:**
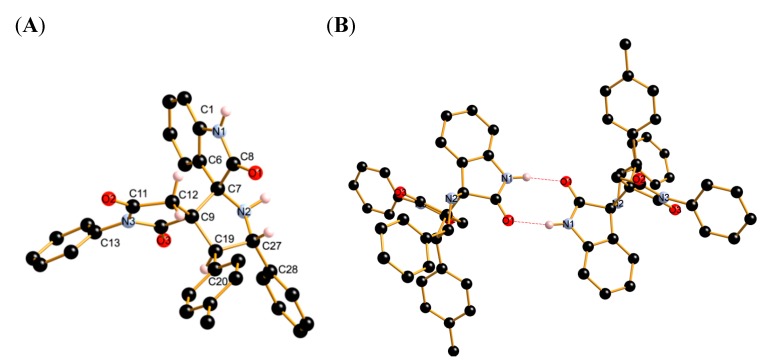
(**B**) Ball and Sticks presentation of the molecular structure of **4b** in the crystal. For clarity, only stereochemically significant hydrogen atoms are shown. Only one of the two independent molecules in the unit cell is presented. Selected bond lengths (Å) and angles (°): C1–N1 1.4144(17), N1–C8 1.3437(19), C8–O1 1.2329(17), C8–C7 1.5556(18), C6–C7 1.5126(19), C7–N2 1.4488(18), N2–C27 1.4669(19), C11–O2 1.2060(17), C11–N3 1.36965(18), C10–N3 1.3959(17); C7–C9–C19103.62(10), C9–C19–C27105.21(11), C9–C19–C27105.21(11), C19–C27–N2102.89(11), C27–N2–C7108.10(11), N2–C7–C8114.02(11), N2–C7–C8114.02(11), C7–C8–N1108.41(11), C8–N1–C1111.33(11), N1–C1–C6109.24(11), C6–C7–C9115.09(11), C9–C10–N3109.24(11), C10–N3–C11112.56(11). (**A**) Pairwise association of two molecules in the packing through intermolecular N-H····O hydrogen bonding. *d*(N-H····O 2.00(2) Å.

**Figure 8:**
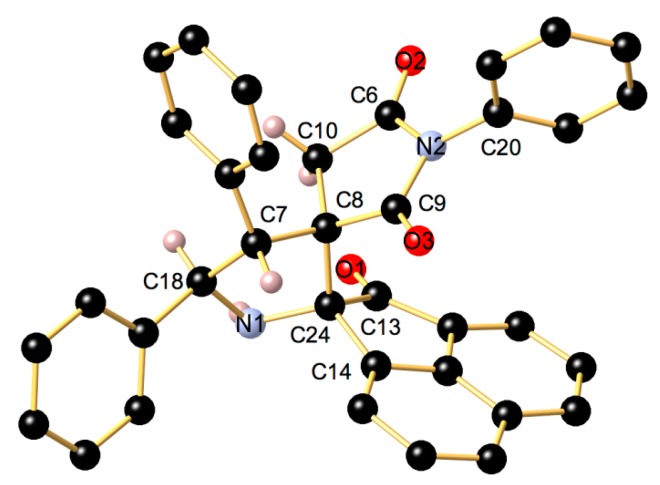
Ball and Sticks presentation of the molecular structure of **4m** in the crystal. For clarity, only stereochemically significant hydrogen atoms are shown. Selected bond lengths (Å) and angles (°):C6–N2 1.395(2), N2–C9 1.397(2), C6–O2 1.2093(18), C9–O3 1.2074(18), C13–O1 1.52152(18),C24–N1 1.461(2), N1–C18 1.4787(18); C8–C10–C6105.67(12), C10–C6–N2107.92(13), C6–N2–C9113.11(13), N2–C9–C8108.11(13), C9–C8–C24115.07(12), N1–C24–C8103.31(12), C24–N1–C18110.43(12), C7–C18–N1105.11(12), C18–C7–C8101.90(12), C7–C8–C10114.28(12).

**Figure 9:**
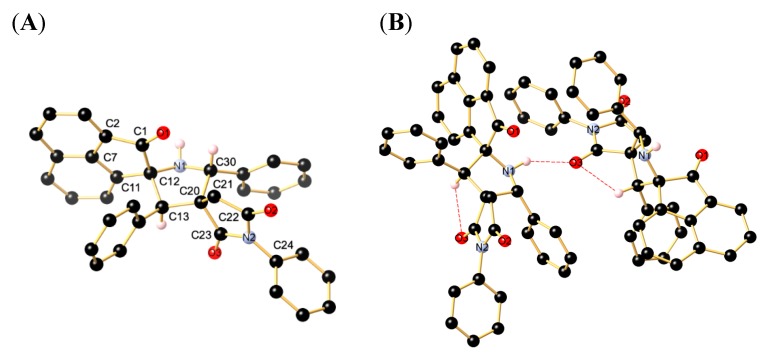
(**B**) Ball and Sticks presentation of the molecular structure of **5m** in the crystal. For clarity, only stereochemically significant hydrogen atoms are shown. Selected bond lengths (Å) and angles (°): C12–N1 1.462(3), N1–C30 1.469(3), C1–O1 1.217(3), C22–O2 1.212(3), C23–O3 1.204(3), C22–N2 1.394(3), N2–C23 1.4787(18); C20–C13–C12 104.46(17), C12–N1–C30 106.91(17), N1–C30–C20 112.96(18), C20–C21–C22 104.86(18), N2–C22–C21 107.52(18), C22–N2–C23 112.24(19), N2–C23–C20 107.98(19), N2–C23–O3 124.4(6). (**A**) Association of two molecules in the packing through intermolecular N-H····O hydrogen bonding.

**Figure 10:**
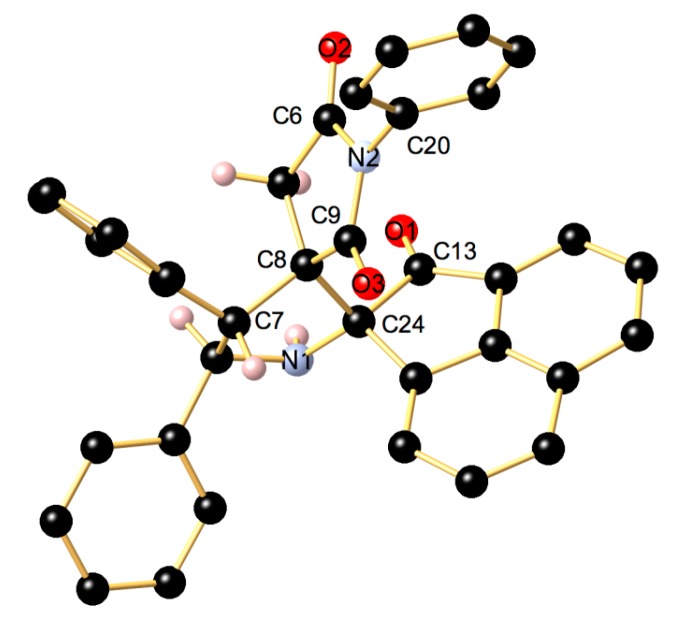
Ball and Sticks presentation of the molecular structure of **7f** in the crystal. For clarity, only stereochemically significant hydrogen atoms are shown. Selected bond lengths (Å) and angles (°):C24–N1 1.461(2), N1–C18 1.4784(18), C24–C8 1.587(2), C8–C9 1.525(2), C9–O3 1.2074(18), C9–N2 1.397(2), C6–N2 1.395(2), C6–O2 1.2093(18), C13–O1 1.2152(18); C7–C8–C24 99.71(12), C7–C8–C24 99.71(12), C24–C13–O1 123.67(14), C24–C8–C9 115.07(12), C8–C9–O3 127.87(14), C8–C9–N2 108.11(13), C9–N2–C6 113.11(13), N2–C6–O2 124.19(15).

The regio- and the stereochemical outcome of the cycloadditions were finally unambiguously ascertained by X-ray analysis of the crystal structure of cycloadducts **4b**, **4m**, **5m,** and **7f**, whose molecular structures are depicted in Figure 7, Figure 8, Figure 9 and Figure 10, respectively.

There are two independent molecules in the unit cell of **4b**. In the packing, two molecules are associated pairwise through two intermolecular N-H····O hydrogen interactions (*d*(N ····O)2.8622(15) Å) with a N1-H1····O1’ angle of 169.3(18)°. As illustrated in Figure 7, a supramolecular six-membered cycle is thus formed. In contrast, no intermolecular N-H····O hydrogen bonding is present in the solid-state structure of derivative **4m** (Figure 8).

A weak intermolecular N-H····O hydrogen interaction is present in the crystal structure of **5m**, albeit with weaker bonding (*d*(N····O) 3.064(3), *d*N-H····O 2.230(2) Å) compared to **4b**. Additionally, the O3 atom interacts weakly in an intramolecular manner with the H13 atom attached to C13 giving rise to a five-membered cycle (*d*C13-H····O13··2.391 Å) (Figure 9).

**Scheme 4:**
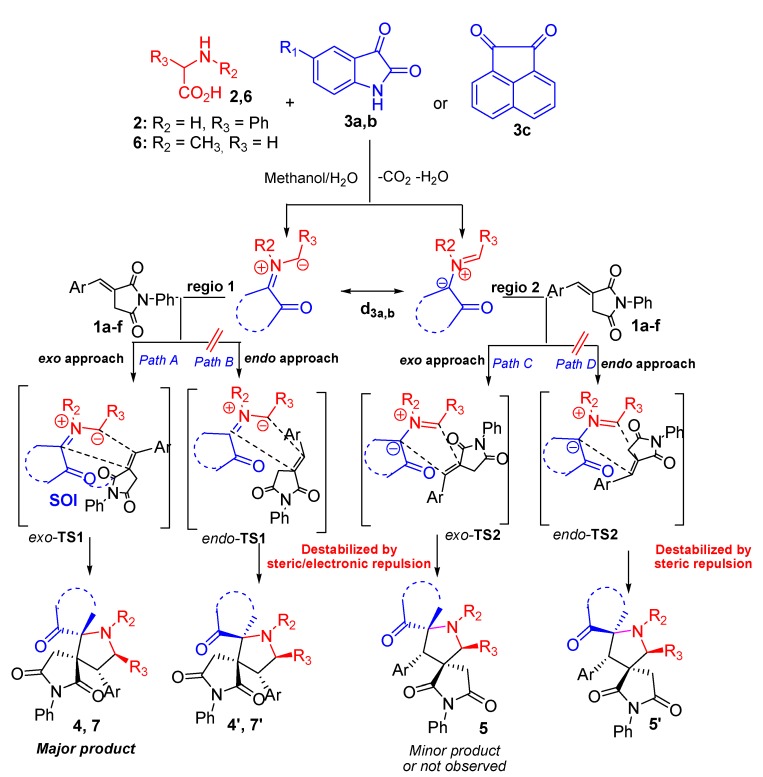
Proposed mechanism for the 1,3-dipolar cycloaddition of azomethine ylides **d_3,4_** with (*E)-*3-arylidene-1-phenylpyrrolidine-2,5-dione.

Elucidation of the four structures reveals (i) a *cis*-relationship between the carbonyl of the oxindole or acenaphthenequinone ring and the proton attached at C-10b0, and that (ii) the two carbonyl carbons of 1,2-diketone **3a**–**c** moieties and the enone part are in *trans-*relationship. Thus, the cycloadducts are formed through an *exo*-approach between the *(Z,E)*-dipole and enones **1** as outlined in Scheme 4. The cycloaddition proceeds with high *exo*-diastereoselectivity affording in each case only one diastereomer. 

On the basis of our experimental results and previous studies on the reaction mechanism [49,50,51,52,53], we propose in Scheme 4 a plausible mechanism for the regio- and stereoselective dispiropyrrolidine formation. The azomethine ylides **d_3_** are generated in situ by decarboxylative condensation of cyclic 1,2-diketones **3** with aminoacids **2** or **6**. The *(Z,E)*-dipole **d_3_** then undergoes a 1,3-dipolar cycloaddition reaction with the dipolarophile **1** in a regio- and stereoselective manner (path **A**). The formation of the *exo-*regioisomer **5** via path **A** is more favorable because of the presence of a secondary orbital interaction (SOI) [49,54,55], which occurs between the oxygen atom of the carbonyl of the diketone and the carbon atom of the carbonyl of the dipolarophile as shown in Scheme 4. Conversely, the formation of the other regioisomers or diastereoisomers is less favorable because of steric or electronic repulsion in their corresponding transition states. 

### 2.3. Cholinesterase Inhibitory Activities 

Some selected compounds were evaluated for their cholinesterase inhibitory activity against acetylcholinesterase (AChE) and butyrylcholinesterase (BuChE) enzymes. The principal role of AChE is termination of transmission at cholinergic synapses by rapid hydrolysis of the neurotransmitter acetylcholine (ACh) [56]. The findings are summarized in Table 4. IC_50_ determination was carried out only for those compounds having more than 50% inhibition at 5 μg/mL. The experimental details are provided in the Supplementary Materials.

In general, all tested compounds showed higher inhibition on AChE than BChE at similar concentration. Of the 17 synthesized compounds tested, five compounds exhibit an inhibition of more than 50%. Their respective IC_50_ values on AChE were in the range of 11.42 to 22.21 µM. The most active compound was **4n**, which was only five times less potent than the reference drug, galanthamine [57]. Compounds **4d** and **4e** bearing chloro substituents on the phenyl ring of the diketone **3a** skeleton display higher AChE inhibition than the methyl or methoxy substituents at *para*-position.

In contrast, compound **7b** incorporating an isatin **3a** motif, the methyl substituent at *para*-position exerted a higher AChE inhibition than in the case of the *p*-methoxy or *p*-chloro-substituted analogues **7c** and **7d**. In the case of compounds in series **7e**–**h** bearing an acenaphtoquinone **3c** moiety, the unsubstituted compound **7e** shows better AChE inhibition than the *para-*substituted derivatives (chloro, methyl, or methoxy).

**Figure 11:**
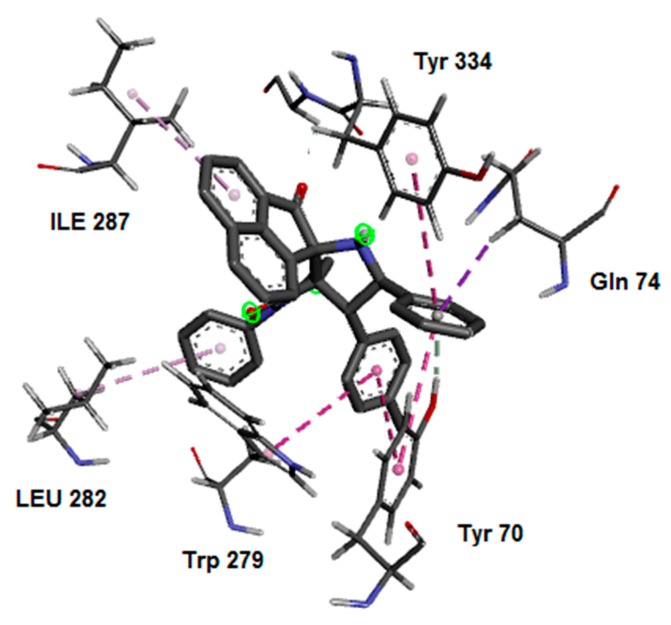
Binding interactions of **4n** with the acetylcholinesterase domain.

According to a molecular docking modelling study, the most potent AChE inhibitor, namely **4n**, is docked at the active site of the TcAChE enzyme allowing to investigate their orientation and binding interactions with the amino acid residues composing the active side channel. The molecular docking results for compound **4n** are summarized in Table 5. An adduct is formed via π–π interactions with the amino acid residues at the peripheral anionic site as shown in Figure 11, with a free energy of binding value of −10.69 kcal. This implies that compound **4n** inhibits the enzyme by blocking the entry site, thus preventing the substrate entry into the active gorge [58].

## 3. Materials and Methods 

### 3.1. Apparatus and General Information

NMR spectra were recorded with a Bruker-Spectrospin AC 300 spectrometer (Rheinstetten, Germany) operating at 300 MHz for ^1^H and 75 MHz for ^13^C. The following abbreviations were used to explain the multiplicities: bs = broad singlet, s = singlet, dd = doublet of doublet, d = doublet, t = triplet, m = multiplet. Elemental analyses were performed on a Perkin Elmer 2400 Series II Elemental CHNS analyzer (Waltham, MA, USA). Electrospray ionization (ESI) mass spectra were collected using a Q-TOF instrument supplied by WATERS (Agilent, Palo Alto, CA, USA). Materials: thin-layer chromatography (TLC): TLC plates (Merck, silica gel 60 F_254_ 0.2 mm 200 × 200 nm) (Darmstadt, Germany); substances were detected using UV light at 254 nm. 

### 3.2. General Procedure for Preparation of Cycloadducts ***4*** and ***5***

A mixture of **1** (3.0 mmol), 2-phenylglycine **2** (0.54 g, 3.6 mmol) and isatin derivatives **3** (3 mmol) was refluxed in MeOH/H_2_O (2:1 *v/v*, 10 mL) for 6h. After completion of the reaction (TLC), the solvent was removed under vacuum. The residue was chromatographed on silica gel employing ethylacetate-cyclohexane (3:7 *v/v*) as eluent to obtain the pure products **4** and **5**. 

(2R*,3S*,4R*,5R*)-spiro[2,3’]oxindole-spiro[3.3^″^]-N-phenylsuccinimide-4,5-diphenyl-pyrrolidine (**4a**). White solid (1.11 g, 74%); mp 161–163 °C; ^1^H-NMR: δ 2.55 (d, 1H, H-4˝, *J* = 18.9 Hz), 2.76 (d, 1H, H-4˝, *J* = 18.9 Hz), 4.34 (d, 1H, H-4, *J* = 9.3 Hz), 5.58 (d, 1H, H-5, *J* = 9.3 Hz), 6.77–6.81 (m, 3H, Ar-H), 6.96–7.01 (m, 1H, Ar-H), 7.17–7.36 (m, 10H, Ar-H), 7.44 (d, 2H, Ar-H; *J* = 7.2 Hz), 7.49–7.52 (m, 3H, Ar-H), 7.94 (bs, 1H, NH); ^13^C-NMR: δ 36.6, 60.7, 61.4, 66.5, 74.1, 109.6, 122.7, 126, 126.2, 126.8, 127.3, 128, 128.1, 128.4, 128.6, 129.8, 130.9, 136.5, 140.2, 140.6, 173.2, 178.7, 178.9; HRMS (ESI-TOF): calcd for C_32_H_25_N_3_O_3_ [M + H]^+^ 500.1974, found 500.1975.

(2R*,3S*,4R*,5R*)-spiro[2,3’]oxindole-spiro[4.3^″^]-N-phenylsuccinimide-3,5-diphenyl-pyrrolidine (**5a**). White solid (0.18 g, 13%); mp 111–113 °C; ^1^H-NMR: δ 3.05 (d, 1H, H-4˝, *J* = 18.3 Hz), 3.32 (d, 1H, H-4˝, *J* = 18.3 Hz), 4.73 (s, 1H, H-4), 5.72 (s, 1H, H-5), 6.39–6.42 (m, 2H, Ar-H), 6.68 (d, 1H, Ar-H; *J* = 7.8 Hz), 7.13–7.28 (m, 10H, Ar-H), 7.36–7.38 (m, 3H, Ar-H), 7.56–7.29 (m, 2H, Ar-H), 7.82 (d, 2H, Ar-H, *J* = 7.2 Hz); ^13^C-NMR: δ 40.8, 59, 59.9, 71.7, 72.8, 109.5, 123.3, 124.4, 126.1, 127.4, 128, 128.2, 128.6, 129.5, 129.7, 130.6, 131.5, 133.8, 137.5, 140.5, 174.1, 178.9, 180.3; HRMS (ESI-TOF): calcd for C_32_H_25_N_3_O_3_ [M + H]^+^ 500.1974, found 500.1973.

(2R*,3S*,4R*,5R*)-spiro[2,3’]oxindole-spiro[3.3^″^]-N-phenylsuccinimide-4-(p-methylphenyl)-5-phenyl-pyrrolidine (**4b**). White solid (1.05 g, 69%); mp 152–154 °C; ^1^H-NMR: δ 2.30 (s, 3H, CH_3_), 2.57 (d, 1H, H-4˝, *J* = 19 Hz), 2.75 (d, 1H, H-4˝, *J* = 19 Hz), 4.3 (d, 1H, H-4, *J* = 9.3 Hz), 5.55 (d, 1H, H-5, *J* = 9.3 Hz), 6.77-7.80 (m, 3H, Ar-H), 6.99 (t, 1H, Ar-H, *J* = 7.5 Hz), 7.14 (d, 2H, Ar-H, *J* = 7.8 Hz), 7.19–7.40 (m, 10H, Ar-H), 7.51 (d, 2H, Ar-H, *J* = 6.3 Hz), 8.03 (bs, 1H, NH); ^13^C-NMR: δ 20.5, 36.6, 60.4, 61.4, 66.3, 74.1, 109.6, 122.7, 125.9, 126.1, 126.2, 126.9, 127.2, 128, 128.1, 128.4, 129.3, 129.6, 129.7, 131, 133.4, 137, 140.6, 173.4, 178.8, 179; HRMS (ESI-TOF): calcd for C_33_H_27_N_3_O_3_ [M + H]^+^ 514.2131, found 514.2131.

(2R*,3S*,4R*,5R*)-spiro[2,3’]oxindole-spiro[4.3^″^]-N-phenylsuccinimide-3-(p-methylphenyl)-5-phenyl-pyrrolidine (**5b**). White solid (0.3g, 20%); mp 108–110 °C; ^1^H-NMR: δ 2.25 (s, 3H, CH_3_), 3.07 (d, 1H, H-4˝, *J* = 18.3 Hz), 3.31 (d, 1H, H-4˝, *J* = 18.3 Hz), 4.7 (s, 1H, H-4), 5.71 (s, 1H, H-5), 5.71 (d, 1H, Ar-H, *J* = 4.2 Hz), 6.37–7.40 (m, 2H, Ar-H), 6.69 (d, 1H, Ar-H, *J* = 7.2 Hz), 7-7.10 (m, 4H, Ar-H), 7.13–7.23 (m, 4H, Ar-H), 7.35–7.38 (m, 3H, Ar-H), 7.46 (bs, 1H, NH), 7.55–7.58 (m, 2H, Ar-H), 7.81 (d, 1H, Ar-H, *J* = 6.9 Hz); ^13^C-NMR: δ 20.4, 40.4, 58.5, 59.1, 71.2, 72.3, 109.1, 122.8, 125.6, 126.9, 127.7, 128.2, 128.9, 129.4, 131, 137.1, 137.3, 140, 173.8, 178.5, 180.1; HRMS (ESI-TOF): calcd for C_33_H_27_N_3_O_3_ [M + H^+^] 514.2131, found 514.2129.

(2R*,3S*,4R*,5R*)-spiro[2,3’]oxindole-spiro[3.3^″^]-N-phenylsuccinimide-4-(p-methoxylphenyl)-5-phenyl-pyrrolidine (**4c**). White solid (0.31 g, 60%); mp 133–135 °C; ^1^H-NMR: δ 2.64 (d, 1H, H-4˝, *J* = 19 Hz), 2.79 (d, 1H, H-4˝, *J* = 19 Hz), 3.82 (s, 3H, OCH_3_), 4.31 (d, 1H, H-4, *J* = 9.3 Hz), 5.52 (d, 1H, H-5, *J* = 9.3 Hz), 5.51–5.74 (m, 1H, Ar-H), 6.82–6.93 (m, 3H, Ar-H), 7.03 (d, 2H, Ar-H, *J* = 6.6 Hz), 7.05–7.07 (m, 1H, Ar-H), 7.22–7.42 (m, 9H, Ar-H), 7.55 (d, 3H, Ar-H, *J* = 9 Hz), 7.84 (bs, 1H, NH); ^13^C-NMR: δ 37.1, 55.2, 60.7, 61.9, 67.2, 74.6, 110.1, 114.5, 123.2, 126.4, 126.6, 126.7, 127.4, 127.8, 128.5, 128.7, 129, 130.3, 131.4, 141, 159.1, 174, 179.4, 179.6; HRMS (ESI-TOF): calcd for C_33_H_27_N_3_O_4_ [M + H]^+^ 530.2080, found 530.2079.

(2R*,3S*,4R*,5R*)-spiro[2,3’]oxindole-spiro[4.3^″^]-N-phenylsuccinimide-3-(p-methoxylphenyl)-5-phenyl-pyrrolidine (**5c**). White solid (0.13 g, 25%); mp 110–112 °C; ^1^H-NMR: δ 3.09 (d, 1H, H-4˝, *J* = 18.1 Hz), 3.26 (d, 1H, H-4˝, *J* = 18.1 Hz) 3.7 (s, 3H, OCH_3_), 4.66 (s, 1H, H-4), 5.7 (s, 1H, H-5), 6.35–7.39 (m, 2H, Ar-H), 6.73 (d, 1H, Ar-H, *J* = 9.3 Hz), 7.07 (d, 1H, Ar-H, *J* = 8.7 Hz), 7.11 (d, 1H, Ar-H, *J* = 9 Hz), 7.12–7.22 (m, 4H, Ar-H), 7.34–7.36 (m, 2H, Ar-H), 7.54–7.57 (m, 2H, Ar-H), 7.80 (d, 1H, Ar-H, *J* = 6.6 Hz); ^13^C-NMR: δ 40.8, 55.1, 59, 59.3, 71.8, 72.8, 74.6, 1;9.5, 114.1, 123.3, 124.4, 125.6, 126.1, 127.4, 128.3, 128.7, 129.4, 131.8, 141, 159.2, 174.3, 179.1, 180.5; HRMS (ESI-TOF): calcd for C_33_H_27_N_3_O_4_ [M + H]^+^ 530.2080, found 530.2080.

(2R*,3S*,4R*,5R*)-spiro[2,3’]oxindole-spiro[3.3^″^]-N-phenylsuccinimide-4-(p-chlorophenyl)-5-phenyl-pyrrolidine (**4d**). White solid (1.29 g, 80%); mp 150–152 °C; ^1^H-NMR: δ 2.57 (d, 1H, H-4˝, *J* = 18.9 Hz), 2.81 (d, 1H, H-4˝, *J* = 18.9 Hz), 4.34 (d, 1H, H-4, *J* = 9.3 Hz), 5.62 (d, 1H, H-5, *J* = 9.3 Hz), 6.80–6.84 (m, 3H, Ar-H), 7.04–7.07 (m, 1H, Ar-H), 7.28–7.47 (m, 12H, Ar-H), 7.51–7.56 (m, 3H, Ar-H), 8.2 (bs, 1H, NH); ^13^C-NMR: δ 36.6, 60.1, 61.2, 67, 74.2, 109.7, 122.8, 125.5, 126, 127.5, 128.1, 128.3, 128.5, 128.8, 129.9, 130.7, 131.3, 135.2, 140, 140.6, 173.1, 178.8; HRMS (ESI-TOF): calcd for C_32_H_24_ClN_3_O_3_ [M + H^+^] 534.1584, found 534.1587.

(2R*,3S*,4R*,5R*)-spiro[2,3’]oxindole-spiro[3.3^″^]-N-phenylsuccinimide-4-(m-chlorophenyl)-5-phenyl-pyrrolidine (**4e**). White solid (1.29 g, 82%); mp 160–162 °C; ^1^H-NMR: δ 2.55 (d, 1H, H-4˝, *J* = 19.2 Hz), 2.76 (d, 1H, H-4˝, *J* = 19.2 Hz), 4.65 (d, 1H, H-4, *J* = 9.9 Hz), 5.61 (d, 1H, H-5, *J* = 9.9 Hz), 6.83–7.02 (m, 3H, Ar-H), 7.27–7.30 (m, 7H, Ar-H), 7.37–7.42 (m, 4H, Ar-H), 7.50–7.58 (m, 4H, Ar-H), 8.42 (bs, 1H, NH); ^13^C-NMR: δ 36.9, 60.6, 61.4, 67.1, 74.5, 110.6, 123.4, 125.7, 126.5, 126.6, 127.5, 128.2, 128.6, 128.7, 128.9, 129,130.4, 130.7, 131.2, 135, 139.1, 141.2, 173.4, 178.8, 179.3; HRMS (ESI-TOF): calcd for C_32_H_24_ClN_3_O_3_ [M + H]^+^ 534.1584, found 534.1583.

(2R*,3S*,4R*,5R*)-spiro[2,3’]oxindole-spiro[3.3^″^]-N-phenylsuccinimide-4-(2-pyridyl)-5-phenyl-pyrrolidine (**4f**). White solid (1.29 g, 87%); mp 152–154 °C; ^1^H-NMR: 2.57 (d, 1H, H-4˝, *J* = 18.9 Hz), 2.81 (d, 1H, H-4˝, *J* = 18.9 Hz), 4.34 (d, 1H, H-4, *J* = 9 Hz), 5.57 (d, 1H, H-5, *J* = 9 Hz), 6.83-6.93 (m, 2H, Ar-H), 7.04 (t, 1H, Ar-H, *J* = 7.5 Hz), 7.24–7.43 (m, 11H, Ar-H), 7.52–7.58 (m, 4H, Ar-H), 7.97 (bs, 1H, NH); ^13^C-NMR: δ 37.1, 60.1, 61.7, 67.3, 74.7, 110.2, 123.3, 125.9, 126.5, 126.6, 127.3, 126.8, 128.1, 128.6, 128.9, 130.3, 130.5, 131.3, 135, 139.4, 140.4, 141, 173.4, 179.1, 179.2; Anal Calcd for C_31_H_24_N_4_O_3_: C, 74.38; H, 4.83; N, 11.19 %; found: C, 74.28; H, 4.8; N, 11.09%.

(2R*,3S*,4R*,5R*)-spiro[2,3’]oxindole-spiro[3.3^″^]-N-phenylsuccinimide-4-(2-furfuryl)-5-phenyl-pyrrolidine (**4g**). White solid (1.17 g, 80%); mp 152–154 °C; ^1^H-NMR: δ 2.56 (d, 1H, H-4˝, *J* = 18.9 Hz), 2.7 (d, 1H, H-4˝, *J* = 18.9 Hz), 4.44 (d, 1H, H-4, *J* = 9 Hz), 5.6 (d, 1H, H-5, *J* = 9 Hz), 6.7 (s, 1H, Ar-H), 6.7 (d, 1H, Ar-H, *J* = 3 Hz), 6.82 (d, 1H, Ar-H, *J* = 7.8 Hz), 6.95-7.03 (m, 3H, Ar-H), 7.28–7.42 (m, 8H, Ar-H), 7.59 (d, 1H, Ar-H, *J* = 7.5 Hz), 7.67 (d, 2H, Ar-H, *J* = 7.2 Hz), 8.37 (bs, 1H, NH); ^13^C-NMR: δ 35.9, 54.5, 60.5, 65, 74.4, 110, 110.5, 111, 123.2, 124.9, 125.9, 126.5, 127.6, 128.2, 128.7, 128.9, 129.1, 130.6, 140.1, 141.4, 142.6, 151.7, 173.8, 178.2, 179.2; Anal Calcd for C_30_H_23_N_3_O_4_: C, 73.61; H, 4.74; N, 8.58%; found: C, 73.52; H, 4.8; N, 8.66%.

(2R*,3S*,4R*,5R*)-spiro[2,3’]-4’-chlorooxindole-spiro[3.3^″^]-N-phenylsuccinimide-4,5-diphenyl-pyrrolidine (**4h***).* White solid (1.11 g, 69%); mp 152–154 °C; ^1^H-NMR: δ 2.62 (d, 1H, H-4˝, *J* = 19 Hz), 2.72 (d, 1H, H-4˝, *J* = 19 Hz), 4.35 (d, 1H, H-4, *J* = 9 Hz), 5.63 (d, 1H, H-5, *J* = 9.3 Hz), 6.8 (d, 1H, Ar-H, *J* = 8.1 Hz), 6.92 (d, 2H, Ar-H, *J* = 7.8 Hz), 7.18 (d, 2H, Ar-H, *J* = 7.5 Hz), 7.28–7.51 (m, 10H, Ar-H), 7.56–7.51 (m, 3H, Ar-H); ^13^C-NMR: δ 36.9, 60.5, 61.4, 66.6, 74.2, 111.5, 126.4, 127.2, 127.5, 128.1, 128.6, 128.8, 129, 129.2, 129.8, 129.9, 130.1, 130.5, 131.2, 133.3, 137.7, 139.7, 173.7, 179.3; Anal Calcd for C_32_H_24_ClN_3_O_3_: C, 71.97; H, 4.53; N, 7.87%; found: C, 72.01; H, 4.48; N, 8.91%.

(2R*,3S*,4R*,5R*)-spiro[2,3’]-4’-chlorooxindole-spiro[4.3^″^]-N-phenylsuccinimide-3,5-diphenyl-pyrrolidine (**5h**). White solid (0.24 g, 16%); mp 102–104 °C; ^1^H-NMR: δ 3.04 (d, 1H, H-4˝, *J* = 18.3 Hz), 3.31 (d, 1H, H-4˝, *J* = 18.3 Hz), 4.7 (s, 1H, H-4), 5.75 (s, 1H, H-5), 6.4–6.43 (m, 2H, Ar-H), 6.61 (d, 1H, Ar-H, *J* = 8.1 Hz), 7.15–7.58 (m, 11H, Ar-H), 7.69–7.48 (m, 4H, Ar-H); ^13^C-NMR: δ 40.5, 58.7, 59.9, 71.7, 72.6, 110.9, 125, 126.1, 127.4, 128, 128.3, 128.4, 128.7, 128.8, 128.9, 129.7, 130.5, 131, 131.3, 133.2, 136.6, 139.2, 173.8, 178.7, 179.7; Anal Calcd for C_32_H_24_ClN_3_O_3_: C, 71.97; H, 4.53; N, 7.87%; found: C, 71.87; H, 4.68; N, 7.80%.

(2R*,3S*,4R*,5R*)-spiro[2,3’]-4’-chlorooxindole-spiro[3.3^″^]-N-phenylsuccinimide-4-(p-methylphenyl)-5-phenyl-pyrrolidine (**4i**). White solid (1.2 g, 74%); mp 158–160 °C; ^1^H-NMR: δ 2.07 (s, 3H, CH_3_), 2.6 (d, 1H, H-4˝, *J* = 19.2 Hz), 2.73 (d, 1H, H-4˝, *J* = 19.2 Hz), 4.37 (d, 1H, H-4, *J* = 9 Hz), 5.64 (d, 1H, H-5, *J* = 9 Hz), 6.78 (d, 1H, Ar-H, *J* = 8.4 Hz), 6.9–6.93 (m, 2H, Ar-H), 7.25-7.46 (m, 9H, Ar-H), 7.49 (d, 2H, Ar-H, *J* = 7.2 Hz), 7.55–7.59 (m, 3H, Ar-H), 8.55 (bs, 1H, NH); ^13^C-NMR: δ 20.5, 36.5, 59.9, 61, 66.4, 73.9, 110.9, 125.9, 126.6, 127.5, 128.1, 128.3, 128.4, 128.7, 129.8, 130, 130.7, 139.2, 170.7, 173.2, 178.8; Anal Calcd for C_33_H_26_ClN_3_O_3_: C, 72.32; H, 4.78; N, 7.67 %; found: C, 72.39; H, 4.70; N, 7.71%.

(2R*,3S*,4R*,5R*)-spiro[2,3’]-4’-chlorooxindole-spiro[4.3^″^]-N-phenylsuccinimide-3-(p-methylphenyl)-5-phenyl-pyrrolidine (**5i**). White solid (0.21 g, 13%); mp 106–108 °C; ^1^H-NMR: δ 2.29 (s, 3H, CH_3_), 2.97 (d, 1H, H-4˝, *J* = 19.2 Hz), 3.21 (d, 1H, H-4˝, *J* = 19.2 Hz), 4.68 (s, 1H, H-4), 5.74 (s, 1H, H-5), 6.32–6.35 (m, 2H, Ar-H), 6.75 (d, 1H, Ar-H, *J* = 8.1 Hz), 7.05–7.09 (m, 2H, Ar-H), 7.15-7.18 (m, 5H, Ar-H), 7.27–7.29 (m, 5H, Ar-H), 7.48–7.51 (m, 2H, Ar-H); ^13^C-NMR: δ 21, 40.5, 58.6, 59.5, 71.6, 72.4, 111, 124.9, 126.1, 127.4, 128.4, 128.6, 128.8, 129.1, 129.6, 129.7, 130.4, 131, 131.3, 136.6, 138.2, 139.2, 174, 178.8, 179.7; Anal Calcd for C_33_H_26_ClN_3_O_3_: C, 72.32; H, 4.78; N, 7.67%; found: C, 72.27; H, 4.83; N, 7.73%.

(2R*,3S*,4R*,5R*)-spiro[2,3’]-4’-chlorooxindole-spiro[3.3^″^]-N-phenylsuccinimide-4-(p-methoxyphenyl)-5-phenyl-pyrrolidine (**4j**). White solid (1.41 g, 84%); mp 152–154 °C; ^1^H-NMR: δ 2.62 (d, 1H, H-4˝, *J* = 19.2 Hz), 2.72 (d, 1H, H-4˝, *J* = 19.2 Hz), 3.79 (s, 3H, OCH_3_), 4.28 (d, 1H, H-4, *J* = 9 Hz), 5.53 (d, 1H, H-5, *J* = 9 Hz), 6.67 (d, 1H, Ar-H, *J* = 8.4 Hz), 6.90 (d, 4H, Ar-H, *J* = 8.1 Hz), 7.23–7.31 (m, 4H, Ar-H), 7.34–7.45 (m, 5H, Ar-H), 7.53–7.56 (m, 3H, Ar-H); ^13^C-NMR: δ 36.5, 54.7, 60, 61.1, 66.6, 73.9, 110.9, 114, 125.8, 126.6, 126.7, 126.9, 127.5, 128.1, 128.2, 128.3, 128.7, 129.9, 130.7, 131, 158.7, 173.4, 178.9; Anal Calcd for C_33_H_26_ClN_3_O_4_: C, 70.27; H, 4.65; N, 7.45%; found: C, 70.2; H, 4.72; N, 7.55%.

(2R*,3S*,4R*,5R*)-spiro[2,3’]-4’-chlorooxindole-spiro[3.3^″^]-N-phenylsuccinimide-4-(p-chlorophenyl)-5-phenyl-pyrrolidine (**4k**). White solid (1.37 g, 81%); mp 145–147 °C; ^1^H-NMR: 2.56 (d, 1H, H-4˝, *J* = 19 Hz), 2.69 (d, 1H, H-4˝, *J* = 19 Hz), 4.38 (d, 1H, H-4, *J* = 9.3 Hz), 5.64 (d, 1H, H-5, *J* = 9.3 Hz), 6.84–6.93 (m, 3H, Ar-H), 7.28–7.78 (m, 14H, Ar-H), 8.52 (bs, 1H, NH); ^13^C-NMR: δ 36.7, 60, 61, 66.9, 73.9, 111.7, 125.7, 126.3, 127.2, 127.4, 128.4, 128.7, 128.9, 129, 129.2, 129.4, 130.8, 131.1, 131.7, 134.2, 139.6, 173.4, 179; Anal Calcd for C_32_H_23_Cl_2_N_3_O_3_: C, 67.61; H, 4.08; N, 7.39 %; found: C, 67.53; H, 4.16; N, 7.32%.

(2R*,3S*,4R*,5R*)-spiro[2,3’]-4’-chlorooxindole-spiro[3.3^″^]-N-phenylsuccinimide-4-(m-chlorophenyl)-5-phenyl-pyrrolidine (**4l**). White solid (1.5 g, 88%); mp 155–157 °C; ^1^H-NMR: δ 2.51 (d, 1H, H-4˝, *J* = 19.2 Hz), 2.66 (d, 1H, H-4˝, *J* = 19.2 Hz), 4.31 (d, 1H, H-4, *J* = 9 Hz), 5.57 (d, 1H, H-5, *J* = 9 Hz), 6.79 (d, 1H, Ar-H, *J* = 8.1 Hz), 6.86–6.9 (m, 2H, Ar-H), 7.22–7.41 (m, 10H, Ar-H), 7.51–7.53 (m, 4H, Ar-H); ^13^C-NMR: δ 36.8, 60.2, 61.1, 66.9, 74.3, 111.7, 126.3, 127.1, 127.4, 128.3, 128.6; 128.7, 128.9, 129, 129.2, 129.7, 130.4, 130.5, 130.7, 131.1, 135.1, 138.8, 139.7, 173.1, 179; C, 67.61; H, 4.08; N, 7.39%; found: C, 67.7; H, 4.15; N, 7.44%.

(2R*,3S*,4R*,5R*)-spiro[2,2’]acenaphthene-1’-one-spiro[3.3^″^]-N-phenylsuccinimide-4,5-diphenyl-pyrrolidine (**4m**). White solid (1.44 g, 90%); mp 150-152°C; ^1^H-NMR: δ 2.55 (q, 2H, H-4˝, *J* = 20 Hz), 4.46 (d, 1H, H-4, *J* = 9.3 Hz), 5.63 (d, 1H, H-5, *J* = 9.3 Hz), 6.72–7.75 (m, 2H, Ar-H), 7.25-7.74 (m, 15H, Ar-H), 7.85-7.92 (m, 2H, Ar-H), 7.98 (d, 1H, Ar-H, *J* = 6.9 Hz), 8.12 (d, 1H, Ar-H, *J* = 8.1 Hz); ^13^C-NMR: δ 36.4, 61.1, 62.1, 67.1, 77.7, 123.3, 126.1, 126.4, 127.4, 127.8, 128.5, 128.8, 128.9, 129.1, 129.2, 130.2, 130.3, 130.7, 130.8, 131.4, 132.4; 135.3, 135.9, 137.1, 141.2, 173.1, 179.9, 205.6; HRMS (ESI-TOF): calcd for C_36_H_27_N_2_O_3_ [M + H]^+^ 535.2022, found 535.2016.

(2R*,3S*,4R*,5R*)-spiro[2,3’]acenaphthene-1’-one-spiro[4.3^″^]-N-phenylsuccinimide-4,5-diphenyl-pyrrolidine (**5m**). White solid (1.44 g, 88%); mp 140–142 °C; ^1^H-NMR: δ 3.07 (d, 1H, H-4˝, *J* = 18.3 Hz), 3.41 (d, 1H, H-4˝, *J* = 18.3 Hz), 4.99 (s, 1H, H-3), 5.75 (s, 1H, H-5), 6.4–6.43 (m, 2H, Ar-H), 6.99–7.08 (m, 5H, Ar-H), 7.22-7.29 (m, 3H, Ar-H), 7.36–7.38 (m, 3H, Ar-H), 7.59–7.85 (m, 6H, Ar-H), 8.01–8.12 (m, 2H, Ar-H); ^13^C-NMR: δ 40.9, 59.9, 73.4, 75.4, 77.4, 120.4, 121.3, 125.2, 126.2, 127.5, 127.7, 128.2; 128.3, 128.6, 128.7, 128.2, 128.3, 128.6, 128.7, 129.1, 130.3, 130.7, 131.5, 131.6, 131.8, 134.2, 137.5, 139.5, 142.5, 174.3, 179.1, 207.7; HRMS (ESI-TOF): calcd for C_36_H_27_N_2_O_3_ [M + H]^+^ 535.2022, found 535.2020.

(2R*,3S*,4R*,5R*)-spiro[2,3’]acenaphthene-1’-one-spiro[3.3^″^]-N-phenylsuccinimide-4-(p-methylphenyl)-5-phenyl-pyrrolidine (**4n**). White solid (1.44 g, 88%); mp 140–242 °C; ^1^H-NMR: δ 2.37 (s, 3H, CH_3_), 2.6 (s, 2H, H-4˝), 4.47 (d, 1H, H-4, *J* = 9.3 Hz), 5.66 (d, 1H, H-5, *J* = 9.3 Hz), 6.77–6.80 (m, 2H, Ar-H), 7.22–7.40 (m, 8H, Ar-H), 7.45 (d, 2H, Ar-H, *J* = 7.8 Hz), 7.61–7.69 (m, 3H, Ar-H), 7.77–7.82 (m, 1H, Ar-H), 7.90–7.97 (m, 2H, Ar-H), 8.03 (d, 1H, Ar-H, *J* = 6.9 Hz), 8.17 (d, 1H, Ar-H, *J* = 8.4 Hz); ^13^C-NMR: δ 21, 36.4, 61.8, 62.1, 66.9, 77.7, 122.2, 123.3, 126, 126.4, 126.8, 127.4, 127.7, 128.5, 128.8, 128.9, 129.9, 130.1, 130.7, 130.9, 131.5, 132.3, 133.9, 136.1, 137.5, 141.2, 142.5, 173.5, 180, 205.4; HRMS (ESI-TOF): calcd for C_37_H_28_N_2_O_3_ [M + H]^+^ 549.2178, found 549.2183.

(2R*,3S*,4R*,5R*)-spiro[2,3’]acenaphthene-1’-one-spiro[3.3^″^]-N-phenylsuccinimide-4-(p-methoxyphenyl)-5-phenyl-pyrrolidine (**4o**). White solid (1.47 g, 87%); mp 151–153 °C; ^1^H-NMR: δ 2.55 (q, 2H, H-4˝), 4.39 (d, 1H, H-4, *J* = 9 Hz), 3.79 (s, 3H, OCH_3_), 5.57 (d, 1H, H-5, *J* = 9 Hz), 6.78–6.94 (m, 1H, Ar-H), 6.99–7.12 (m, 4H, Ar-H), 7.28–7.51 (m, 3H, Ar-H), 7.61–7.63 (m, 2H, Ar-H), 7.68–8.21(m, 10H, Ar-H); ^13^C-NMR: δ 39.3, 55.2, 61.6, 62, 67, 77.7, 114.6, 122.2, 123.3, 126.1, 126.4, 127.4, 127.7, 128.5, 128.6, 128.8, 129, 130.7, 130.8, 131.4, 131.5, 132.4, 136, 141.3, 142.5, 159.2, 173.6, 180.1, 205.6; HRMS (ESI-TOF): calcd for C_37_H_28_N_2_O_4_ [M + H]^+^ 565.2127, found 565.2127.

(2R*,3S*,4R*,5R*)-spiro[2,3’]acenaphthene-1’-one-spiro[3.3^″^]-N-phenylsuccinimide-4-(p-chlorophenyl)-5-phenyl-pyrrolidine (**4p**). White solid (1.53 g, 90%); mp 155–157 °C; ^1^H-NMR: δ 2.52 (d, 1H, H-4˝, *J* = 18.9 Hz), 2.6 (d, 1H, H-4˝, *J* = 18.9 Hz), 4.45 (d, 1H, H-4, *J* = 9.3 Hz), 5.59 (d, 1H, H-5, *J* = 9.3 Hz), 6.77–6.80 (m, 2H, Ar-H), 7.27–7.66 (m, 13H, Ar-H), 7.69–7.89 (m, 2H, Ar-H), 7.95–8.05 (m, 2H, Ar-H), 8.19 (d, 1H, Ar-H, *J* = 8.1 Hz); ^13^C-NMR: δ 36.4, 61.6, 61.8, 67.5, 77.8, 122.3, 123.7, 126.2, 126.3, 127.3, 127.9, 128.6, 128.8, 128.9, 129.1, 129.4, 129.5, 130.8, 131.2, 131.4, 131.7, 132.5, 133.8, 133.9, 135.7, 135.8, 140.8, 142.6, 172.5, 173.1, 179.9, 205.5; Anal Calcd for C_36_H_25_ClN_2_O_3_: C, 75.98; H, 4.43; N, 4.92%; found: C, 75.9; H, 4.38; N, 5.03 %.

(2R*,3S*,4R*,5R*)-spiro[2,3’]acenaphthene-1’-one-spiro[3.3^″^]-N-phenylsuccinimide-4-(m-chlorophenyl)-5-phenyl-pyrrolidine (**4q**). White solid (1.56 g, 92%); mp 135–137 °C; ^1^H-NMR: δ 2.5 (d, 1H, H-4˝, *J* = 19.2 Hz), 2.58 (d, 1H, H-4˝, *J* = 19.2 Hz), 4.45 (d, 1H, H-4, *J* = 9.3 Hz), 5.65 (d, 1H, H-5, *J* = 9.3 Hz), 6.76-6.82 (m, 2H, Ar-H), 7.27–7.41 (m, 8H, Ar-H), 7.51 (d, 1H, Ar-H, *J* = 7.2 Hz), 7.57–7.67 (m, 4H, Ar-H), 7.79 (d, 2H, Ar-H, *J* = 7.5 Hz), 7.79 (d, 1H, Ar-H, *J* = 7.5 Hz), 8.05 (d, 1H, Ar-H, *J* = 6.9 Hz), 8.18 (d, 1H, Ar-H, *J* = 8.1 Hz); ^13^C-NMR: δ 36.3, 61.5, 61.6, 67.3, 77.5, 122.6, 123.3, 125.4, 126.1, 126.4, 127.4, 128.1, 128.3, 128.5, 128.7, 128.8, 129, 130.4, 131.5, 131.2, 132.6, 135, 135.1, 139.2, 140.2, 142.6, 173.1, 179.9, 204.9; Anal Calcd for C_36_H_25_ClN_2_O_3_: C, 75.98; H, 4.43; N, 4.92%; found: C, 76.01; H, 4.46; N, 4.88%.

(2R*,3S*,4R*,5R*)-spiro[2,3’]acenaphthene-1’-one-spiro[3.3^″^]-N-phenylsuccinimide-4-(2-pyridyl)-5-phenyl-pyrrolidine (**4r**). White solid (1.5 g, 93%); mp 122–124 °C; ^1^H-NMR: δ 2.67 (d, 1H, H-4˝, *J* = 18.7 Hz), 2.77 (d, 1H, H-4˝, *J* = 18.7 Hz), 4.82 (d, 1H, H-4, *J* = 9.6 Hz), 5.81 (d, 1H, H-5, *J* = 9.6 Hz), 7.75 (d, 2H, Ar-H, *J* = 7.5 Hz), 7.02–7.32 (m, 7H, Ar-H), 7.66–7.78 (m, 6H, Ar-H), 7.89–7.99 (m, 3H, Ar-H), 8.16 (d, 1H, Ar-H, *J* = 8.1 Hz), 8.61 (d, 1H, Ar-H, *J* = 4.2 Hz); ^13^C-NMR: δ 36.3, 61.5, 61.6, 67.3, 77.5, 122.6, 123.3, 125.4, 126.1, 126.4, 127.4, 128.1, 128.3, 128.5, 128.7, 128.8, 129, 130.4, 131.5, 131.2, 132.6, 135, 135.1, 139.2, 140.2, 142.6, 173.1, 179.9, 204.9; Anal Calcd for C_35_H_25_N_3_O_3_: C, 78.49; H, 4.7; N, 7.85 %; found: C, 78.41; H, 4.75; N, 7.8%.

### 3.3. General Procedure for the Preparation of Cycloadducts ***7a**–**h***

A mixture of **1** (3.0 mmol), sarcosine **2** (3.6 mmol) and isatin **3a** or acenaphthenequinone **3c** (3 mmol) was refluxed in MeOH/H_2_O (2:1 v/v, 10 mL) for 6h. After completion of the reaction (TLC), the solvent was removed under vacuum. The residue was recrystallized from ethanol to obtain the pure product **7a**–**h**.

(2R*,3S*,4R*)-spiro[2,3’]oxindole-spiro[3.3^″^]-N-phenylsuccinimide-4-phenyl-N-methylpyrrolidine (**7a**). White solid (1.14 g, 85%); mp 152–154 °C; ^1^H-NMR: δ 2.29 (s, 3H, CH_3_), 2.5 (d, 1H, H-4˝, *J* = 18.9 Hz), 2.78 (d, 1H, H-4˝, *J* = 18.9 Hz), 3.62 (t, 1H, H-4), 4.07 (t, 1H, H-4), 4.54 (dd, 1H, H-5), 6.78–6.81 (m, 2H, Ar-H), 6.85 (d, 1H, Ar-H, *J* = 7.5 Hz), 7.02–7.07 (m, 1H, Ar-H), 7.28–7.44 (m, 8H, Ar-H), 7.5–7.53 (m, 2H, Ar-H), 7.61 (bs, 1H, NH); ^13^C-NMR: δ 34.6, 36.6, 49.3, 58.4, 61.3, 77.9, 109.4, 122.8, 124.6, 126.1, 127, 127.3, 128, 128.4, 128.6, 129.6, 131, 137.2, 140.9, 173.2, 176.9, 177.9; Anal Calcd for C_27_H_23_N_3_O_3_: C, 74.12; H, 5.3; N, 9.6%; found: C, 74.19; H, 5.25; N, 9.67%.

(2R*,3S*,4R*)-spiro[2,3’]oxindole-spiro[3.3^″^]-N-phenylsuccinimide-4-(p-methylphenyl)-N-methylpyrrolidine (**7b**). White solid (1.17 g, 88%); mp 135–137 °C; ^1^H-NMR: δ 2.28 (s, 3H, CH_3_), 2.35 (s, 3H, CH_3_), 2.51 (d, 1H, H-4˝, *J* = 18.7 Hz), 2.77 (d, 1H, H-4˝, *J* = 18.7 Hz), 3.58 (t, 1H, H-4), 4.05 (t, 1H, H-4), 4.51 (dd, 1H, H-5), 6.77–6.84 (m, 3H, Ar-H), 7–7.06 (m, 1H, Ar-H), 7.19–7.44 (m, 9H, Ar-H), 7.82 (bs, 1H, NH); ^13^C-NMR: δ 21, 35.1, 37, 49.5, 58.7, 61.7, 78.4, 109.9, 123.3, 125.1, 126.6, 127.6, 128.5, 128.8, 129.8, 129.9, 130.1, 131.6, 134.4, 137.6, 141.5, 173.7, 177.4, 178.4; Anal Calcd for C_28_H_25_N_3_O_3_: C, 74.48; H, 5.58; N, 9.31%; found: C, 74.41; H, 5.51; N, 9.4%.

(2R*,3S*,4R*)-spiro[2,3’]oxindole-spiro[3.3^″^]-N-phenylsuccinimide-4-(p-methoxyphenyl)-N-methylpyrrolidine (**7c**). White solid (1.35 g, 87%); mp 109–111 °C; ^1^H-NMR: δ 2.27 (s, 3H, CH_3_), 2.52 (d, 1H, H-4˝, *J* = 18.9 Hz), 2.75 (d, 1H, H-4˝, *J* = 18.9 Hz), 3.59 (t, 1H, H-4), 3.82 (s, 3H, OCH_3_), 4.01 (t, 1H, H-4), 4.48 (t, 1H, H-5), 6.78–6.84 (m, 3H, Ar-H), 6.92 (d, 2H, Ar-H, *J* = 8.7 Hz), 7.03 (t, 1H, Ar-H, *J* = 7.5 Hz), 7.29–7.42 (m, 7H, Ar-H), 7.56 (bs, 1H, NH); ^13^C-NMR: δ 35.1, 37, 49.2, 55.2, 58.9, 61.7, 78.4, 109.9, 114.5, 123.3, 125, 126.5, 127.7, 128.5, 128.8, 129.4, 130.1, 131.1, 131.6, 141.4, 159.3, 173.6, 177.2, 178.4; Anal Calcd for C_28_H_25_N_3_O_4_: C, 71.93; H, 5.39; N, 8.99%; found: C, 71.87; H, 5.3; N, 9.06%.

(2R*,3S*,4R*)-spiro[2,3’]oxindole-spiro[3.3^″^]-N-phenylsuccinimide-4-(p-chlorophenyl)-N-methylpyrrolidine (**7d**). White solid (1.14 g, 80%); mp 126–128 °C; ^1^H-NMR: δ 2.26 (s, 3H, CH_3_), 2.44 (d, 1H, H-4˝, *J* = 18.9 Hz), 2.75 (d, 1H, H-4˝, *J* = 18.9 Hz), 3.6 (t, 1H, H-4),3.98 (t, 1H, H-4), 4.48 (dd, 1H, H-5), 6.76–6.79 (m, 2H, Ar-H), 6.83 (d, 1H, Ar-H, *J* = 7.8 Hz), 7.04–7.38 (m, 7H, Ar-H), 7.45 (d, 2H, Ar-H, *J* = 8.7 Hz); ^13^C-NMR: δ 35, 37, 49, 59, 61.6, 78.4, 109.9, 123.4, 124.9, 126.5, 127.5, 128.5, 128.8, 129.2, 130.2, 131.5, 133.8, 136.2, 141.3, 173.3, 177.2, 178.1; Anal Calcd for C_27_H_22_ClN_3_O_3_: C, 68.71; H, 4.7; N, 8.9%; found: C, 68.81; H, 4.78; N, 8.86%.

(2R*,3S*,4R*)-spiro[2,3’] acenaphthene-1’-one-spiro[3.3^″^]-N-phenylsuccinimide-4-phenyl-N-methylpyrrolidine (**7e**). White solid (1.2 g, 85%); mp 115–117 °C; ^1^H-NMR: δ 2.24 (s, 3H, CH_3_), 2.46 (d, 1H, H-4˝, *J* = 18.7 Hz), 2.66 (d, 1H, H-4˝, *J* = 18.7 Hz), 3.73 (m, 1H, H-4), 4.19 (t, 1H, H-4), 4.68 (t, 1H, H-5), 6.69–6.72 (m, 2H, Ar-H), 7.30–7.82 (m, 11H, Ar-H), 7.93–7.97 (m, 2H, Ar-H), 8.17 (d, 1H, Ar-H, *J* = 8.1 Hz); ^13^C-NMR: δ 34.5, 35.7, 49.9, 58.6, 61.6, 120.5, 123.6, 125.4, 125.8, 125.9, 127.4, 127.8, 127.9, 128.3, 128.4, 128.5, 128.6, 129.5, 129.7, 130.2, 131, 131.1, 132, 134.5, 134.8, 137.2, 142.5, 172.8, 178.1, 206.6; Anal Calcd for C_31_H_24_N_2_O_3_: C, 78.79; H, 5.12; N, 5.93%; found: C, 78.85; H, 5.04; N, 6.01%.

(2R*,3S*,4R*)-spiro[2,3’]acenaphthene-1’-one-spiro[3.3^″^]-N-phenylsuccinimide-4-(p-methylphenyl)-N-methylpyrrolidine (**7f**). White solid (1.2 g, 83%); mp 102–104 °C; ^1^H-NMR: δ 2.21 (s, 3H, CH_3_), 2.37 (s, 3H, CH_3_), 2.45 (d, 1H, H-4˝, *J* = 18.9 Hz), 2.62 (d, 1H, H-4˝, *J* = 18.9 Hz), 3.68 (m, 1H, H-4), 4.14 (t, 1H, H-4), 4.62 (t, 1H, H-5), 6.66–6.69 (m, 2H, Ar-H), 7.22–7.32 (m, 5H, Ar-H), 7.42 (d, 2H, Ar-H, *J* = 8.1 Hz), 7.64–7.75 (m, 1H, Ar-H), 7.79 (d, 2H, Ar-H, *J* = 7.2 Hz), 7.90–7.94 (m, 2H, Ar-H), 8.15 (d, 1H, Ar-H, *J* = 8.1 Hz); ^13^C-NMR: δ 21, 35, 36.1, 50.1, 59, 62, 81, 121, 124.2, 125.8, 126.3, 128.3, 128.8, 129, 129.8, 130.2, 130.7, 131.5, 131.6, 132.4, 134.5, 135, 137.6, 143, 173.4, 178.6, 207.1; Anal Calcd for C_32_H_26_N_2_O_3_: C, 78.99; H, 5.39; N, 5.76%; found: C, 78.89; H, 5.31; N, 5.80 %.

(2R*,3S*,4R*)-spiro[2,3’]acenaphthene-1’-one-spiro[3.3^″^]-N-phenylsuccinimide-4-(p-methoxyphenyl)-N-methylpyrrolidine (**7g**). White solid (1.29 g, 87%); mp 120–122 °C; ^1^H-NMR: δ 2.22 (s, 3H, CH_3_), 2.47 (d, 1H, H-4˝, *J* = 18.9 Hz), 2.58 (d, 1H, H-4˝, *J* = 18.9 Hz), 3.72 (m, 1H, H-4), 3.84 (s, 3H, OCH_3_), 4.12 (t, 1H, H-4), 4.59 (t, 1H, H-5), 6.69–6.73 (m, 2H, Ar-H), 6.97 (d, 2H, Ar-H, *J* = 8.7 Hz), 7.27-7.38 (m, 3H, Ar-H), 7.27-7.38 (m, 2H, Ar-H), 7.41-7.53 (m, 3H, Ar-H), 7.66–7.80 (m, 3H, Ar-H), 7.92–7.97 (m, 2H, Ar-H), 8.18 (d, 1H, Ar-H, *J* = 8.1 Hz); ^13^C-NMR: δ 34.6, 35.6, 49.4, 54.8, 58.8, 61.4, 80.4, 114.2, 120.6, 123.6, 125.5, 125.9, 126, 127.9, 128, 128.4, 128.6, 128.9, 130.2, 130.6, 130.8, 130.9, 131.7, 132.1, 134.2, 142.6, 158.7, 173.1, 178.4, 206.9; HRMS (ESI-TOF): calcd for C_32_H_26_N_2_O_4_ [M + H]^+^ 503.1971, found 503.1975.

(2R*,3S*,4R*)-spiro[2,3’]acenaphthene-1’-one-spiro[3.3^″^]-N-phenylsuccinimide-4-(p-chlorophenyl)-N-methylpyrrolidine (**7h**). White solid (1.2 g, 80%); mp 125–126 °C; ^1^H-NMR: δ 2.22 (s, 3H, CH_3_), 2.4 (d, 1H, H-4˝, *J* = 18.9 Hz), 2.59 (d, 1H, H-4˝, *J* = 18.9 Hz), 3.73 (t, 1H, H-4), 4.11 (t, 1H, H-4), 4.61 (t, 1H, H-5), 6.68–6.72 (m, 2H, Ar-H), 7.28–7.37 (m, 2H, Ar-H), 7.42(d, 2H, Ar-H, *J* = 8.4 Hz), 7.53 (d, 2H, Ar-H, *J* = 8.4 Hz), 7.61–7.82 (m, 3H, Ar-H), 7.93–7.99 (m, 3H, Ar-H), 8.20 (d, 1H, Ar-H, *J* = 8.1 Hz); ^13^C-NMR: δ 35, 36.2, 49.7, 59.4, 61.8, 80.9, 121.2, 124, 126.1, 126.3, 128.5, 128.6, 128.9, 129.4, 130.7, 131.2, 131.3, 131.5, 132.7, 133.8, 134.5, 136.3, 143.1, 173.2, 178.7, 207.5; Anal Calcd for C_31_H_23_ClN_2_O_3_: C, 73.44; H, 4.57; N, 5.53%; found: C, 73.39; H, 4.62; N, 5.47%.

### 3.4. Crystal Structure Determinations

A suitable crystal of **4b**, **4m**, **5m,** and **7f** was selected and mounted on an Xcalibur, Sapphire3 diffractometer (Abingdon, UK). The crystals were kept at 150(2) K during data collection. Using Olex2 [59], the structures were solved with the ShelXS [60], structure solution program using direct methods and refined with ShelXL [60], refinement package using least squares minimization. Data (excluding structure factors) for the structures of **4b**, **4m**, **5m,** and **7f** have been deposited at CCDC with deposition numbers 1958732, 1958733, 1958730, and 1958731. These data may be obtained free of charge from CCDC through www.ccdc.cam.ac.uk/data_request/cif. The main crystallographic data together with refinement details are summarized in Table 6.

### 3.5. Cholinesterase Inhibitory Assay

Acetylthiocholine iodide (ATCl), acetylcholinesterase (AChE, from electric eel), butyrylcholinesterase (BChE, from equine serum), *S-*butyrylthiocholine chloride, and 5, 50-dithiobis(2-nitrobenzoicacid) (Ellman’s reagent, DTNB) were purchased from Sigma–Aldrich (Kuala Lumpur, Malaysia).

Cholinesterase enzyme inhibitory potential of the test samples was determined following the method of Khaw et al. (2014) with slight modifications on the vehicle used. Briefly, test samples and galantamine were prepared in DMSO at the initial concentration of 0.5 mg/mL. The final concentration of DMSO in reaction mixture was 1%. At this concentration, DMSO has no inhibitory effect on both AChE and BChE enzymes. For AChE inhibitory assay, 140 µL of 0.1 M sodium phosphate buffer (pH 8) was added to a 96 wells microplate followed by 20 µL of test samples and 20 µL of 0.09 units/mL AChE enzyme. Then, 10 µL of 10 mM DTNB was added into each well followed by 10 µL of 14 mM of acetylthiocholine iodide. The absorbance of the colored end product was measured using Tecan Infinite 200 Pro Microplate Spectrophotometer at 412 nm for 30 min. Each test was conducted in triplicate. For BChE inhibitory assay, the same procedures were applied as AChE except for the use of enzyme and substrate, which were BChE from equine serum and S-butyrylthiocholine chloride. Absorbencies of the test samples were corrected by subtracting the absorbance of their respective blank (test samples in DMSO with substrate and DTNB, but without enzyme). A set of five concentrations was used to estimate the 50% inhibitory concentration (IC_50_) for the compounds showing more than 50% inhibition at 5µg/mL concentration.

### 3.6. Molecular Docking of Compound ***4n***

Molecular docking for **4m** was performed using Autodock 3.0.5 (La Jolla, CA, USA) along with AutoDockTools (ADT) [61]. **4m** was built using Hyperchem 8 and energy minimization was performed with convergence criterion of 0.05 kcal/molA. Crystal structure of AChE from *Torpedo californica* in complex with galanthamine was obtained from Protein Data Bank with PDB ID: 1W6R [62]. The protein was edited using ADT to remove all water molecules and all hydrogen atoms were added. Nonpolar hydrogen atoms and lone pairs were then merged, and each atom was assigned with Gasteiger partial charges. A grid box of 41 × 53 × 41 points, with a spacing of 0.375A° was positioned at the center of active-site gorge. One hundred independent dockings were carried out for each docking experiment. The lowest docked energy of each conformation in the most populated cluster was selected. Analysis and visualization of the docking results was done using BIOVIA Discovery Studio visualizer.

## 4. Conclusions

To conclude, we have developed an efficient and practical strategy for the multicomponent 1,3-dipolar cycloaddition of *(E)-*3-benzylidene-1-phenyl-succinimides, 1,2-cyclic diketones, and diverse *α-*aminoacids. This protocol enables the convenient synthesis of a novel class of dispiropyrrolidines fused succinimide in good to high yields with high diastereoselectivity. Noteworthy, some of the synthetized compounds showed good AChE inhibition. Compound **4n** bearing a methyl substituent at the *para*-position of the phenyl ring exhibited the most potent AChE inhibition. The molecular docking simulation of this compound disclosed its interesting binding interaction to the active site channel of AChE enzymes. 

## Figures and Tables

**Table 1:** Optimization of the reaction conditions for the 1,3-dipolar cycloaddition of dipolarophile **1a** across 2-phenylglycine **2** and isatin **3a** ^a^.

Entry	Solvent	T (°C)	Time (h)	rr (4a:5a) ^b^	Yield (%) ^c^
1	H_2_O	rt	24	70:30	25
2	PhCH_3_	rt	24	70:30	29
3	CH_3_CN	rt	24	70:30	35
4	CH_3_CN	reflux	24	78:22	55
5	EtOH	reflux	12	78:22	70
6	*i*PrOH	reflux	10	70:30	65
7	MeOH	reflux	6	78:22	77
8	MeOH/H_2_O 1:1	reflux	8	80:20	65
**9**	**MeOH/H_2_O 2:1**	**reflux**	**6**	**85:15**	**87**

^a^ The reaction was carried out in 0.5 mmol scale in solvent (10 mL), and the ratio of **1a**/**2**/**3a** is 1:1.2:1. ^b^ The regioisomeric ratio (rr) was determined by the isolated yields of **4a** and **5a**. ^c^ Combined yield of isolated **4a** and **5a**.

**Table 2:** Synthesis of dispiropyrrolidines **4** ^a^.

Entry	1,2-diketone	Ar	Comp.	rr (4:5) ^b^	Yield (%) ^d^
1	3a	C_6_H_5_	**4a** + **5a**	85:15	87
2	3a	*p-*MeC_6_H_4_	**4b** + **5b**	78:22 ^c^	89
3	3a	*p-*MeOC_6_H_4_	**4c** + **5c**	70:30 ^c^	85
4	3a	*p-*ClC_6_H_4_	**4d**	100:00	80
5	3a	*m-*ClC_6_H_4_	**4e**	100:00	82
6	3a	2-pyridyl	**4f**	100:00	87
7	3a	2-furfuryl	**4g**	100:00	80
8	3b	C_6_H_5_	**4h** + **5h**	81:19 ^c^	85
9	3b	*p-*MeC_6_H_4_	**4i** + **5i**	85:15	87
10	3b	*p-*MeOC_6_H_4_	**4j**	100:00	84
11	3b	*p-*ClC_6_H_4_	**4k**	100:00	81
12	3b	*m-*ClC_6_H_4_	**4l**	100:00	88
13	3c	C_6_H_5_	**4m** + **5m**	87:13	90
14	3c	*p-*MeC_6_H_4_	**4n**	100:00	88
15	3c	*p-*MeOC_6_H_4_	**4o**	100:00	87
16	3c	*p-*ClC_6_H_4_	**4p**	100:00	90
17	3c	*m-*ClC_6_H_4_	**4q**	100:00	92
18	3c	2-pyridyl	**4r**	100:00	93

^a^ The reaction was carried out in 3 mmol scale in MeOH/H_2_O (10 mL) at reflux for 6 h, and the ratio of **1**/**2**/**3** is 1:1.2:1. ^b^ Unless otherwise noted, the regioisomeric ratio (rr) was determined by the isolated yields of **4** and **5**. ^c^ Determined by ^1^H-NMR analysis of the crude product. ^d^ Combined yields of isolated **4** and **5**. ^e^ Failed to separate compound **5**.

**Table 3:** Synthesis of dispiropyrrolidines **7.**

Entry	Comp.	1,2-diketone	Ar	Yield (%)
1	7a	3a	C_6_H_5_	85
2	7b	3a	*p-*MeC_6_H_4_	88
3	7c	3a	*p-*MeOC_6_H_4_	87
4	7d	3a	*p-*ClC_6_H_4_	80
5	7e	3c	C_6_H_5_	85
6	7f	3c	*p-*MeC_6_H_4_	83
7	7g	3c	*p-*MeOC_6_H_4_	87
8	7h	3c	*p-*ClC_6_H_4_	80

**Table 4:** AChE and BChE inhibitory activities of spiroheterocyclic hybrids **4** and **7.**

Compound	Diketone	Aminoacid	Ar	% of Inhibition at 5 µg/mL		AChE Inhibition, IC_50_	
				AChE	BChE	µg/mL	µM
**4a**	3a	2	C_6_H_5_	41.95 ± 0.015	29.46 ± 0.015	-	-
**4b**	3a	2	*p-*MeC_6_H_4_	39.13 ± 0.014	8.65 ± 0.011	-	-
**4c**	3a	2	*p-*MeOC_6_H_4_	42.34 ± 0.001	23.33 ± 0.002	-	-
**4d**	3a	2	*p-*ClC_6_H_4_	50.09 ± 0.006	31.36 ± 0.032	11.75 ± 0.58	22.21 ± 0.88
**4e**	3a	2	*m-*ClC_6_H_4_	53.57 ± 0.007	48.59 ± 0.001	9.38 ± 0.46	17.56 ± 0.70
**4m**	3c	2	C_6_H_5_	49.10 ± 0.011	17.98 ± 0.014	-	-
**4n**	3c	2	*p-*MeC_6_H_4_	70.56 ± 0.002	14.20 ± 0.000	6.10 ± 0.30	11.42 ± 0.45
**4o**	3c	2	*p-*MeOC_6_H_4_	35.55 ± 0.023	21.37 ± 0.036	-	-
**4p**	3c	2	*p-*ClC_6_H_4_	42.55 ± 0.007	20.93 ± 0.026	-	-
**7a**	3a	2	C_6_H_5_	30.39 ± 0.017	4.16 ± 0.011	-	-
**7b**	3a	6	*p-*MeC_6_H_4_	51.25 ± 0.015	20.62 ± 0.020	8.27 ± 0.41	16.02 ± 0.64
**7c**	3a	6	*p-*MeOC_6_H_4_	42.78 ± 0.023	11.00 ± 0.015	-	-
**7d**	3a	6	*p-*ClC_6_H_4_	40.24 ± 0.002	14.15 ± 0.012	-	-
**7e**	3c	6	C_6_H_5_	60.00 ± 0.009	24.72 ± 0.000	9.65 ± 0.48	20.44 ± 0.81
**7f**	3c	6	*p-*MeC_6_H_4_	32.10 ± 0.006	18.89 ± 0.020	-	
**7g**	3c	6	*p-*MeOC_6_H_4_	43.00 ± 0.001	7.29 ± 0.013	-	
**7h**	3c	6	*p-*ClC_6_H_4_	38.10 ± 0.014	27.11 ± 0.011	-	
**Galantamine**						0.60 ± 0.09	2.09 ± 0.11

Data presented as mean ± SD (*n* = 3).

**Table 5:** Molecular docking of **4n.**

Compound	Enzyme	Free Energy of Binding (kcal)	Residue	Active Site	Type of Interaction	Distance
**4n**	AChE	−10.69	TRP 279 TYR 70 TYR 334	Peripheral anionic site	π-π	5.13 5.59 4.95 5.50

**Table 6:** Crystal data collection and structure refinement of **4b, 4j, 5j,** and **7b**.

Compound/Formula	4b C_66_H_54_N_6_O_6_	4j C_36_H_26_N_3_O_3_	5j C_36_H_26_N_2_O_3_	7b C_32_H_26_N_2_O_3_
**Formula weight**	1027.15	534.59	534.59	486.55
**Temperature/K**	173(2)	150(2)	150(2)	150(2)
**Wavelength/Å**	0.71073	0.71073	0.71073	0.71069
**Crystal system**	Triclinic	Triclinic	Orthorhombic	Monoclinic
**Space group**	P-1	P-1	P2_1_2_1_2_1_	I2/a
***a/*** **Å**	12.6005(3)	10.2431(6)	11.2244(6)	13.5410(11)
***b/*** **Å**	14.8515(4)	11.2174(6)	14.9261(6)	16.0780(11)
***c/*** **Å**	16.7480(4)	12.9289(6)	15.9902(8)	25.5975(19)
***α***	69.932(2)°	84.107(4)°	90°	90°
***β***	71.396(2)°	84.571(4)°	90°	96.332(7)°
***γ***	76.6788(19)°	33. 246(6)°	90°	90°
**Volume/Å^3^**	2764.18(13)	1317.53(14)	2678.9(2)	5538.9(7)
***Z***	2	2	4	8
**ρ_calc_ g/cm^3^**	1.234	1.3484	1.325	1.167
**Absorp. coefficient/mm^−1^**	0.080	0.086	0.085	0.075
***F*** **(000)**	1080.0	1560.0	1120.0	2048.0
**Crystal size/mm^3^**	0.15 × 0.10 × 0.16	0.12 × 0.087 × 0.05	0.32 × 0.05 × 0.04	0.25 × 0.06 × 0.03
**2 Theta range for data collection/°**	4.83 to 58.43	4.46 to 54.09	4.434 to 55.994	4.868 to 58.304
**Index ranges**	−17 ≤ h ≤ 17,	−13 ≤ h ≤ 13,	−13 ≤ h ≤ 14,	−17 ≤ h ≤ 17,
	−20 ≤ k ≤ 20,	−14 ≤ k ≤ 14,	−19 ≤ k ≤ 19,	−21 ≤ k ≤ 21,
	−22 ≤ l ≤ 22	−16 ≤ l ≤ 16	−21 ≤ l ≤ 21	−34 ≤ l ≤ 34
**Reflections collected**	144222	27647	39373	31906
**Independent reflections**	14030 [*R*(int) = 0.0373]	5732 [R(int) = 0.0502]	6389 [R(int) = 0.0516]	6779 [R(int) = 0.0848]
**Refinement method**	Full-matrix least-squares on *F*^2^	Full-matrix least-squares on *F*^2^	Full-matrix least-squares on *F*^2^	Full-matrix least-squares on *F*^2^
**Data/restraints/parameters**	1430/36/7214	5732/1/3745	6389/0/374	6779/36/336
**Goodness-of-fit on *F^2^***	1.035	1.040	1.036	1.008
**Final *R* indices [*I* > 2sigma(I)]**	*R*1 = 0.0507,	*R*1 = 0.0461,	*R*1 = 0.0441,	*R*1 = 0.0480,
	w*R*2 = 0.1342	w*R*2 = 0.0883	w*R*2 = 0.0804	w*R*2 = 0.1172
***R*** **indices (all data)**	*R*1 = 0.0666,	*R*1 = 0.0734,	*R*1 = 0.0616,	*R*1 = 0.01227,
	w*R*2 = 0.1450	w*R*2 = 0.0986	w*R*2 = 0.0863	w*R*2 = 0.1374
**Largest diff. peak and hole/e. Å^−3^**	0.74 and −0.1450	0.24 and −0.25	0.18 and −0.27	0.22 and −0.25

## Supplementary Materials

The following are available online, Figures S1–S64: ^1^H and ^13^C-NMR spectra, Additional information on Cholinesterase inhibitory assay, Experimental details concerning the Molecular Docking investigation of compound **4n**.

## References

[1] Marson C.M. (2011). New and Unusual Scaffolds in Medicinal Chemistry. Chem. Soc. Rev..

[2] Zheng Y.C., Tice M., Singh S.B. (2014). The Use of Spirocyclic Scaffolds in Drug Discovery. Bioorg. Med. Chem. Lett..

[3] Kotha S., Panguluri N.R., Ali R. (2017). Design and Synthesis of Spirocycles. Eur. J. Org. Chem..

[4] El-Sharief A.M.S., Ammar Y.A., Belal A., El-Sharief M.A.M.S., Mohamed Y.A., Mehany A.B.M., Elhag Ali G.A.M., Ragab A. (2019). Design, Synthesis, Molecular Docking and Biological Activity Evaluation of Some Novel Indole Derivatives as Potent Anticancer Active Agents and Apoptosis Inducers. Bioorg. Chem..

[5] Lotfy G., El Sayed H., Said M.M., Aziz Y.M.A., Al-Dhfyan A., Al-Majid A.M., Barakat A. (2018). Regio- and Stereoselective Synthesis of New Spirooxindoles *via* 1,3-Dipolar Cycloaddition Reaction: Anticancer and Molecular Docking Studies. J. Photochem. Photobiol. B Biol..

[6] Bouhenna M.M., Orlikova B., Talhi O., Schram B., Pinto D.C.G.A., Taibi N., Bachari K., Diederich M., Silva A.M.S., Mameri N. (2017). Anti-proliferative, Cytotoxic and NF-ĸB Inhibitory Properties of Spiro(Lactone-Cyclohexanone) Compounds in Human Leukemia. Anticancer Res..

[7] Kumar R.S., Almansour A.I., Arumugam N., Mohammad F. (2019). Design, Synthesis and In Vitro Mechanistic Investigation of Novel Hexacyclic Cage-Like Hybrid Heterocycles. Molecules.

[8] Galvez J., Polo S., Insuasty B., Gutierrez M., Cáceres D., Alzate-Morales J.H., De-la-Torre P., Quiroga J. (2018). Design, Facile Synthesis, and Evaluation of Novel Spiro- and Pyrazolo[1,5-*c*]quinazolines as Cholinesterase Inhibitors: Molecular Docking and MM/GBSA Studies. Comput. Biol. Chem..

[9] Arumugam N., Almansour A.I., Kumar R.S., Kotresha D., Saiswaroop R., Venketesh S. (2019). Dispiropyrrolidinyl-piperidone Embedded Indeno[1,2-*b*]quinoxaline Heterocyclic Hybrids: Synthesis, Cholinesterase Inhibitory Activity and their Molecular Docking Simulation. Bioorg. Med. Chem..

[10] Yang M.-C., Peng C., Huang H., Yang L., He X.-H., Huang W., Cui H.-L., He G., Han B. (2017). Organocatalytic Asymmetric Synthesis of Spiro-oxindole Piperidine Derivatives That Reduce Cancer Cell Proliferation by Inhibiting MDM2–p53 Interaction. Org. Lett..

[11] Hegde S.G., Koodlur L., Narayanarao M. (2019). Regioselective Synthesis and Biological Evaluation of Novel Dispiropyrrolidine Derivatives v*ia one-pot* four-Component Reaction. Synth. Commun..

[12] Redkin R.G., Syumka E.I., Shemchuk L.A., Chernykh V.P. (2017). Synthesis and antimicrobial activity of Bis-Derivatives of 3a′, 6a′Dihydro-2’H-Spiro[Indole-3,1’-Pyrrolo[3,4-c]Pyrrole]-2,4’,6’(1H, 3’H, 5’H)-Trione. J. App. Pharm. Sci..

[13] Almansour A.I., Arumugam N., Kumar R.S., Al-thamili D.M., Periyasami G., Ponmurugan K., Al-Dhabi N.A., Perumal K., Premnath D. (2019). Domino Multicomponent Approach for the Synthesis of Functionalized Spiro-Indeno[1,2-b]quinoxaline Heterocyclic Hybrids and Their Antimicrobial Activity, Synergistic Effect and Molecular Docking Simulation. Molecules.

[14] Nakagawa T., Ubukata T., Yokoyama Y. (2018). Chirality and stereoselectivity in photochromic reactions. J. Photochem. Photobiol. C Photochem. Rev..

[15] Coudret C., Chernyshev A.V., Metelitsa A.V., Micheau J.C. (2017). New Trends in Spiro-compounds Photochromic Metals Sensors: Quantitative Aspects. Photon-Working Switches.

[16] Takase K., Noguchi K., Nakano K. (2018). [1]Benzothiophene-Fused Chiral Spiro Polycyclic Aromatic Compounds: Optical Resolution, Functionalization, and Optical Properties. J. Org. Chem..

[17] Gao K., Xu B., Hong C.S., Shi X.L., Liu H.B., Li X.S., Xie L.H., Jen A.K.Y. (2018). Di-Spiro-Based Hole-Transporting Materials for Highly Efficient Perovskite Solar Cells. Adv. Energy Mater..

[18] Carreira E.M., Fessard T.C. (2014). Four-Membered Ring-Containing Spirocycles: Synthetic Strategies and Opportunities. Chem. Rev..

[19] Gollner A., Rudolph D., Arnhof H., Bauer M., Blake S.M., Boehmelt G., Cockroft X.-L., Dahmann G., Ettmayer P., Gerstberger T. (2016). Discovery of Novel Spiro[3H-indole-3,2′-pyrrolidin]-2(1H)-one Compounds as Chemically Stable and Orally Active Inhibitors of the MDM2−p53 Interaction. J. Med. Chem..

[20] Popowicz G.M., Czarna A., Wolf S., Wang K., Wang W., Dömling A., Holak T.A. (2010). Structures of Low Molecular Weight Inhibitors Bound to MDMX and MDM2 Reveal New Approaches for p53-MDMX/ MDM2 Antagonist Drug Discovery. Cell Cycle.

[21] Patchett A.A., Nargund R.P., Doherty A.M. (2000). Privileged Structures an Update. Annual Reports in Medicinal Chemistry.

[22] Aguilar A., Lu J., Liu L., Du D., Bernard D., McEachern D., Przybranowski S., Li X., Luo R., Wen B. (2017). Discovery of 4-((3′R,4′S,5′R)-6″-Chloro-4′-(3-chloro-2-fluorophenyl)-1′-ethyl-2″-oxodispiro[cyclohexane-1,2′-pyrrolidine-3′,3″-indoline]-5′-carboxamido)bicyclo[2.2.2]octane-1-carboxylic Acid (AA-115/APG115): A Potent and Orally Active Murine Double Minute 2 (MDM2) Inhibitor in Clinical Development. J. Med. Chem..

[23] Gallifordand C.V., Scheidt A. (2007). Pyrrolidinyl-Spirooxindole Natural Products as Inspirations for the Development of Potential Therapeutic Agents. Angew. Chem. Int. Ed. Engl..

[24] Arun Y., Bhaskar G., Balachandran C., Ignacimuthu S., Perumal P.T. (2013). Facile One-Pot Synthesis of Novel Dispirooxindole-Pyrrolidine Derivatives and Their Antimicrobial and Anticancer Activity Against A549 Human Lung Adenocarcinoma Cancer Cell Line. Bioorg. Med. Chem. Lett..

[25] Wei A.C., Ali M.A., Yoon Y.K., Ismail R., Choon T.S., Kumar R.S. (2013). A Facile Three-Component [3+2]-Cycloaddition for the Regioselective Synthesis of Highly Functionalised Dispiropyrrolidines Acting as Antimycobacterial Agents. Bioorg. Med. Chem. Lett..

[26] Haddad S., Boudriga S., Porzio F., Soldera A., Askri M., Sriram D., Yogeeswari P., Knorr M., Rousselin Y., Kubicki M.M. (2014). Synthesis of Novel Dispiropyrrolothiazoles by Three-Component 1,3-Cipolar Cycloaddition and Evaluation of Their Antimycobacterial Activity. RSC Adv..

[27] Arumugama N., Almansoura A.I., Kumara R.S., Altafa M., Padmanabanc R., Sureshbabuc P., Angamuthuc G., Kotreshad D., Manohare T.S., Venketeshe S. (2018). Spiropyrrolidine/spiroindolizino[6,7-b]indole heterocyclic hybrids: Stereoselective Synthesis, Cholinesterase Inhibitory Activity and Their Molecular Docking Study. Bioorg. Chem..

[28] Yavari I., Khajeh-Khezri A.K. (2018). Recent Advances in the Synthesis of Hetero- and Carbocyclic Compounds and Complexes Based on Acenaphthylene-1,2-dione. Synthesis.

[29] Babu S., Raghunathan R., Mathivanan N., Omprabha G., Velmurugan D., Raghu R. (2008). Synthesis, Characterisation and Anti-Microbial Activity Studies of Novel Dispiro-Oxindolopyrrolizidines. Curr. Chem. Biol..

[30] Gollner A., Weinstabl H., Fuchs J.E., Rudolph D., Garavel G., Hofbauer K.S., Karolyi-Oezguer J., Gmaschitz G., Hela W., Kerres N. (2019). Targeted Synthesis of Complex Spiro[3H-indole-3,2´-pyrrolidin]-2(1H)-ones by a Novel Intramolecular Cyclization of Azomethine Ylides as Highly Potent MDM2–p53 Inhibitors. Chem. Med. Chem..

[31] Saraswat P., Jeyabalan G., Hassan M., Rahman M.U., Nyola N.K. (2016). Review of synthesis and various biological activities of spiro heterocyclic compounds comprising oxindole and pyrrolidine moieties. Synth. Commun..

[32] Jesudoss H.M., Saminathan M., Jesudoss R.E., Murugan D., Alagusundaram P. (2018). Crystal Structure, Spectral, Electronic, NLO Studies, and Bioactivity of 3′-(1-Benzyl-5-Methyl-1H-1,2,3-Triazole-4-Carbonyl)-4′-(4-Bromophenyl)-1′-Methyl-2H-Spiro [Acenaphthylene-1,2′-Pyrrolidine]-2-One. Braz. J. Phys..

[33] Malarkodi J.H., Murugavel S., Ezhilarasi J.R., Dinesh M., Ponnuswamy A. (2019). Structure Investigation, Spectral Characterization, Electronic Properties, and Antimicrobial and Molecular Docking Studies of 3’-(1-benzyl-5-methyl-1H-1,2,3-triazole-4-carbonyl)-1’-methyl4’-phenyl-2H-spiro[acenaphthylene-1,2’-pyrrolidine]-2-one. J. Chin. Chem. Soc..

[34] Hassan M.Z., Ali M.A., Osman H., Kumar R.S., Arumugam N. (2016). Design, Synthesis and Antimycobacterial Activity of Dispiropyrrolidines Derivatives. Med. Chem..

[35] Qian Y.-L., Li B., Xia P.-J., Wang J., Xiang H.-Y., Yang H. (2018). Diastereospecific Entry to Pyrrolidinyldispirooxindole Skeletons *via three*-Component 1,3-Dipolar Cycloadditions. Tetrahedron.

[36] Kumar R.S., Almansour A.I., Arumugam N., Periyasami G., Athimoolam S., Kumar R.R., Asad M., Asiri A.M. (2018). Dipolar Cycloaddition Based Multi-Component Reaction: Synthesis of Spiro Tethered Acenaphthylene–Indolizine–Pyridinone Hybrids. Tetrahedron Lett..

[37] Sumesh R.V., Shylaja A., Kumar R.R., Almansour A.I., Kumar R.S. (2018). Synthesis of Spiro-Linked Quinolinone-Pyrrolidine/Pyrrolo[1,2-c] Thiazole-Oxindole/Acenaphthalene Hybrids via Multi-component [3 + 2] Cycloaddition. Tetrahedron Lett..

[38] Rani G.U., Kumar S.V., Bharkavi C., Menéndez J.C., Perumal S. (2016). One-pot Access to a Library of Dispiro Oxindole-Pyrrolidine/Pyrrolothiazolethiochromane Hybrids *via* Three-component 1,3-Dipolar Cycloaddition Reactions. ACS Comb. Sci..

[39] Rani M.A., Kumar S.V., Malathi K., Muthu M., Almansour A.I., Kumar R.S., Kumar R.R. (2017). A One-Pot Multicomponent 1,3-Dipolar Cycloaddition Strategy: Combinatorial Synthesis of Dihydrothiophenone-Engrafted Dispiro Hybrid Heterocycles. ACS Comb. Sci..

[40] Arumugam N., Almansour A.I., Kumar R.S., Periasamy V.S., Athinarayanan J., Alshatwi A.A., Govindasami P., Altaf M., Menéndez J.C. (2018). Regio- and Diastereoselective Synthesis of Anticancer Spirooxindoles Derived from Tryptophan and Histidine via Three-Component 1,3-Dipolar Cycloadditions in an Ionic Liquid. Tetrahedron.

[41] Tiwari K.N., Pandurang T.P., Pant S., Sreelekha P. (2018). Efficient Synthesis of Spirooxindole-Pyrrolizidines and Dispirooxindole-Piperazines. Synth. Commun..

[42] Ishiyama T., Tokuda K., Ishibashi T., Ito A., Toma S., Ohno Y. (2007). Lurasidone (SM-13496), A Novel Atypical Antipsychotic Drug, Reverses MK-801-Induced Impairment of Learning and Memory in The Rat Passive-Avoidance Test. Eur. J. Pharmacol..

[43] Patil M.M., Rajput S.S. (2014). Succinimides: Synthesis, Reaction and Biological Activity. Int. J. Pharm. Sci..

[44] Isaka M., Rugseree N., Maithip P., Kongsaeree P., Prabpai S., Thebtaranonth Y. (2005). Hirsutellones A—E, Antimycobacterial Alkaloids from the Insect Pathogenic Fungus Hirsutella nivea BCC 2594. Tetrahedron.

[45] Matviiuk T., Morid G., Lherbeta C., Rodriguez F., Pasca M.R., Gorichko M., Guidettia B., Voitenko Z., Baltasa M. (2014). Synthesis of 3-Heteryl Substituted Pyrrolidine-2,5-diones *via* Catalytic Michael Reaction and Evaluation of Their Inhibitory Activity Against InhA and Mycobacterium Tuberculosis. Eur. J. Med. Chem..

[46] Zhao S., Li H., Chang X., Wang J., Zhao E., Yin Z., Mao X., Deng S., Hao T., Wang H. (2019). Synthesis, in vitro Stability, and Antiproliferative Effect of D-cysteine Modified GnRH-doxorubicin Conjugates. J. Pept. Sci..

[47] Socala K., Mogilski S., Pieróg M., Nieoczym D., Abram M., Szulczyk B., Lubelska A., Latacz G., Doboszewska U., Wla P. (2019). KA-11, A Novel Pyrrolidine-2,5-dione Derived Broad-Spectrum Anticonvulsant: Its Antiepileptogenic, Antinociceptive Properties and *in vitro* Characterization. ACS Chem. Neurosci..

[48] Rapacz A., Obniska J., Wiklik-Poudel B., Rybka S., Sałat K., Filipek B. (2016). Anticonvulsant and antinociceptive activity of new amides derived from 3-phenyl-2,5-dioxo-pyrrolidine-1-yl-acetic acid in mice. Eur. J. Pharmacol..

[49] Boudriga S., Elmhawech B., Askri M. (2019). Straightforward and Highly Diastereoselective Synthesis of a New Set of Functionalized Dispiropyrrolidines Involving Multicomponent 1,3-Dipolar Cycloaddition with Azomethine Ylides. J. Het. Chem..

[50] Boudriga S., Haddad S., Askri M., Soldera A., Knorr M., Strohmann C., Golz C. (2019). Highly Diastereoselective Donstruction of Noveldispiropyrrolo[2,1-a]isoquinoline Derivatives *via* Multicomponent 1,3-Dipolar Dycloaddition of Dyclic Diketones-Based Tetrahydroisoquinolinium *N-*Ylides. RSC Adv..

[51] Haddad S., Boudriga S., Akhaja T.N., Raval J.P., Porzio F., Soldera A., Askri M., Knorr M., Rousselin Y., Kubicki M.M. (2015). A Strategic Approach to the Synthesis of Functionalized Spirooxindole Pyrrolidine Derivatives: In vitro Antibacterial, Antifungal, Antimalarial and Antitubercular studies. New J. Chem..

[52] Haddad S., Boudriga S., Parzo F., Soldera A., Askri M., Knorr M., Rousselin Y., Kubicki M.M., Golz C., Strohmann C. (2015). Regio- and Stereoselective Synthesis of Spiropyrrolizidines and Piperazines through Azomethine Ylide Cycloaddition Reaction. J. Org. Chem..

[53] Mhiri C., Boudriga S., Askri M., Knorr M., Sriram D., Yogeeswari P., Nana F., Golz C., Strohmanni C. (2015). Design of Novel Dispirooxindolopyrrolidine and Dispirooxindolopyrrolothiazole Derivatives as Potential Antitubercular Agents. Bioorg. Med. Chem. Lett..

[54] Houk K.N. (1970). The Role of Secondary Orbital Interactions in Cycloaddition Reactions. Tetrahedron Lett..

[55] Maurya R.A., Nayak R., Reddy C.N., Kapure J.S., Nanubolu J.B., Singarapu K.K., Ajitha M., Kamal A. (2014). Regio- and Stereoselective Synthesis of Novel Spiropyrrolidines through 1,3-dipolar Cycloaddition Reactions of Azomethine Ylides and 2-Styrylquinazolin-4(3H)-ones. RSC Adv..

[56] Rosenberry T.L. (1975). Synthesis of Novel Spirooxindolo-Pyrrolidines, Pyrrolizidines, and Pyrrolothiazoles *via* a Regioselective three-Component [3+2] Cycloaddition and their Preliminary Antimicrobial Evaluation. Adv. Enzymol. Relat. Areas Mol. Biol..

[57] Marco L., Carreiras M. (2006). Galanthamine, a Natural Product for the Treatment of Alzheimer’s Disease. Recent Pat. CNS Drug Discov..

[58] Xu Y., Colletier J.-P., Weik M., Jiang H., Moult J., Silman I., Sussman J.L. (2008). Flexibility of Aromatic Residues in the Active-Site Gorge of Acetylcholinesterase: X-ray versus Molecular Dynamics. Biophysical J..

[59] Dolomanov O.V., Bourhis L.J., Gildea R.J., Howardand J.A.K., Puschmann H. (2009). OLEX2: A Complete Structure Solution, Refinement and Analysis Program. J. Appl. Crystallogr..

[60] Sheldrick G.M. (2008). A short history of SHELX. Acta Crystallogr..

[61] Morris G.M., Goodsell D.S., Halliday R.S., Huey R., Hart W.E., Belew R.K., Olson A.J. (1998). Automated Docking Using a Lamarckian Genetic Algorithm and An Empirical Binding Free Energy Function. J. Comput. Chem..

[62] Greenblatt H.M., Guillou C., Guenard D., Argaman A., Botti S., Badet B., Thal C., Silman I., Sussman J.L. (2004). The Complex of a Bivalent Derivative of Galanthamine with Torpedo Acetylcholinesterase Displays Drastic Deformation of the Active-Site Gorge: Implications for Structure-Based Drug Design. J. Am. Chem. Soc..

