# Supplementary material for: Three-Component Access to Functionalized Spiropyrrolidine Heterocyclic Scaffolds and Their Cholinesterase Inhibitory Activity
Source: Molecules. 2020 Apr 23;25(8):1963. doi: 10.3390/molecules25081963 (PMC7221748; doi:10.3390/molecules25081963)
Supplement: Supplementary file 1 [file molecules-25-01963-s001.pdf]

molecules

Supplementary Materials

Three-component Access to Functionalized Spiropyrrolidine Heterocyclic Scaffolds and Their Cholinesterase Inhibitory Activity.

Sarra Boudriga [1,*], Saoussen Haddad [1], Vikneswaran Murugaiyah [2], Moheddine Askri [1,*], Michael Knorr [3,*], Carsten Strohmann [4] and Christopher Golz [4]

[1] Department of Chemistry, Laboratory of Heterocyclic Chemistry Natural Product and Reactivity/CHPNR, Faculty of Science of Monastir, 5000 Monastir, Tunisia; haddad_saoussen@live.fr

[2] Discipline of Pharmacology, School of Pharmaceutical Sciences, Universiti Sains Malaysia, 11800 USM, Penang, Malaysia; vicky@usm.my

[3] Institut UTINAM - UMR CNRS 6213, Université Bourgogne Franche-Comté, 16 Route de Gray, 25030 Besançon, France°

[4] Technische Universität Dortmund, Anorganische Chemie Otto-Hahn-Straße 6,44221 Dortmund, Germany; carsten.strohmann@tu-dortmund.de (C.S.); christopher.golz@tu-dortmund.de (C.G.).

* Correspondence: sarra_boudriga@yahoo.fr **(S.B.)**; moheddine.askri@fsm.rnu.tn (M.A.); michael.knorr@univ-fcomte.fr (M.K.); Tel.: +216-21-14-76-07 (S.B); Tel.: +216-98-67-61-87 (M.A.); Tel.:+33-3-8166-6270 (M.K.)

Table of Contents

		Pages
1	^{1}H- and ^{13}C-NMR Spectra of compounds 4a-r and 5a-r (Fig. S1 to S48)	2–25
2	^{1}H- and ^{13}C-NMR Spectra of compounds 7a-h (Fig. S49 to S64)	26–33
3	Cholinesterase inhibitory assay	34
4	Molecular Docking of compound **4n**	34

1. ^{1}H- and ^{13}C-NMR Spectra of compounds 4a-r and 5a-r (Fig. S1 to S36)

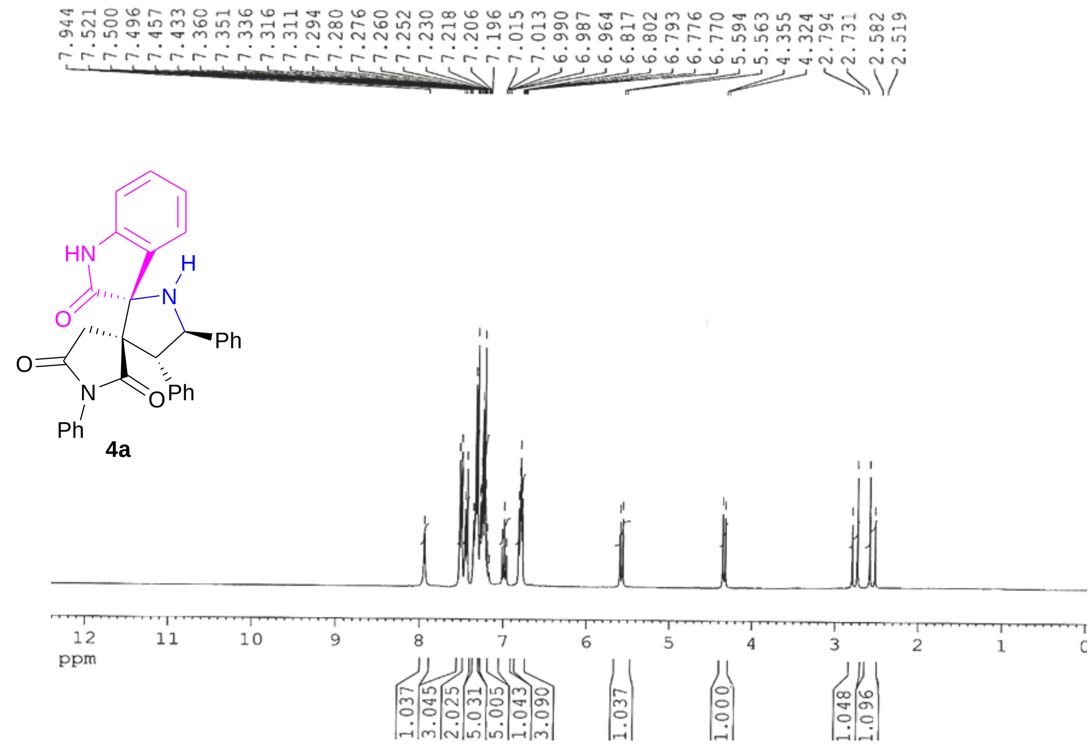

Fig. S1. ^{1}H NMR spectrum of **4a** in $CDCl_3$

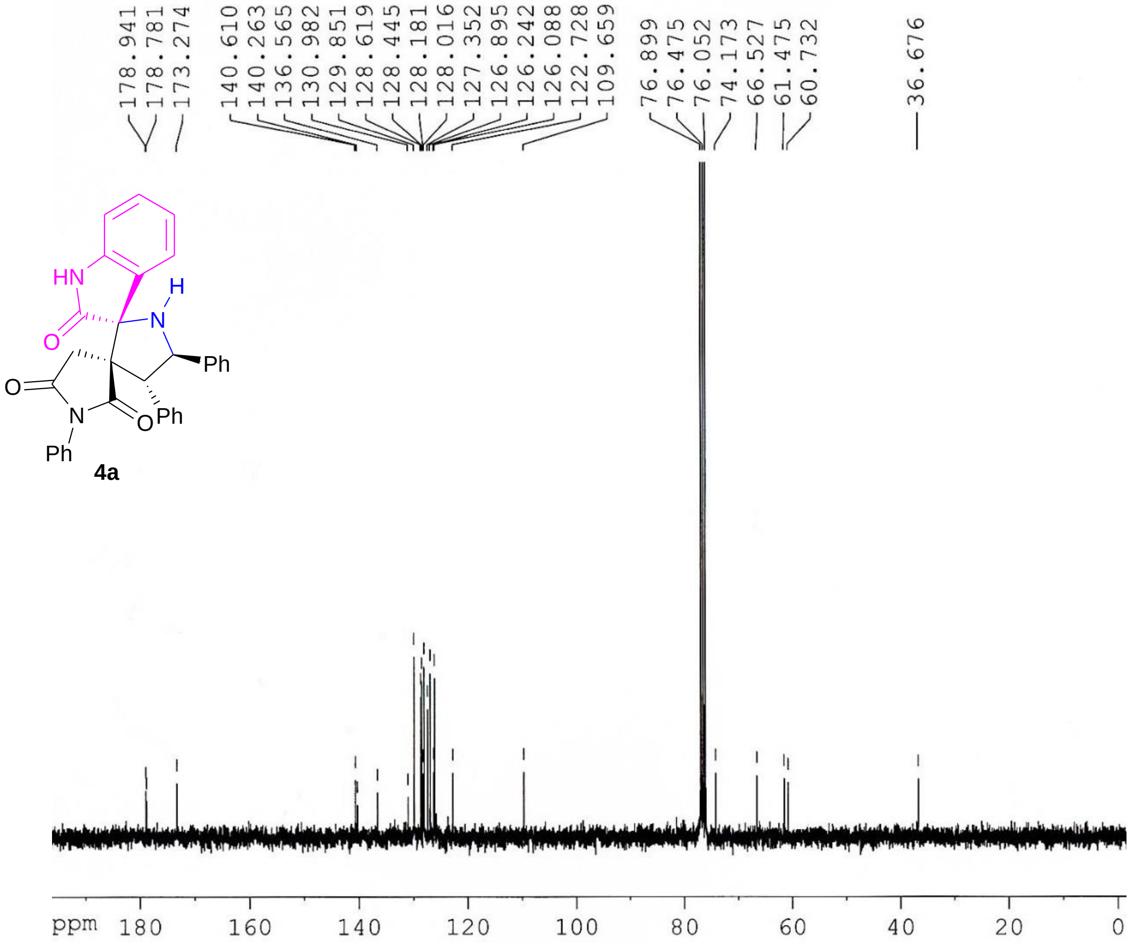

Fig. S2. ^{13}C NMR spectrum of **4a** in $CDCl_3$

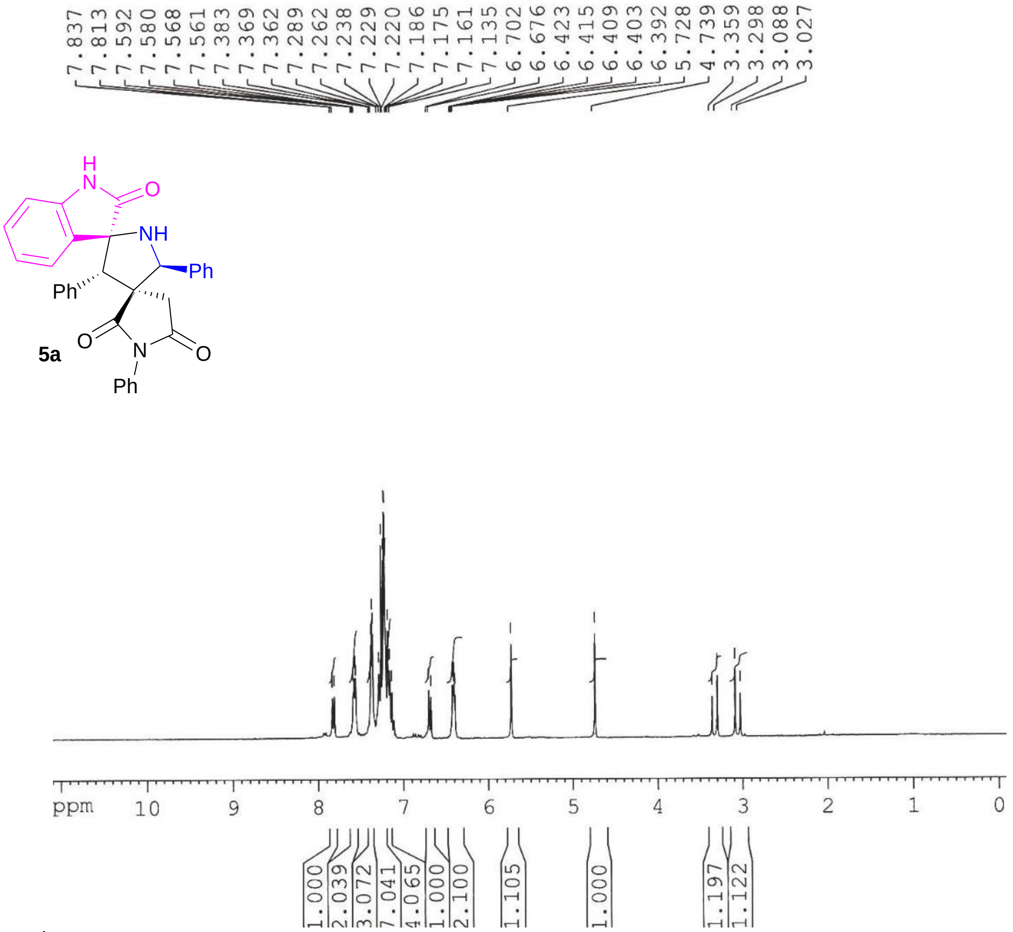

Fig. S3.^{1}H NMR spectrum of **5a** in $CDCl_3$

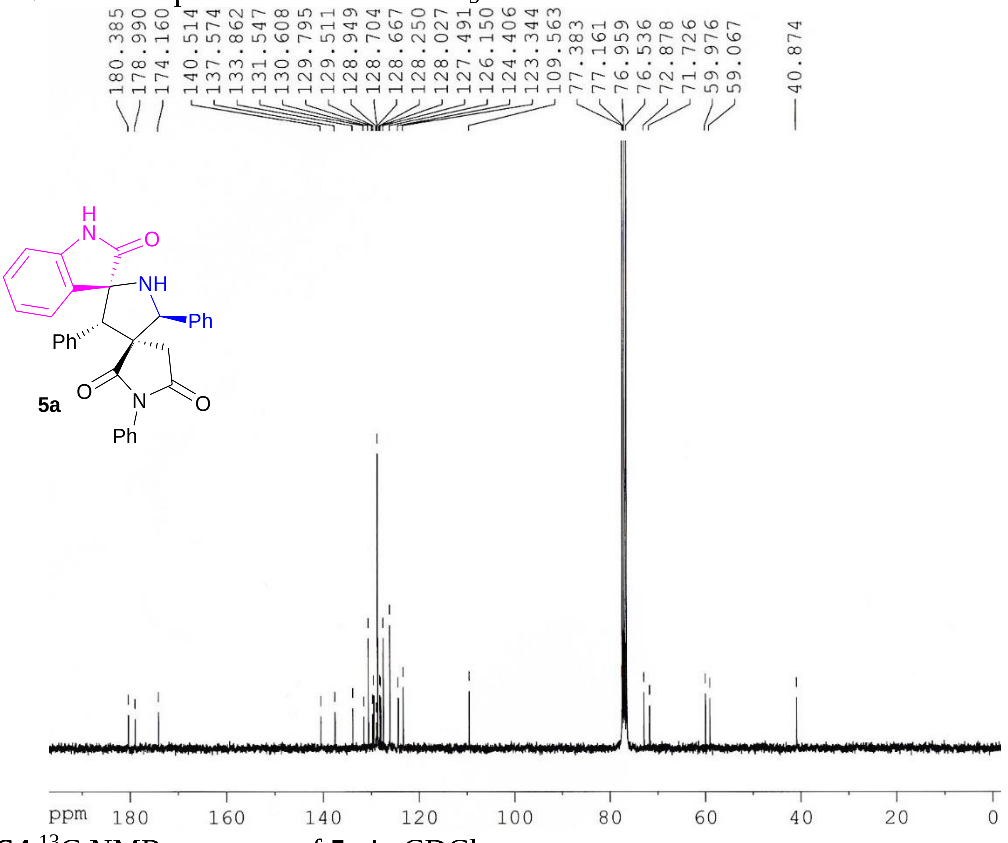

Fig. S4.^{13}C NMR spectrum of **5a** in $CDCl_3$

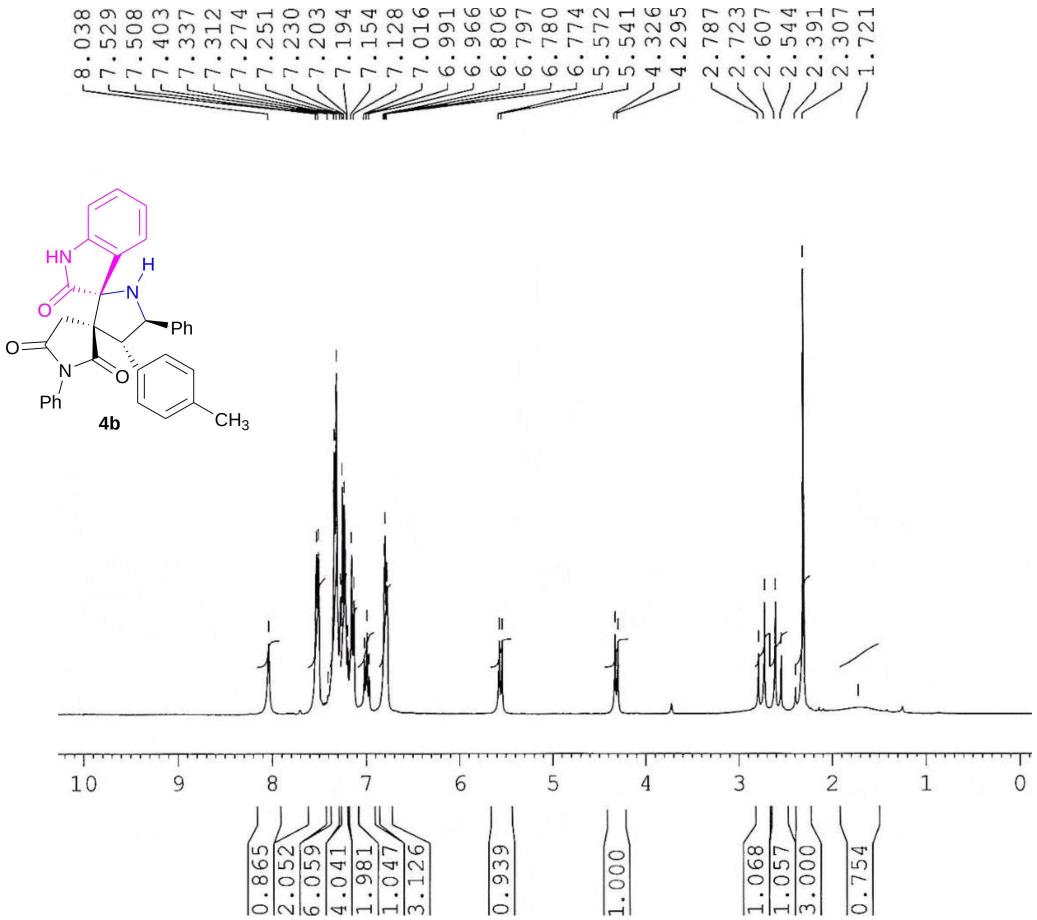

Fig. S5.^{1}H NMR spectrum of **4b** in $CDCl_3$

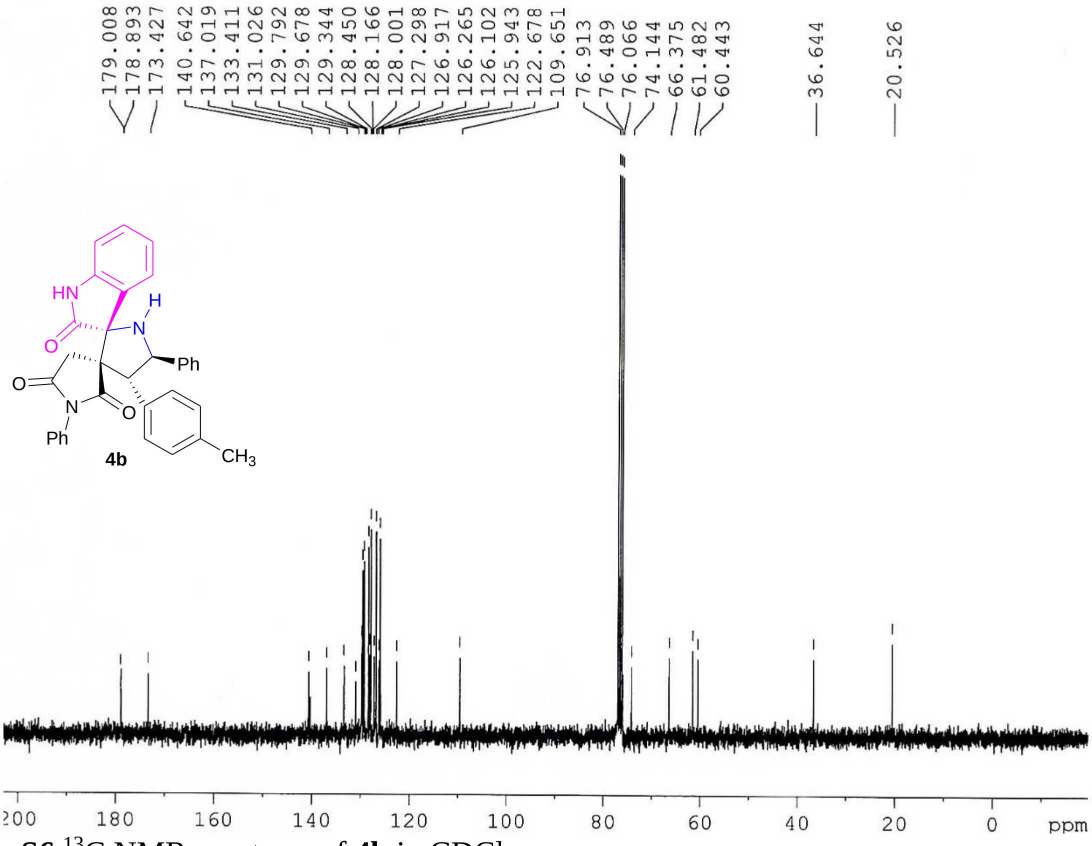

Fig. S6.^{13}C NMR spectrum of **4b** in $CDCl_3$

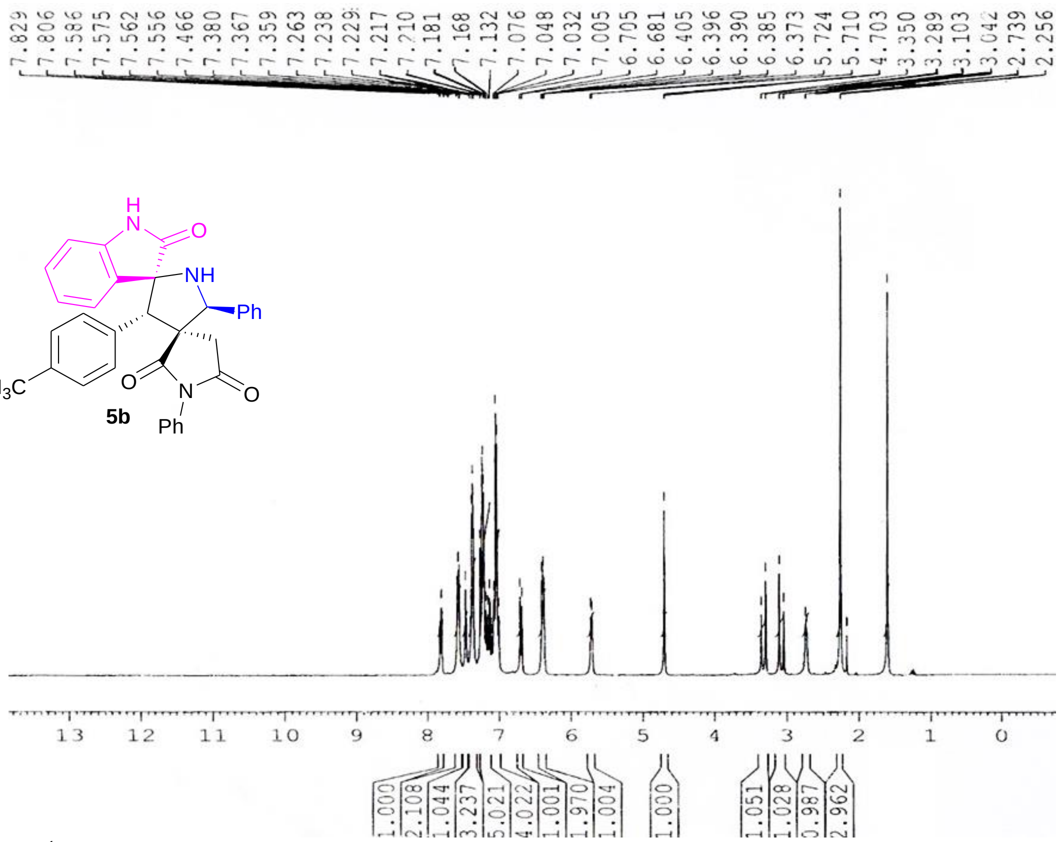

Fig. S7.^{1}H NMR spectrum of **5b** in $CDCl_3$

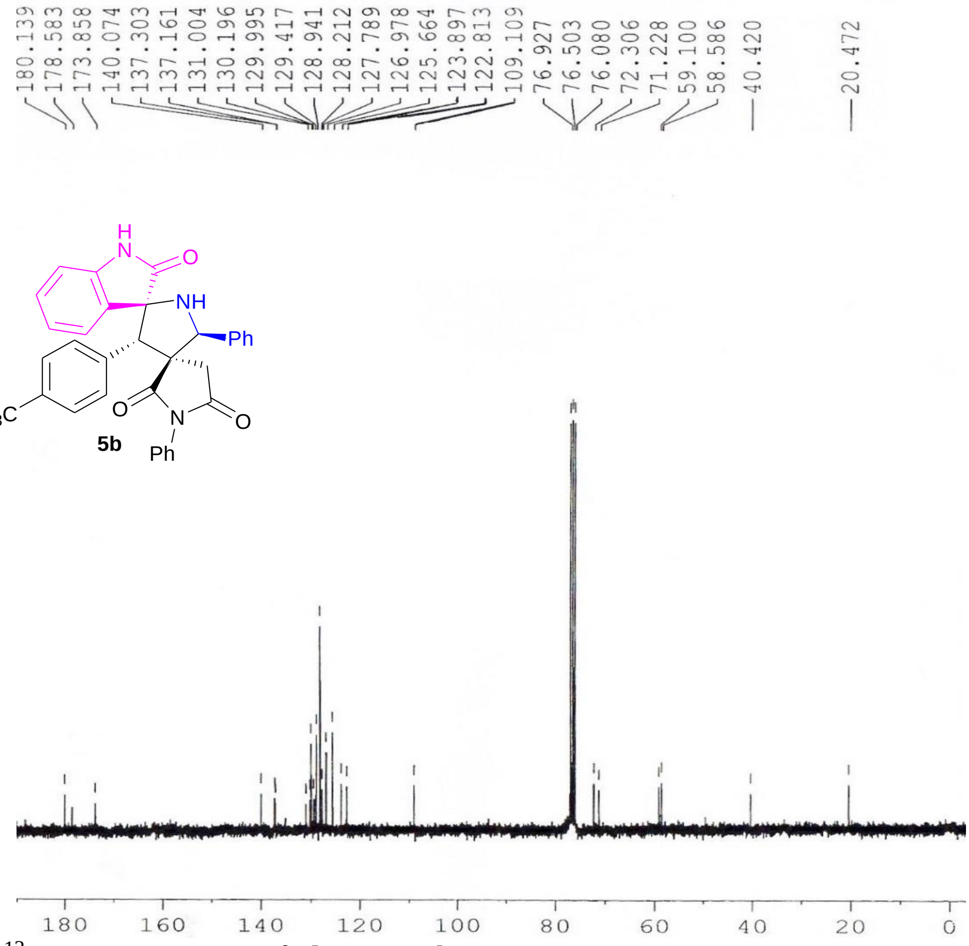

Fig. S8.^{13}C NMR spectrum of **5b** in $CDCl_3$

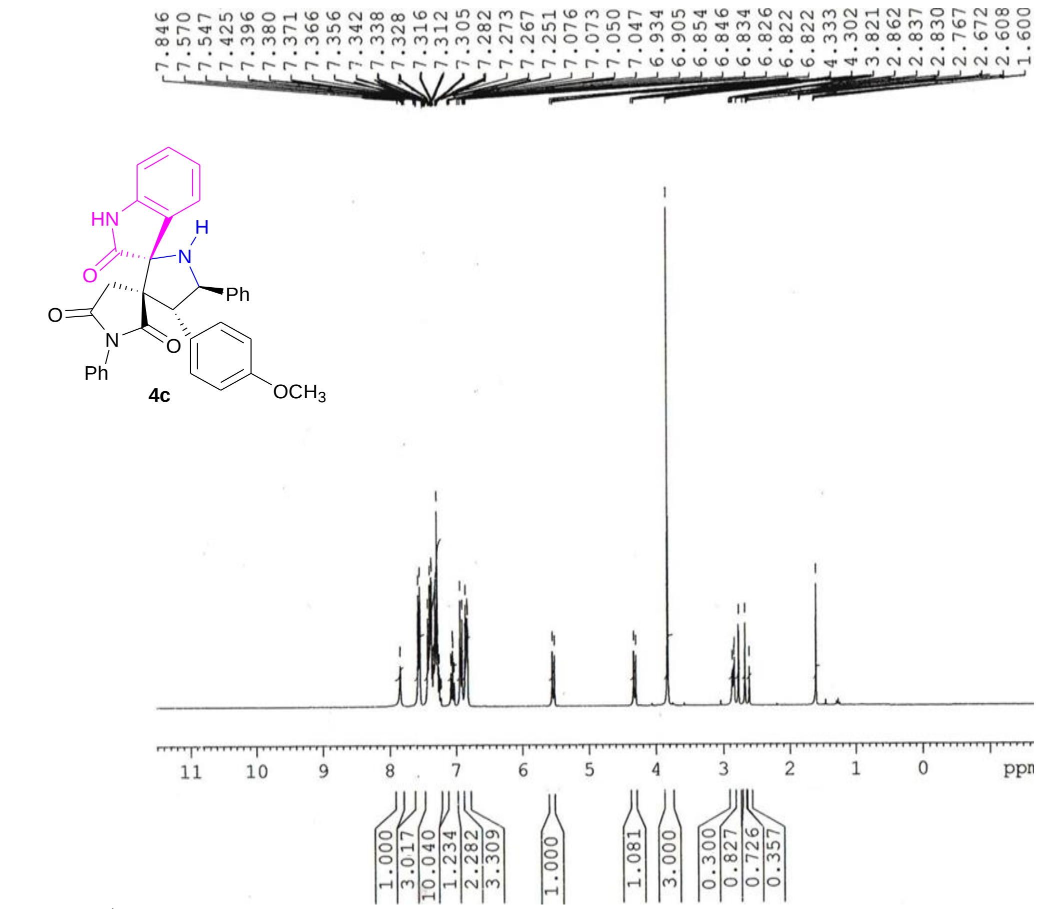

Fig. S9. ^{1}H NMR spectrum of **4c** in $CDCl_3$

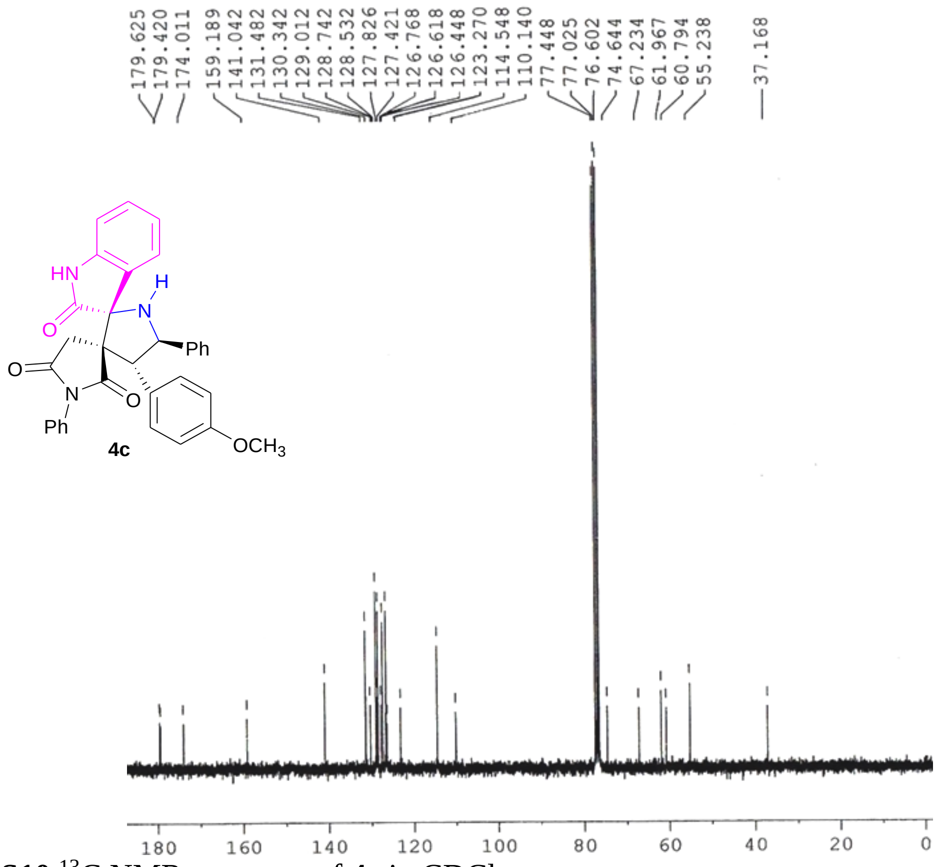

Fig. S10. ^{13}C NMR spectrum of **4c** in $CDCl_3$

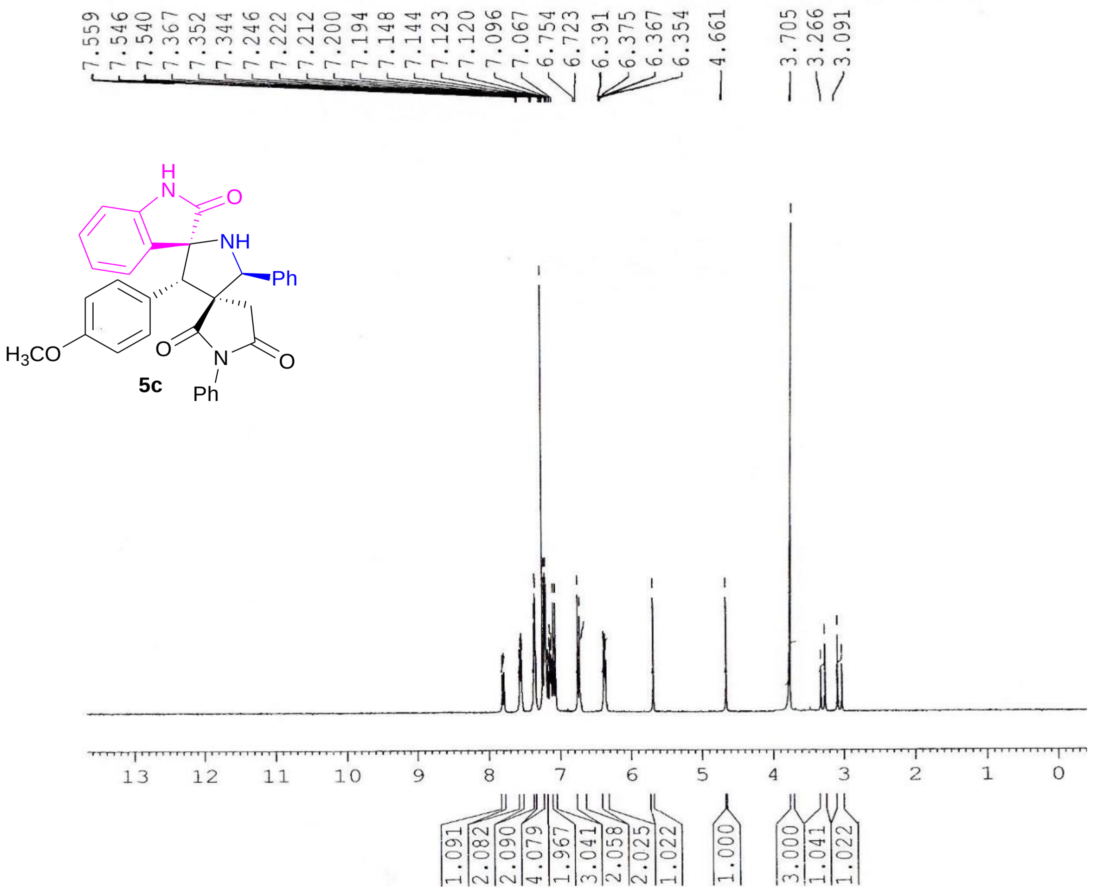

Fig. S11. ^{1}H NMR spectrum of **5c** in $CDCl_3$

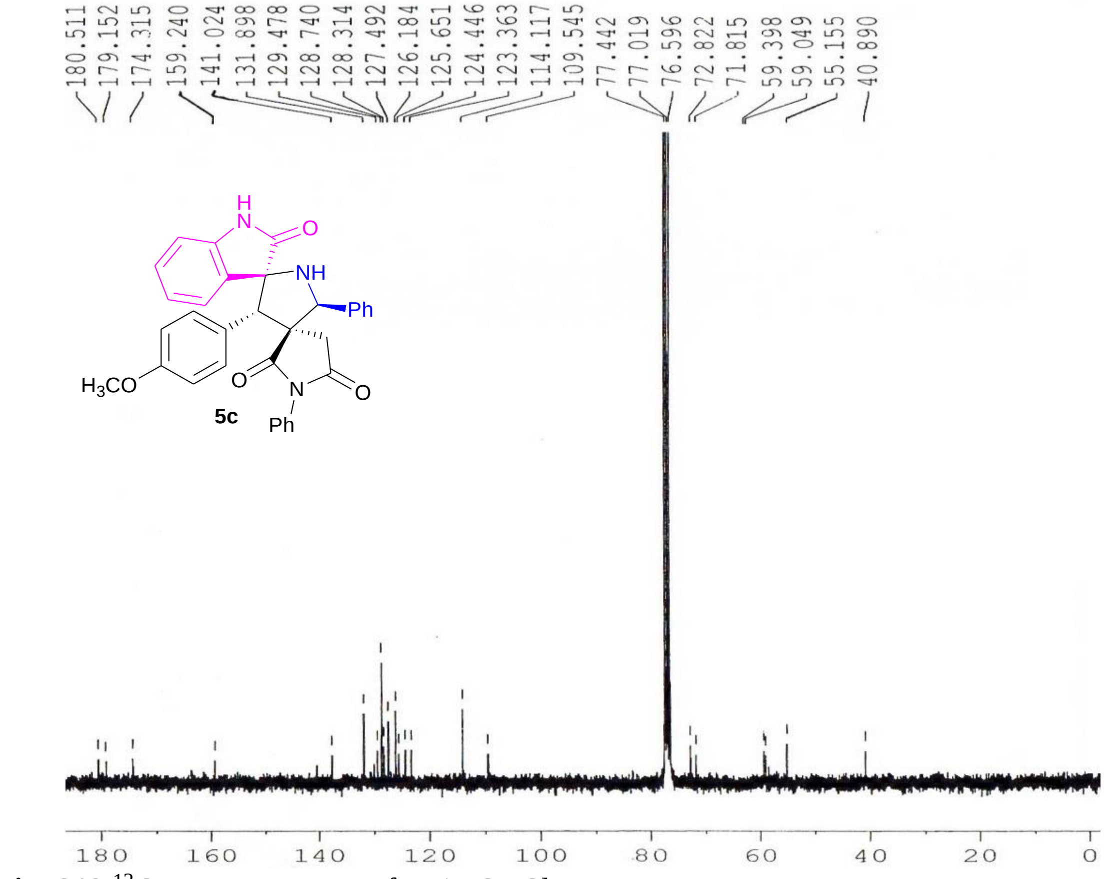

Fig. S12. ^{13}C NMR spectrum of **5c** in $CDCl_3$

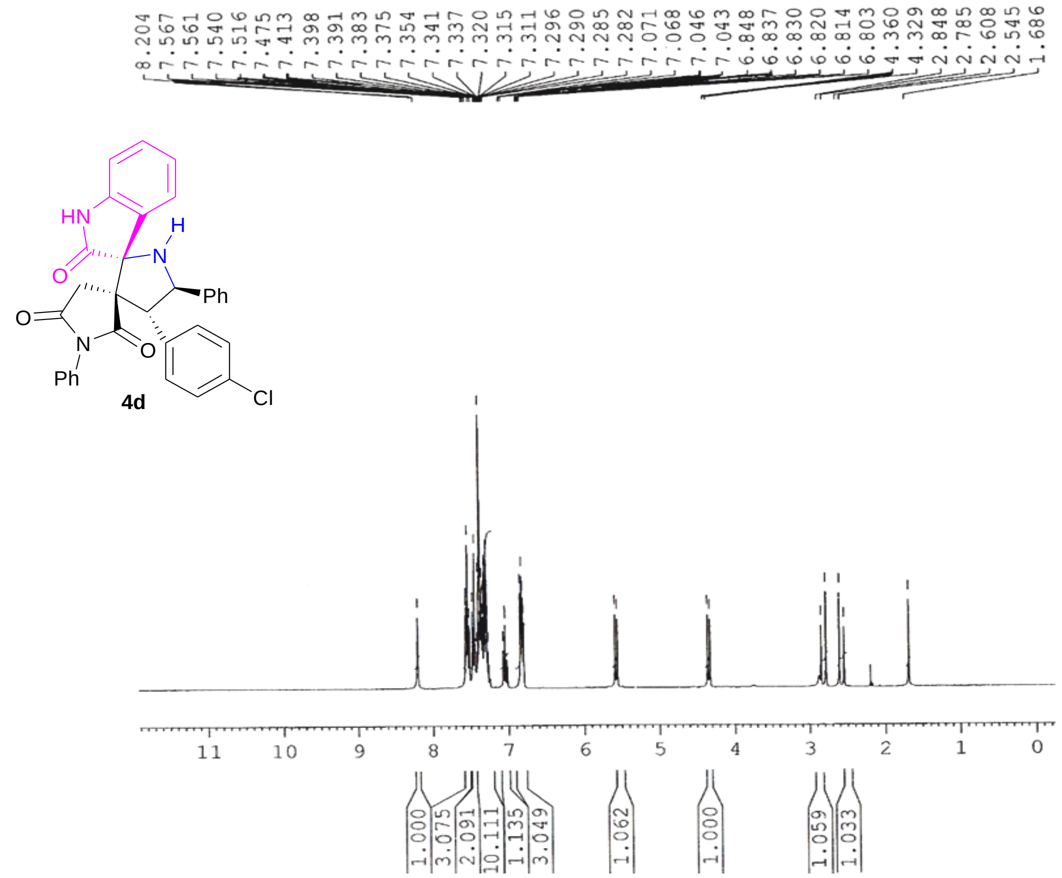

Fig. S13.^{1}H NMR spectrum of **4d** in $CDCl_3$

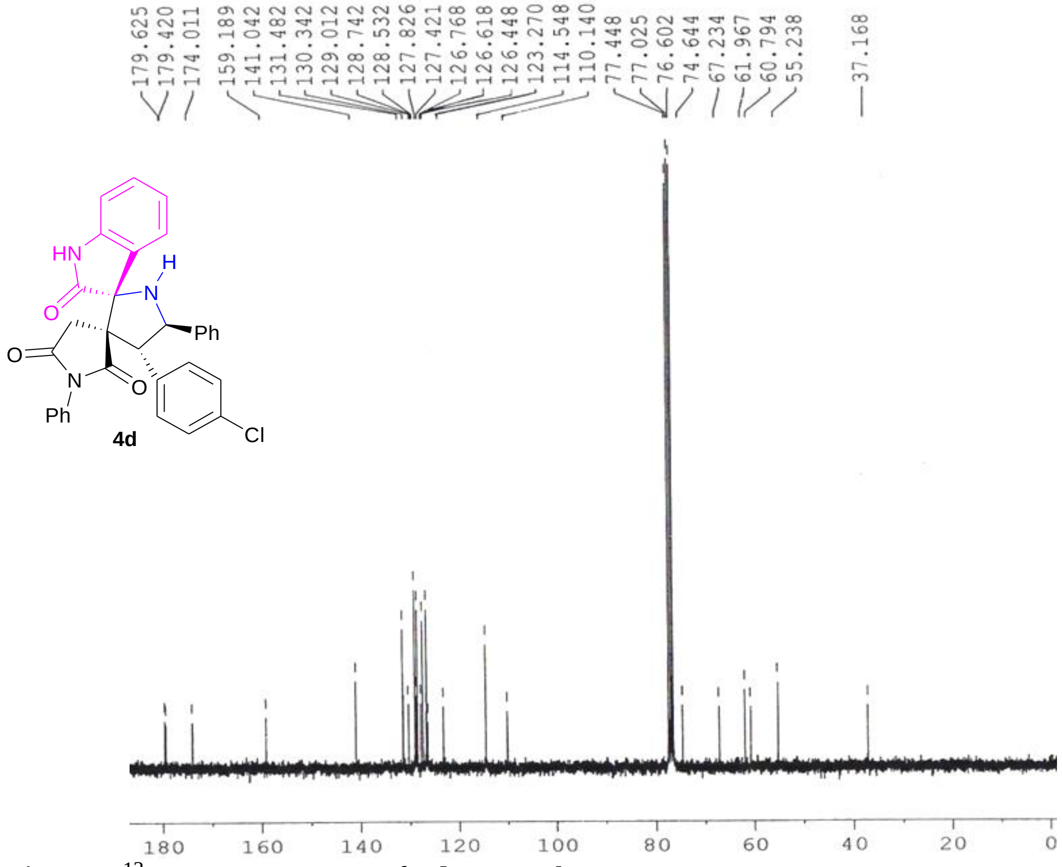

Fig. S14.^{13}C NMR spectrum of **4d** in $CDCl_3$

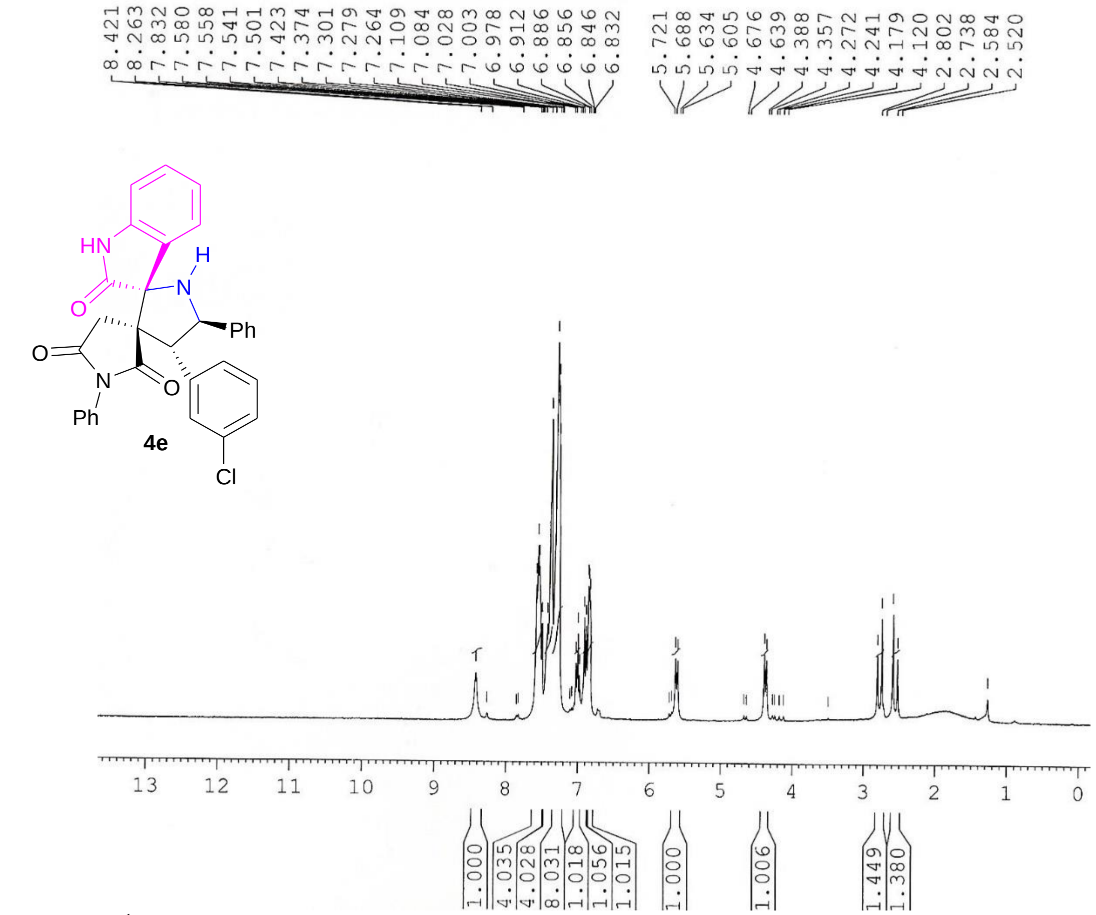

Fig. S15. ^{1}H NMR spectrum of **4e** in $CDCl_3$

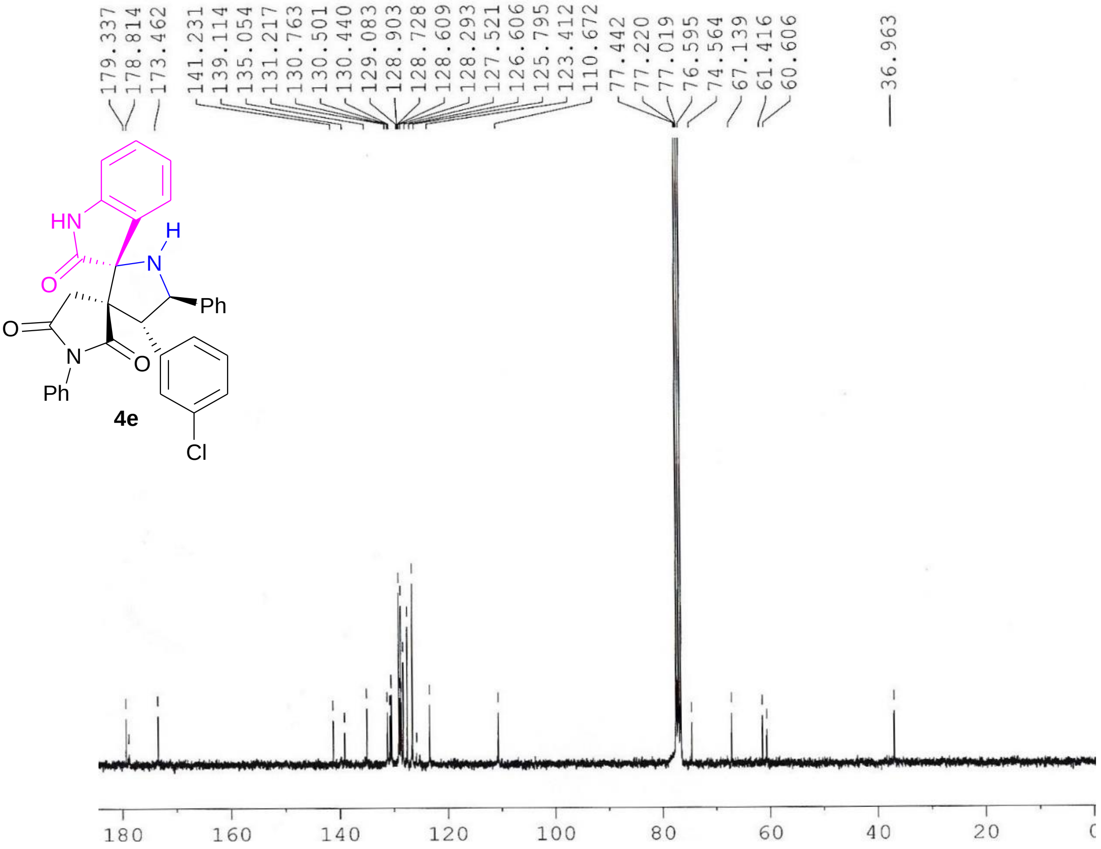

Fig. S16. ^{13}C NMR spectrum of **4e** in $CDCl_3$

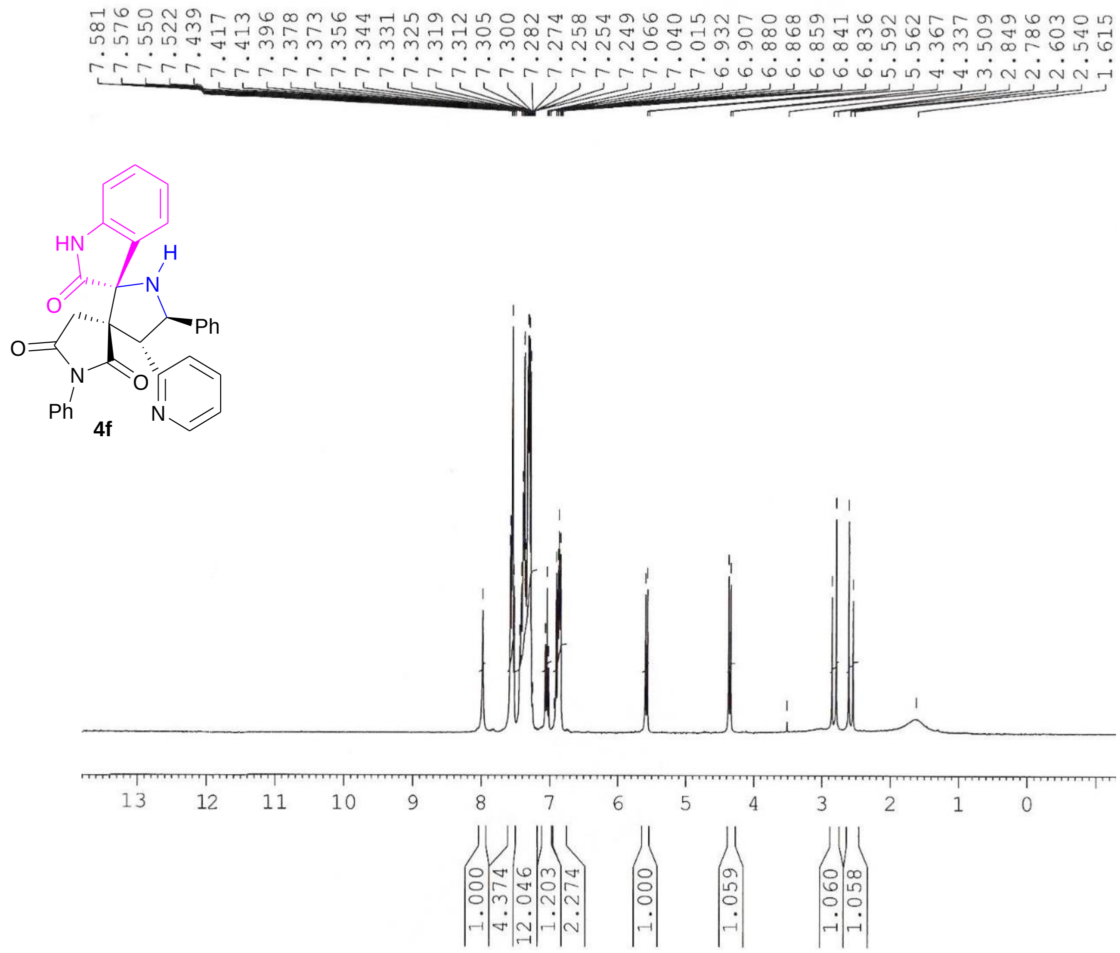

Fig. S17.^{1}H NMR spectrum of **4f** in $CDCl_3$

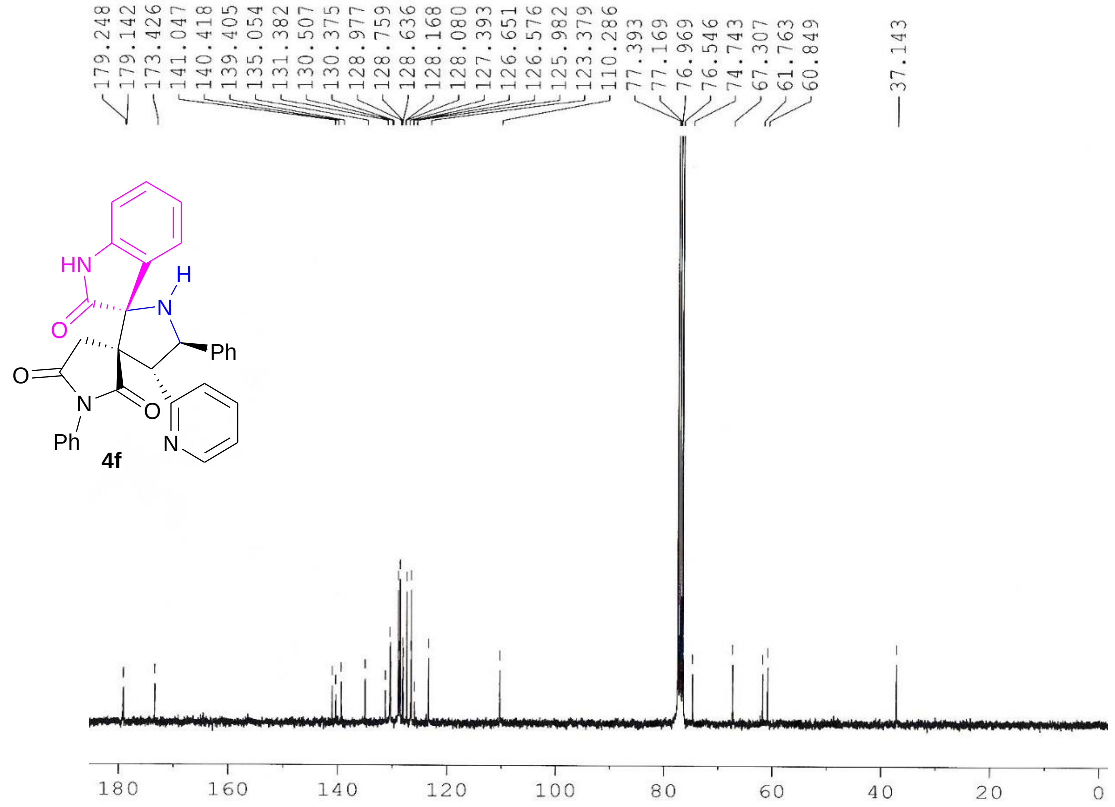

Fig. S18.^{13}C NMR spectrum of **4f** in $CDCl_3$

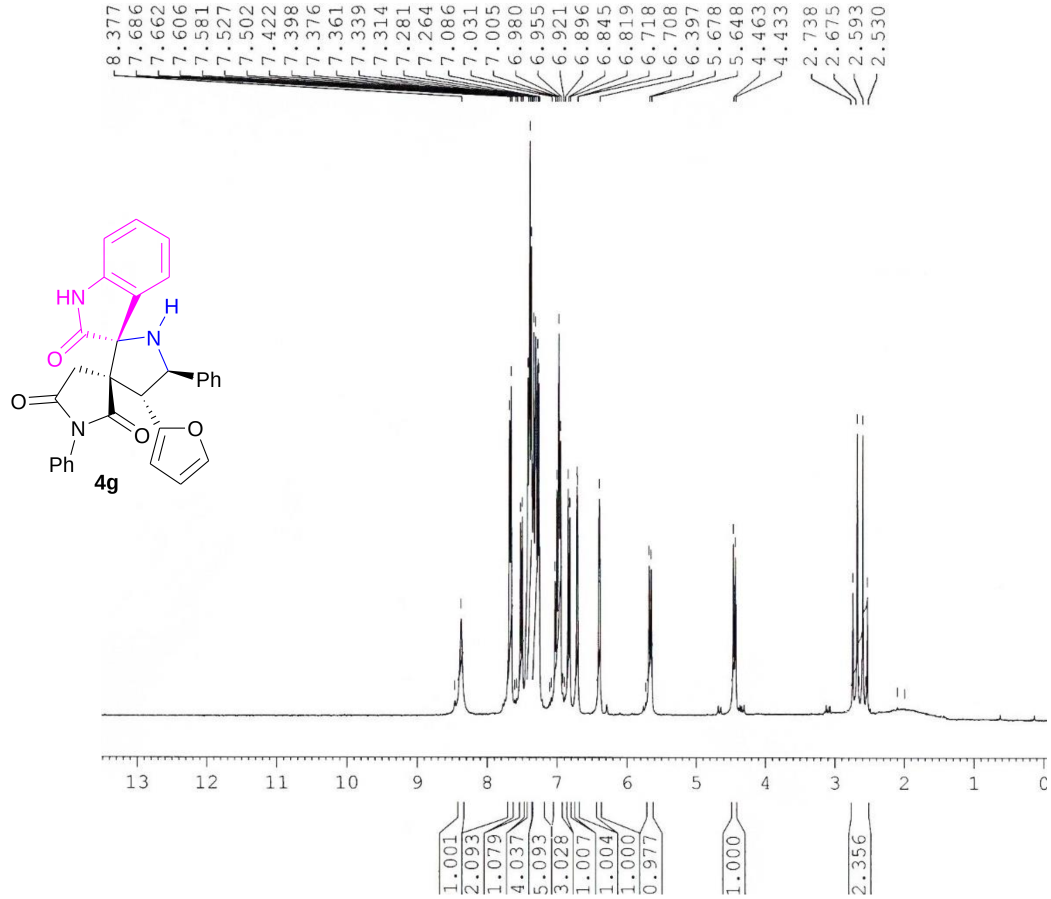

Fig. S19.^{1}H NMR spectrum of **4g** in $CDCl_3$

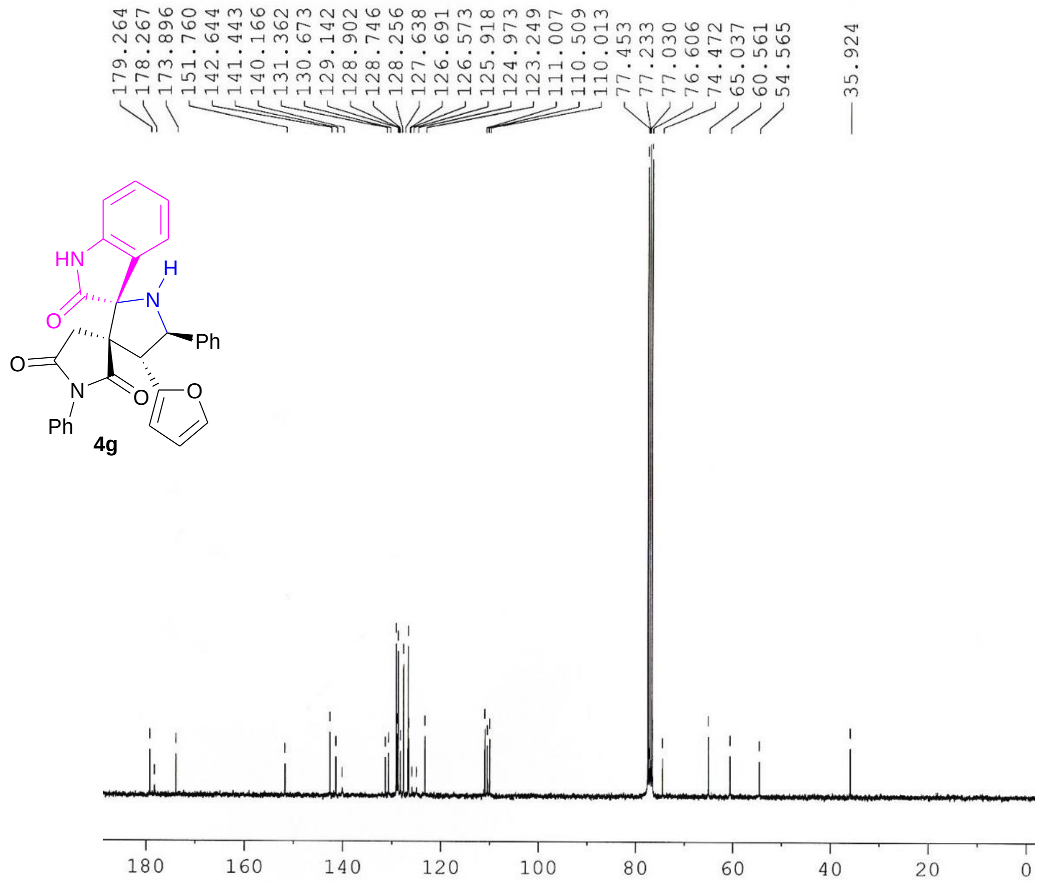

Fig. S20.^{13}C NMR spectrum of **4g** in $CDCl_3$

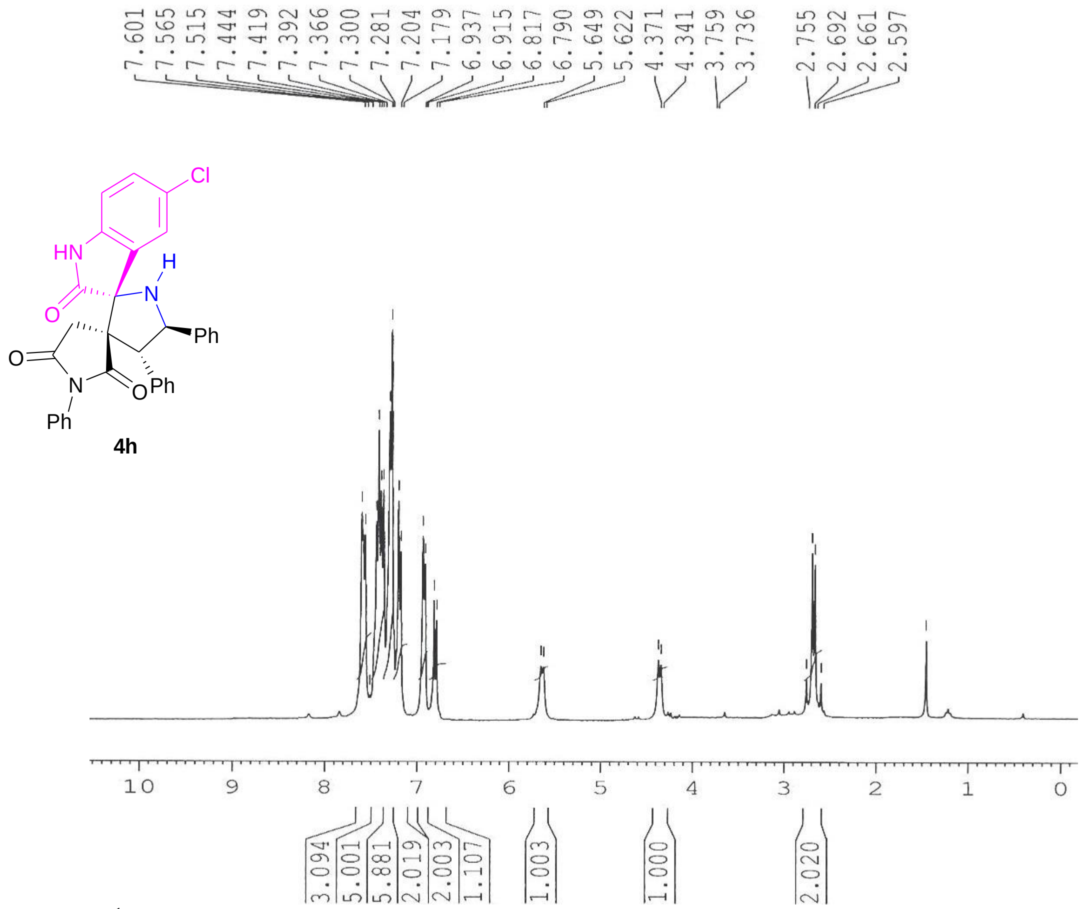

Fig. S21.^{1}H NMR spectrum of **4h** in $CDCl_3$

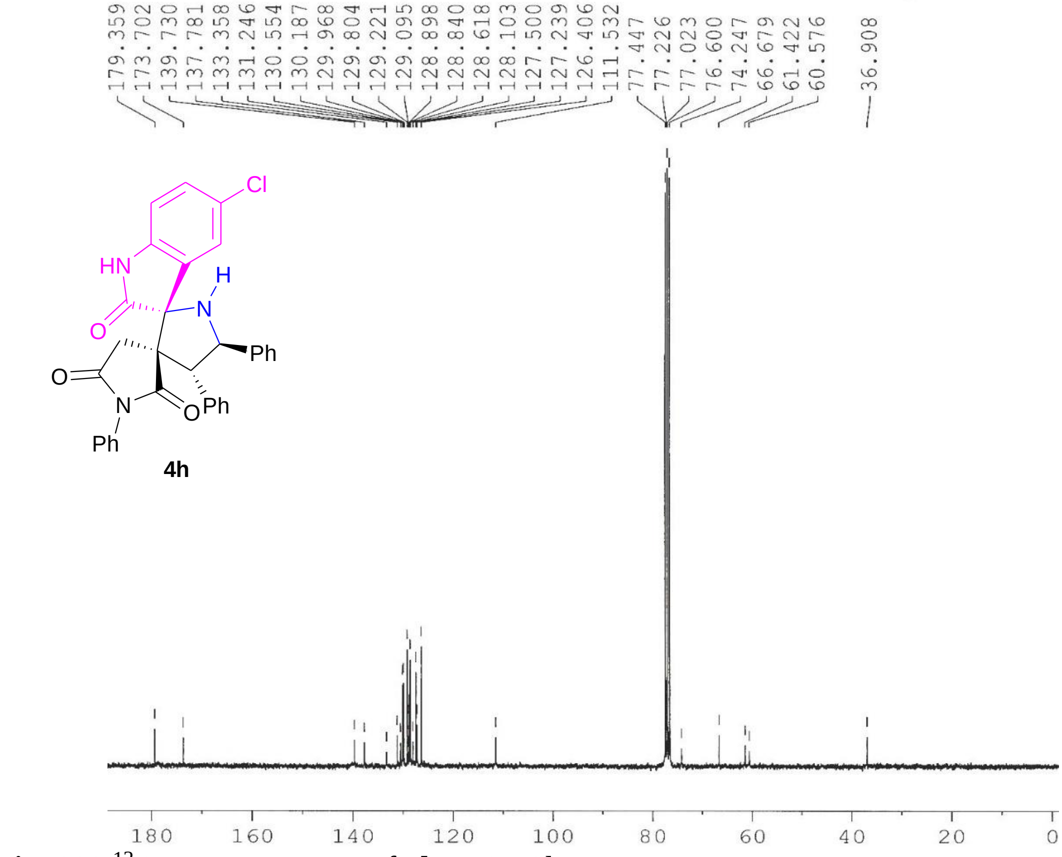

Fig. S22.^{13}C NMR spectrum of **4h** in $CDCl_3$

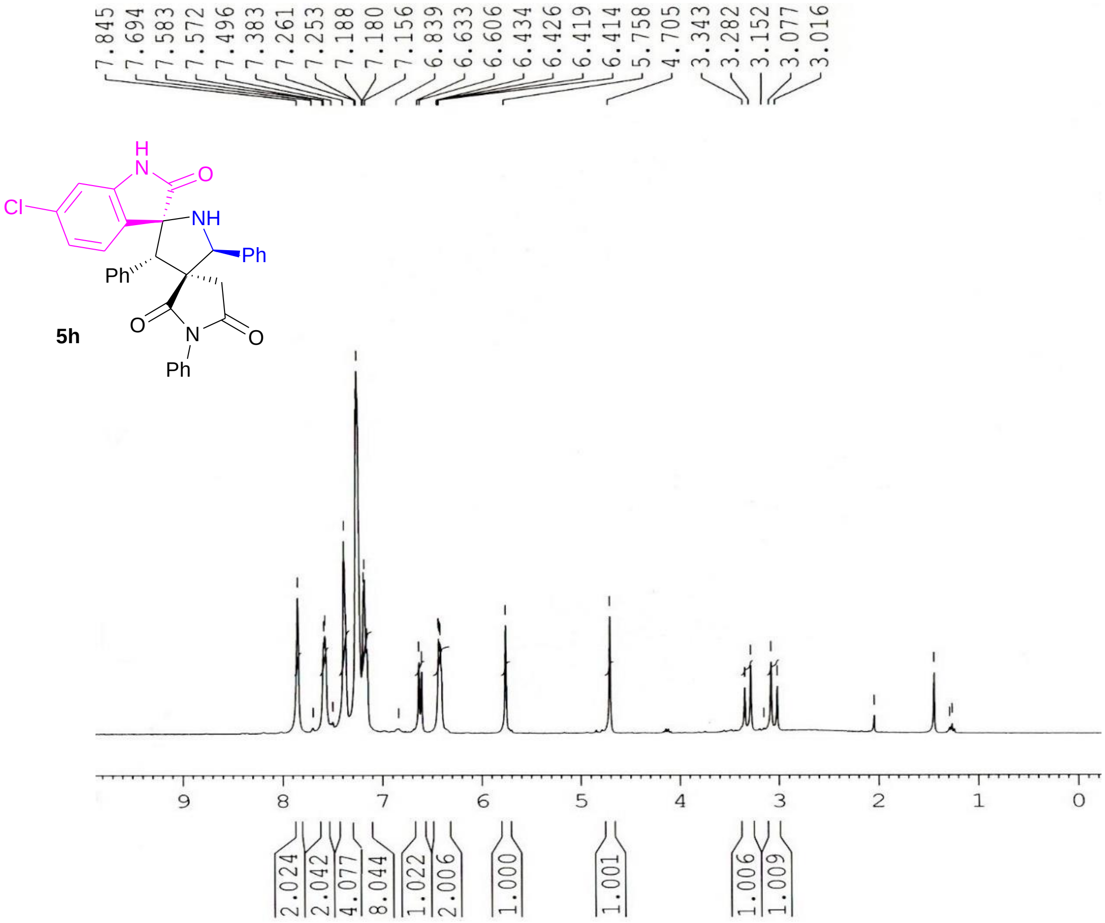

Fig. S23.^{1}H NMR spectrum of **5h** in $CDCl_3$

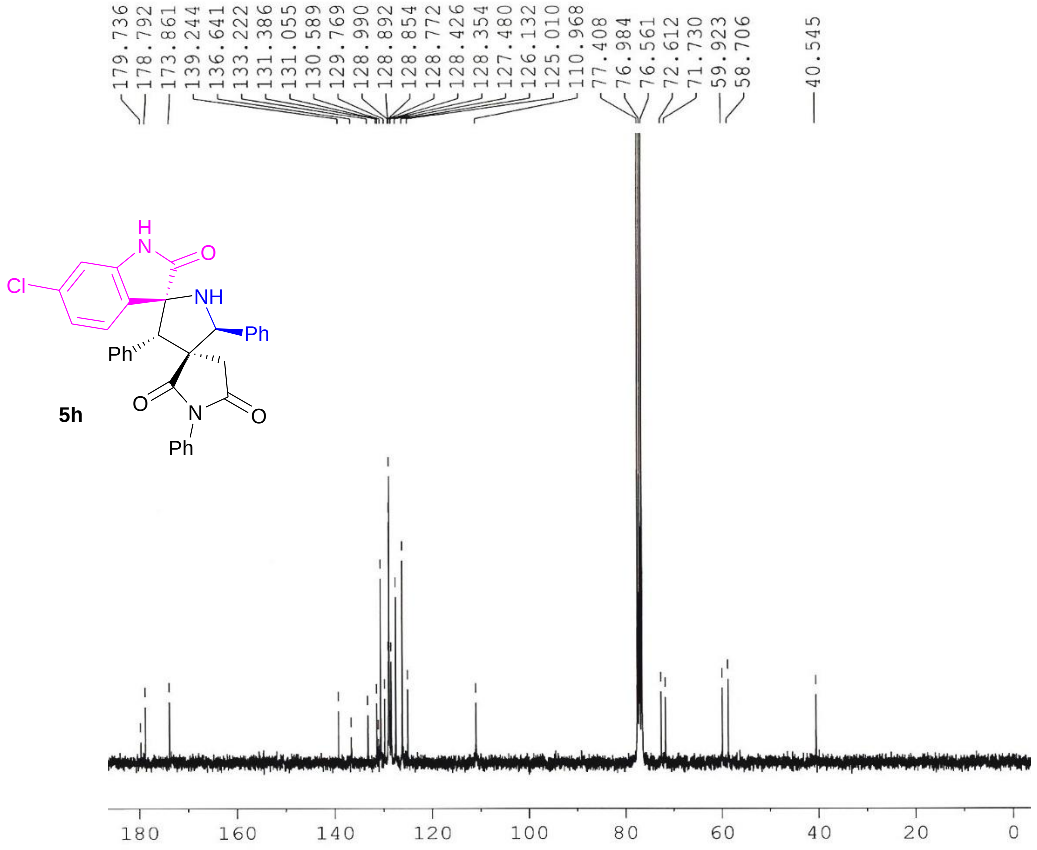

Fig. S24.^{13}C NMR spectrum of **5h** in $CDCl_3$

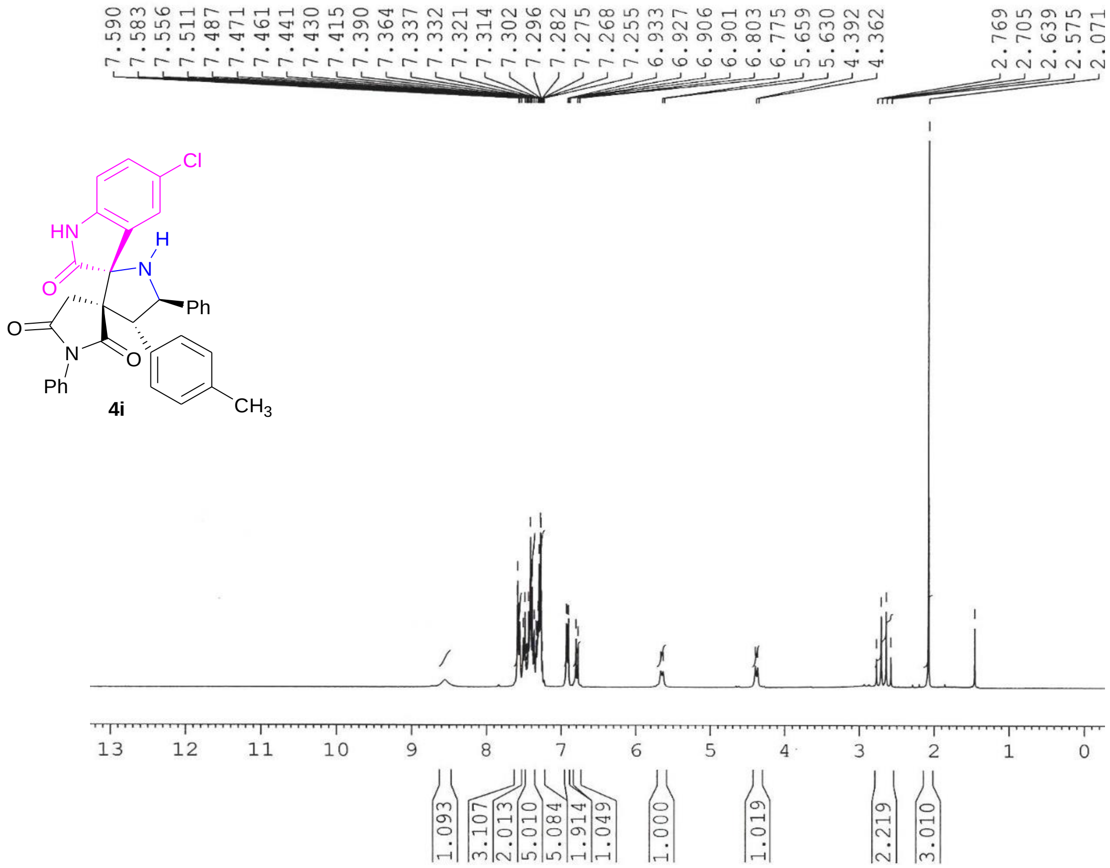

Fig. S25. ^{1}H NMR spectrum of **4i** in $CDCl_3$

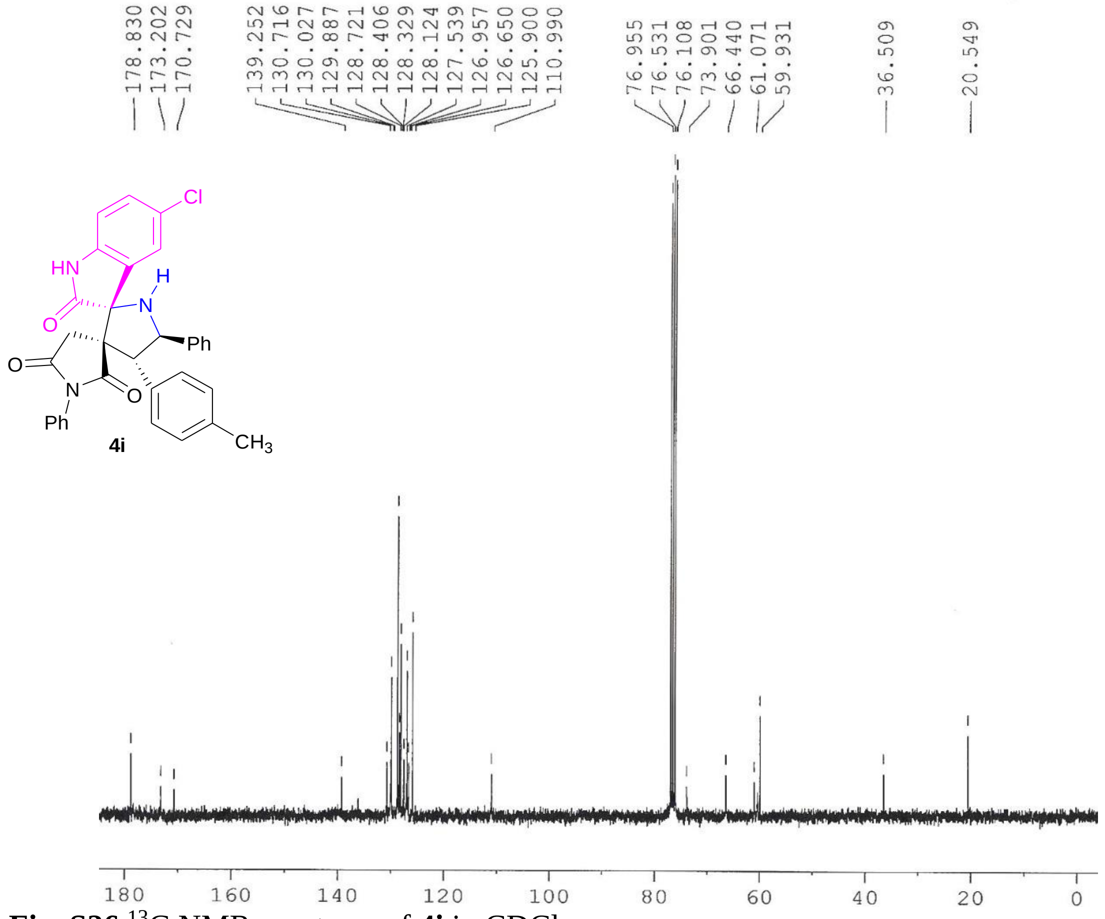

Fig. S26. ^{13}C NMR spectrum of **4i** in $CDCl_3$

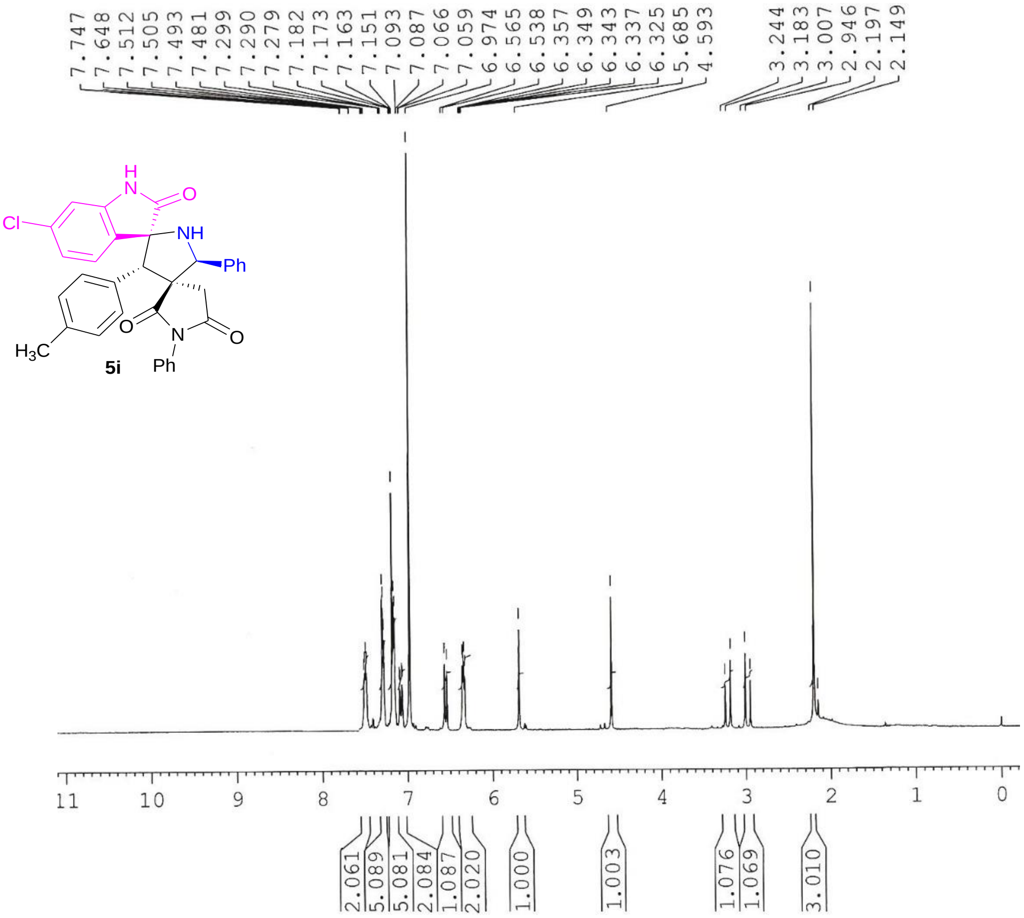

Fig. S27.^{1}H NMR spectrum of **5i** in $CDCl_3$

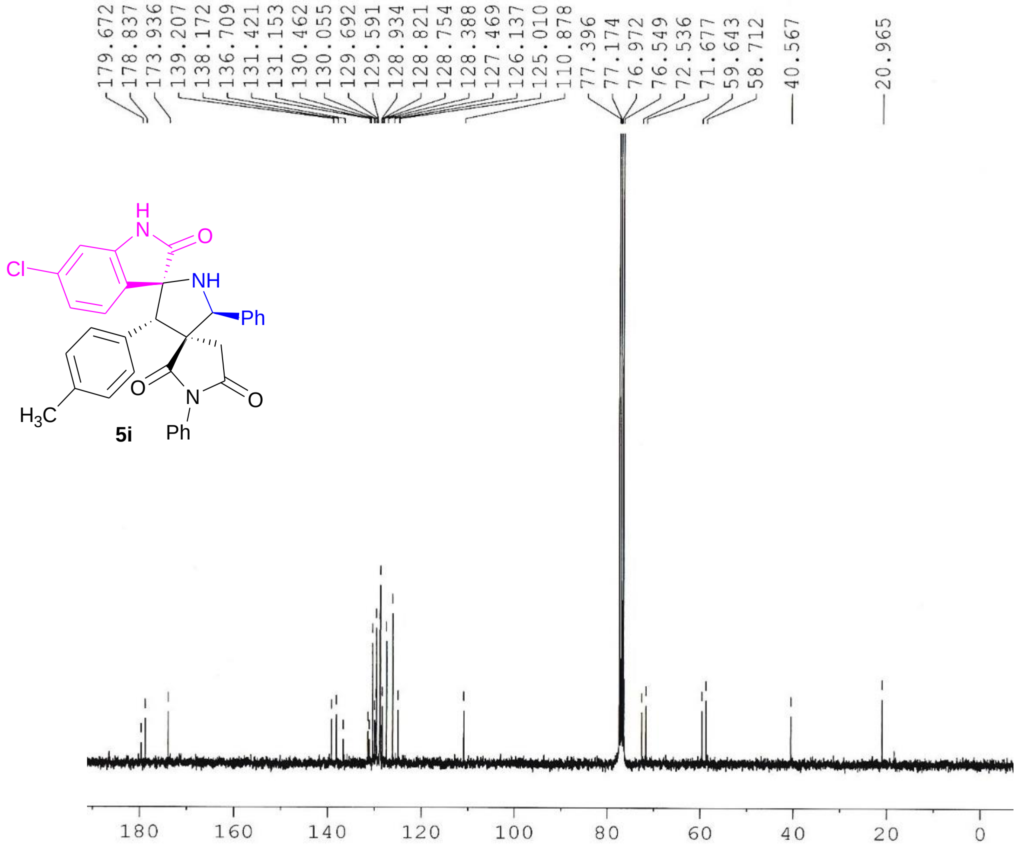

Fig. S28.^{13}C NMR spectrum of **5i** in $CDCl_3$

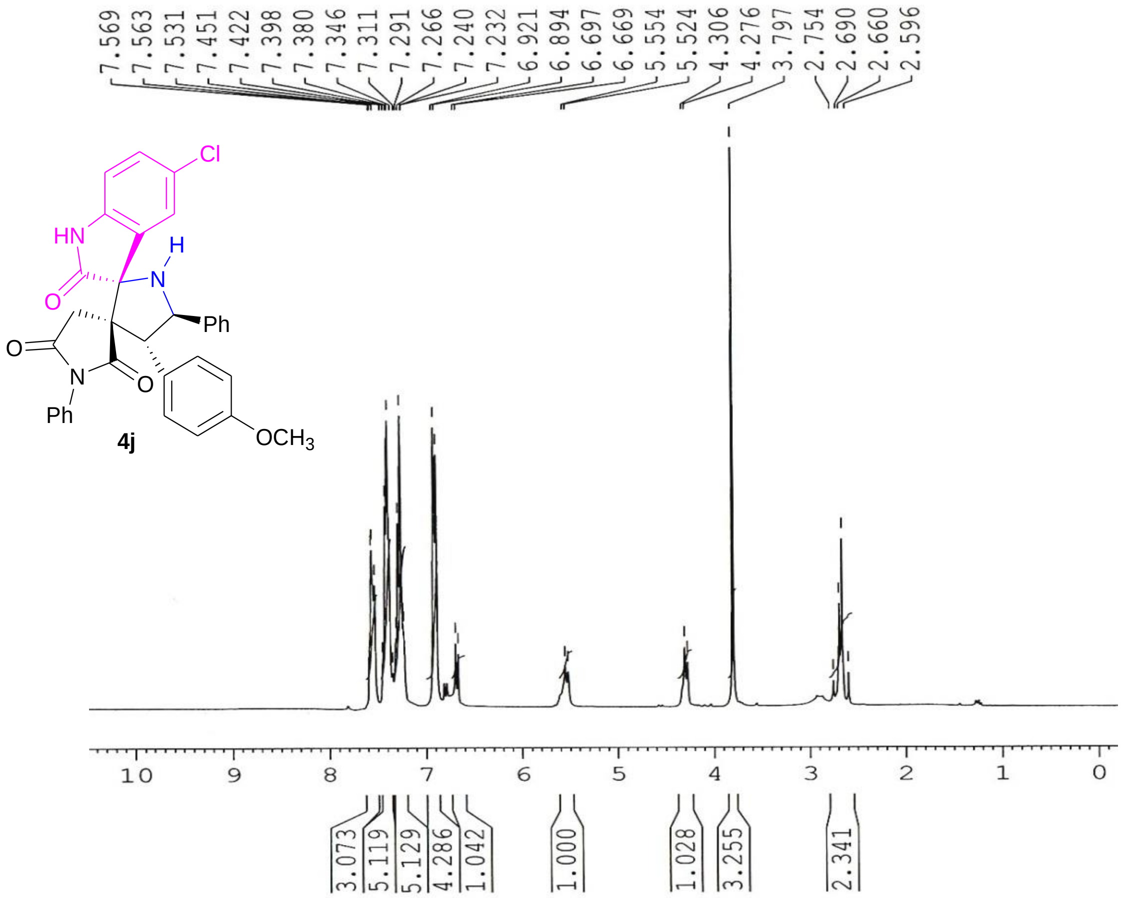

Fig. S29.^{1}H NMR spectrum of **4j** in $CDCl_3$

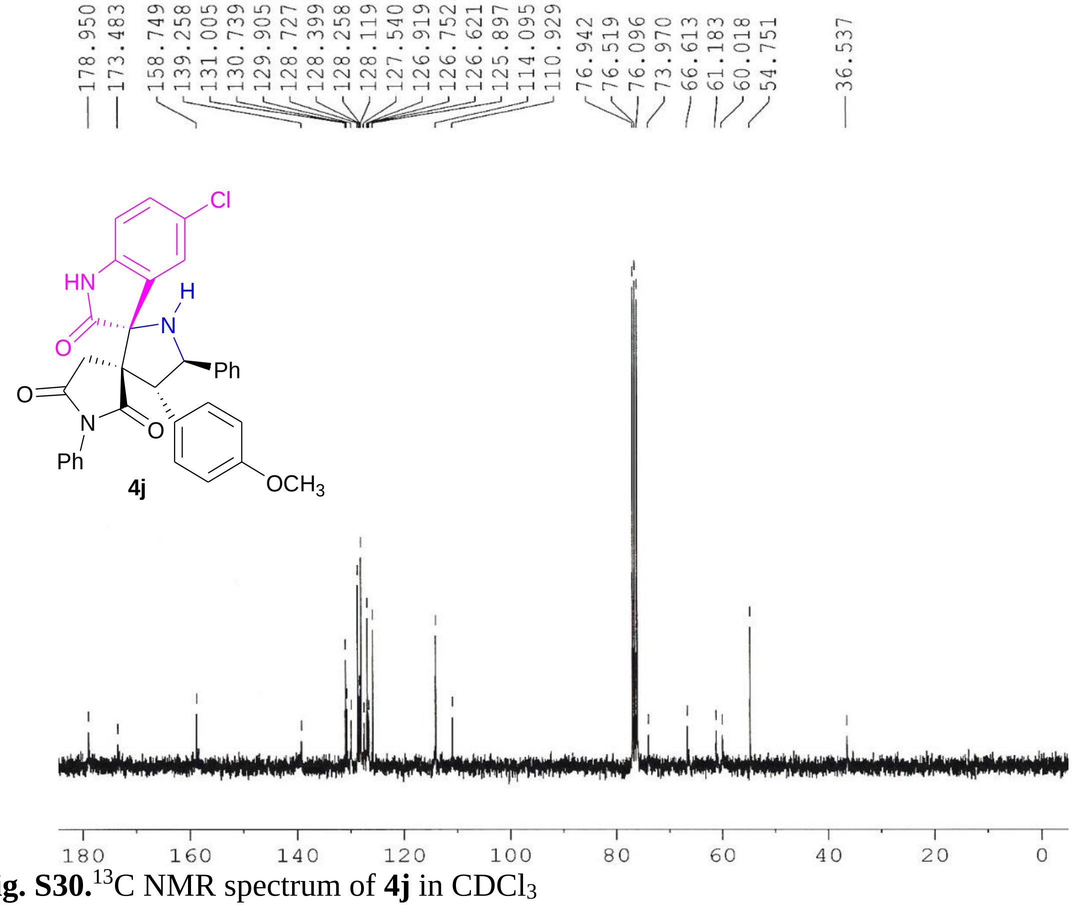

Fig. S30.^{13}C NMR spectrum of **4j** in $CDCl_3$

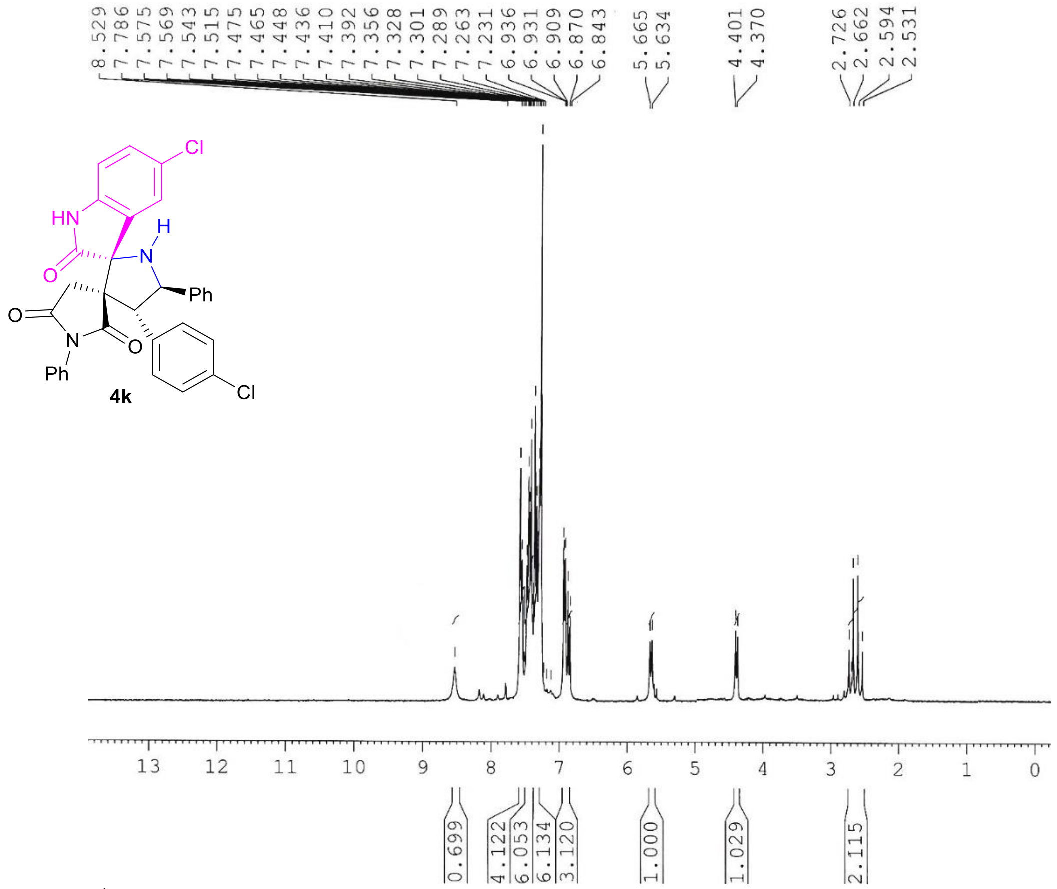

Fig. S31.^{1}H NMR spectrum of **4k** in $CDCl_3$

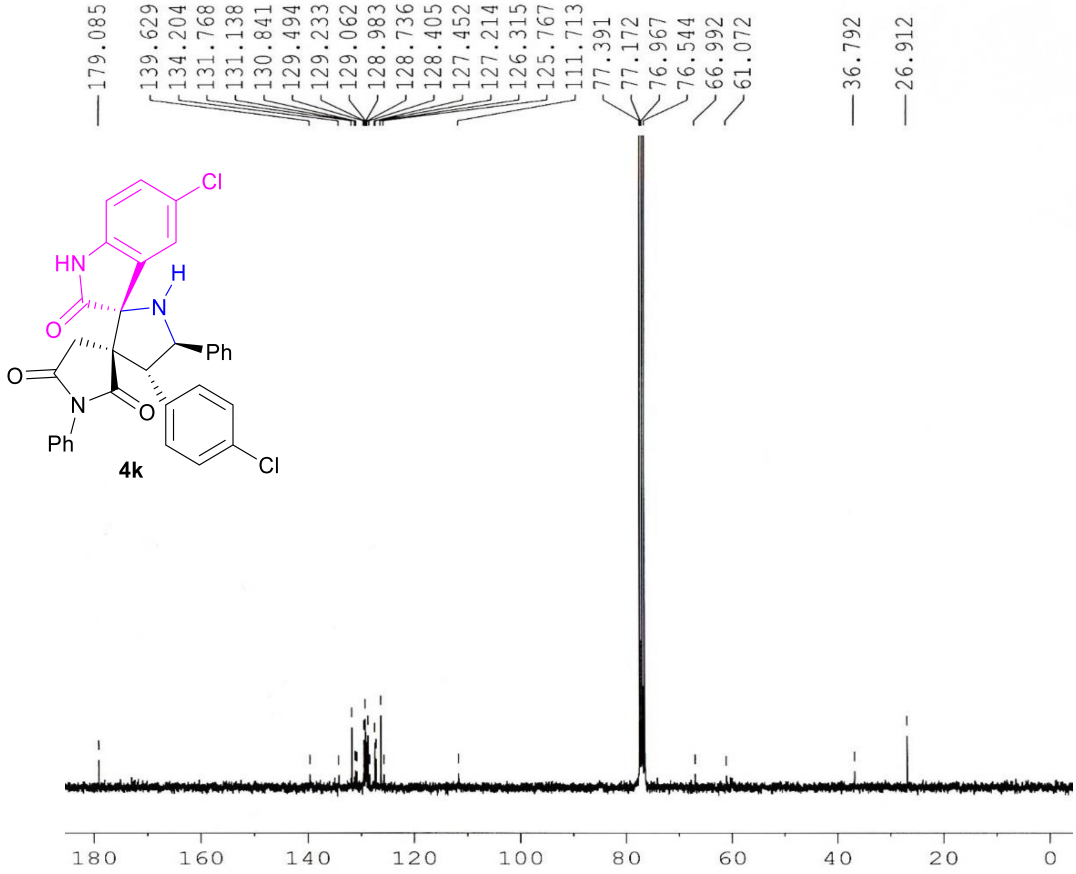

Fig. S32.^{13}C NMR spectrum of **4k** in $CDCl_3$

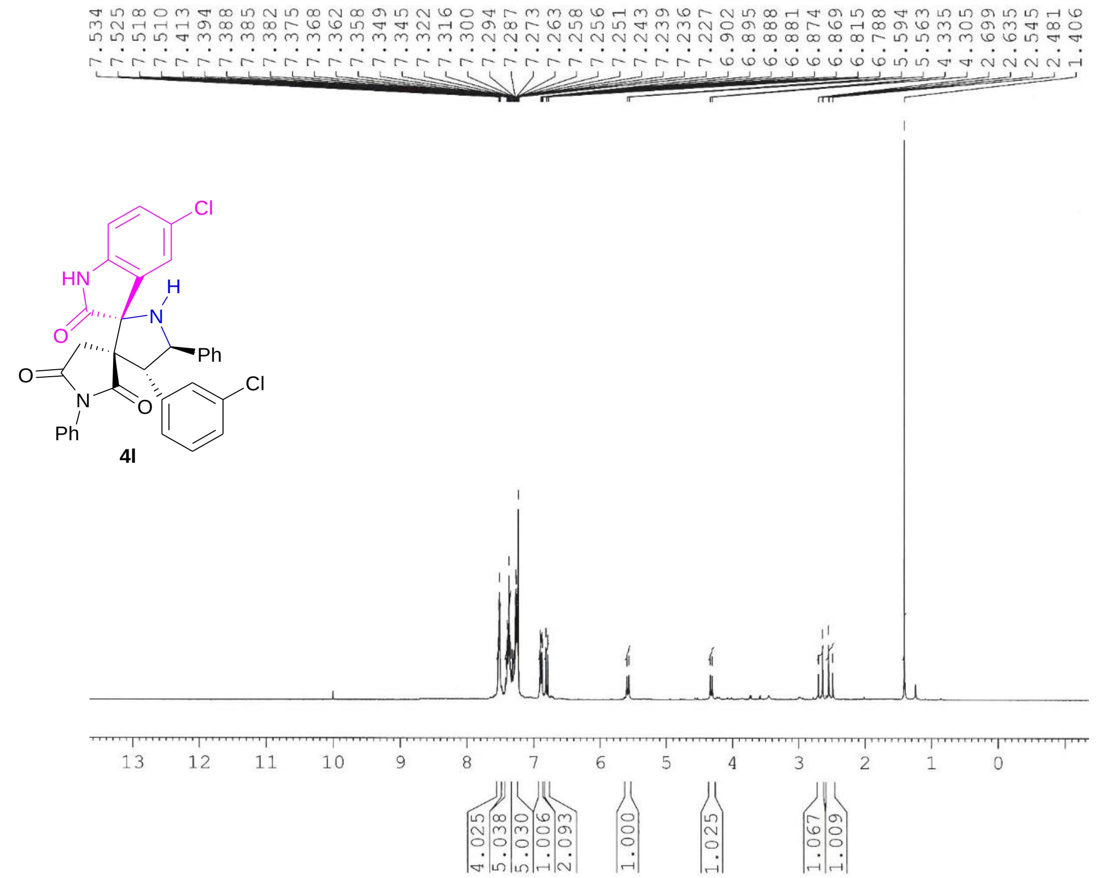

Fig. S33. ^{1}H NMR spectrum of **4l** in $CDCl_3$

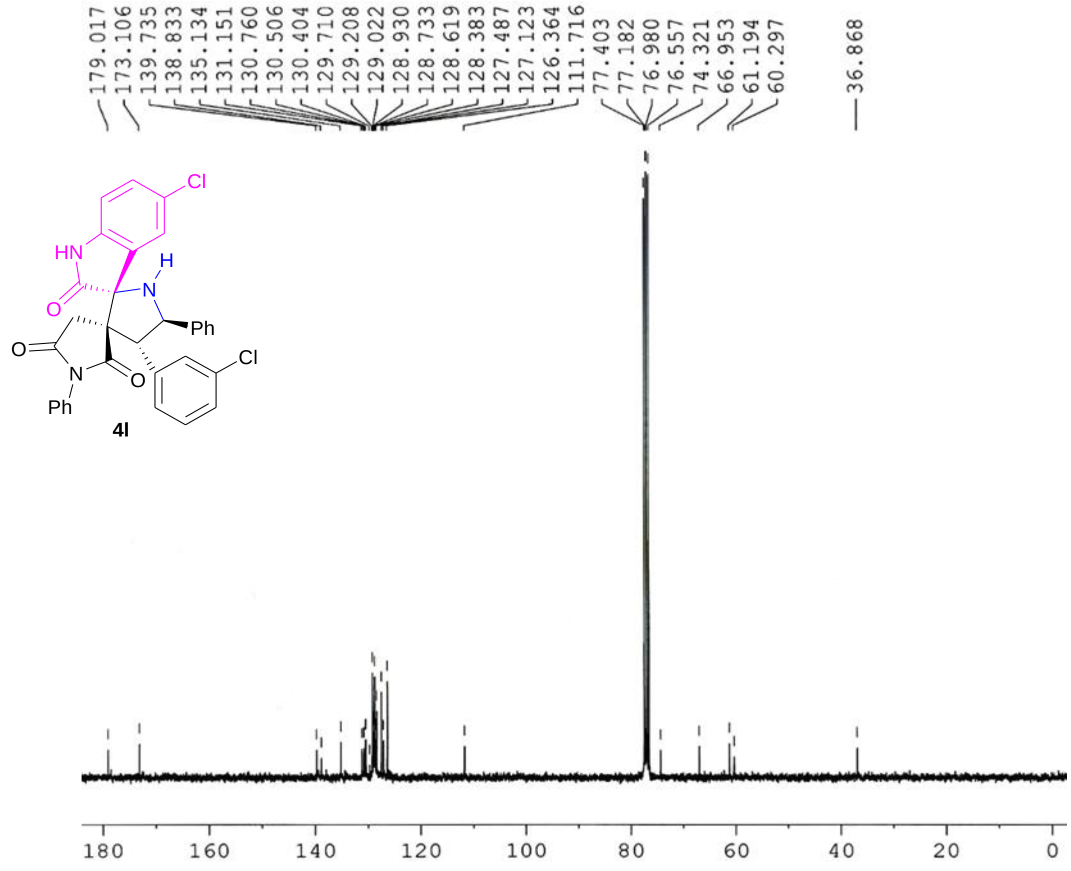

Fig. S34. ^{13}C NMR spectrum of **4l** in $CDCl_3$

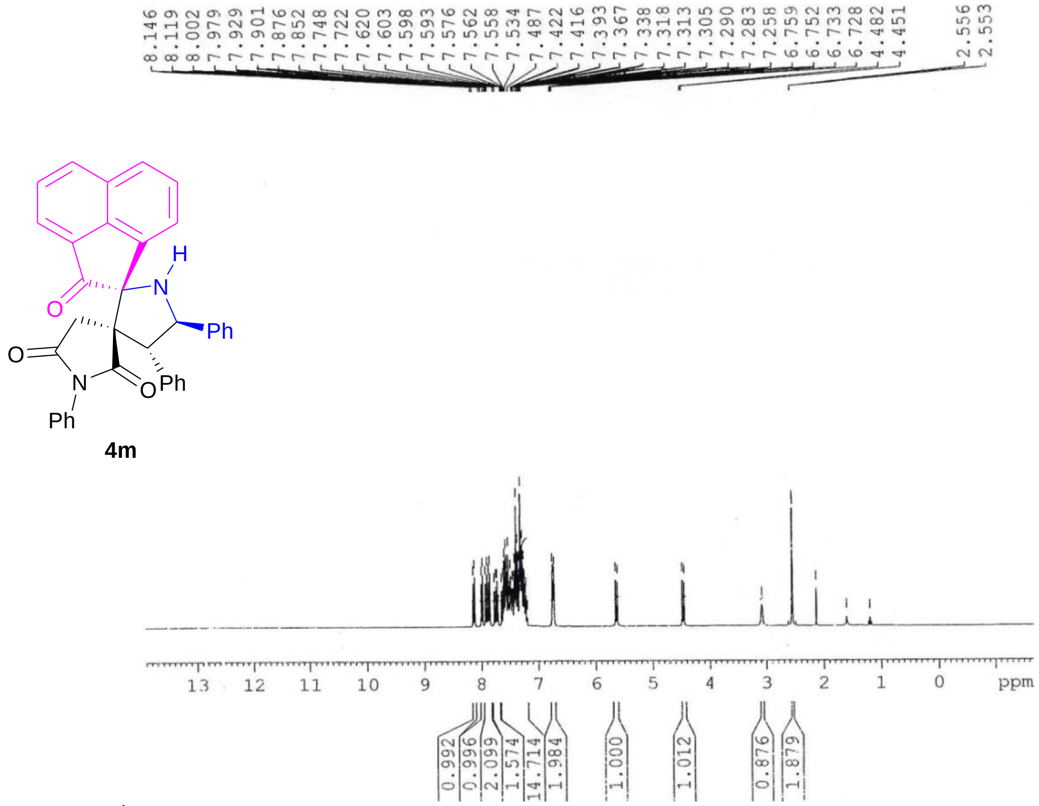

Fig. S35.^{1}H NMR spectrum of **4m** in $CDCl_3$

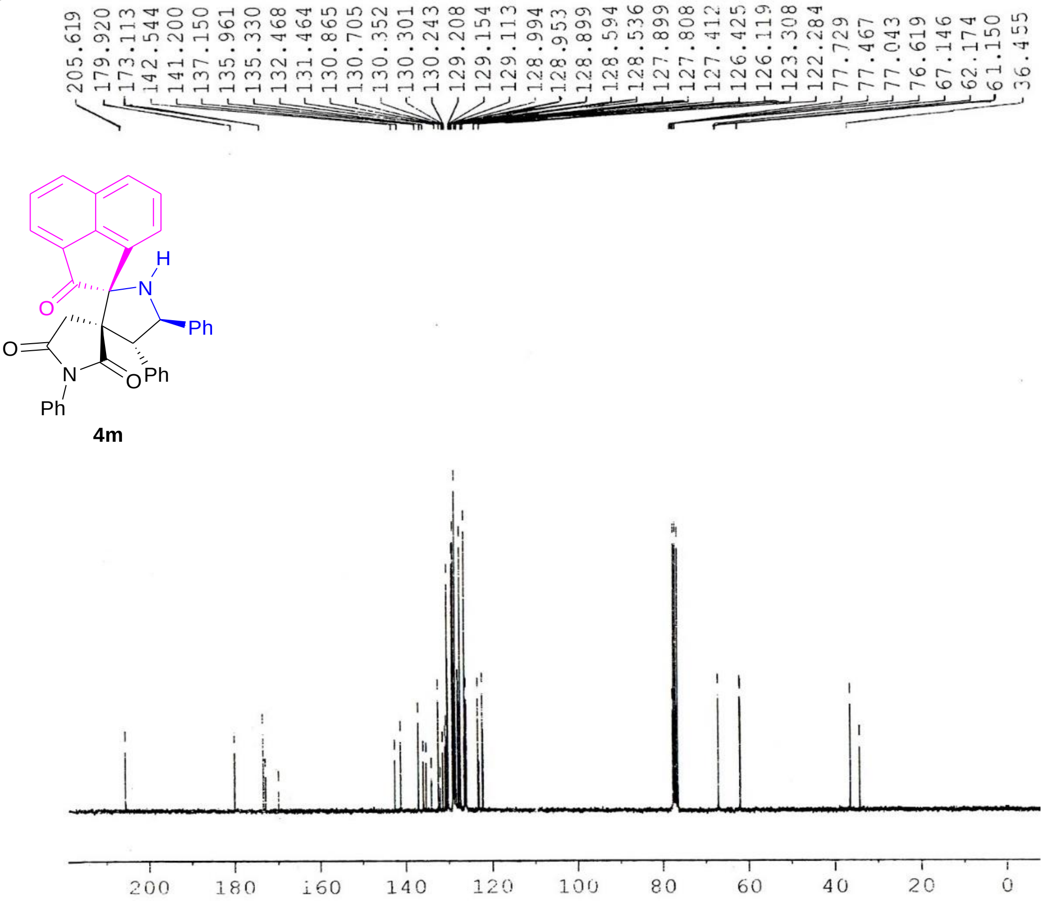

Fig. 36.^{13}C NMR spectrum of **4m** in $CDCl_3$

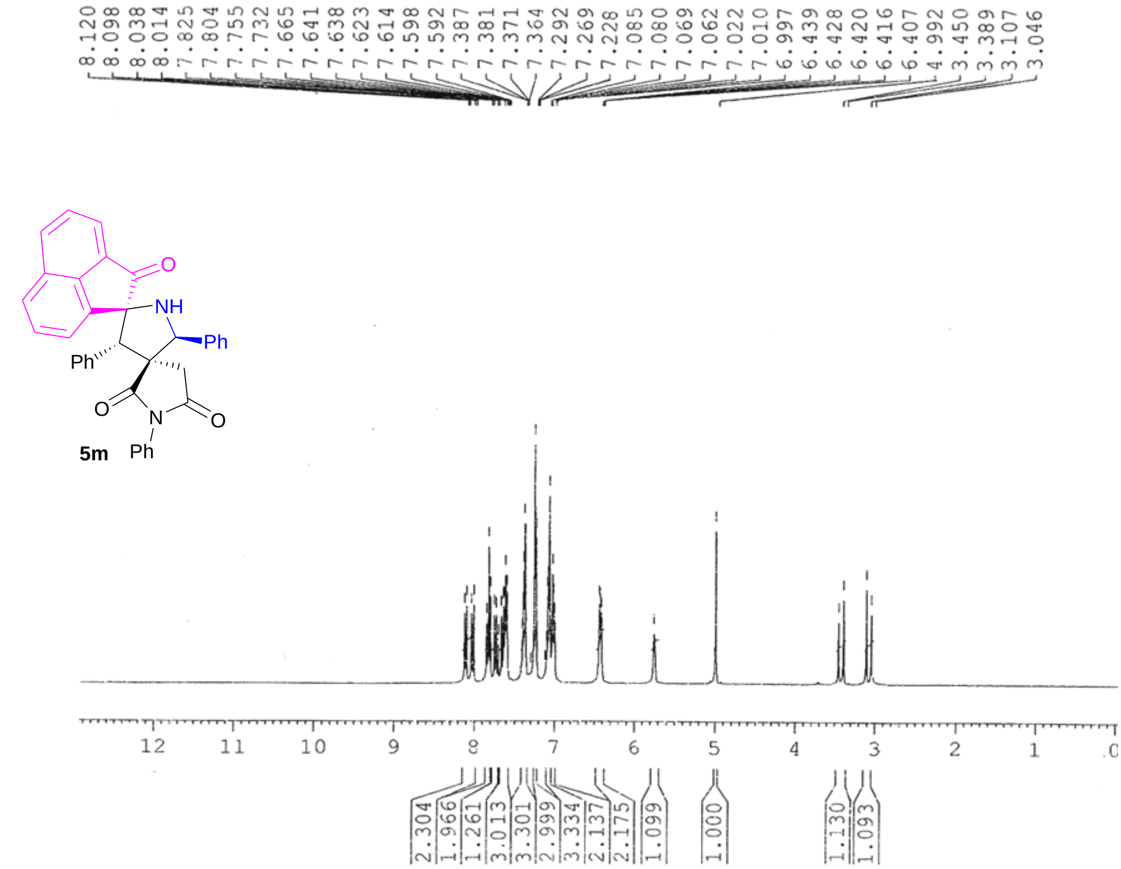

Fig. S37.^{1}H NMR spectrum of **5m** in $CDCl_3$

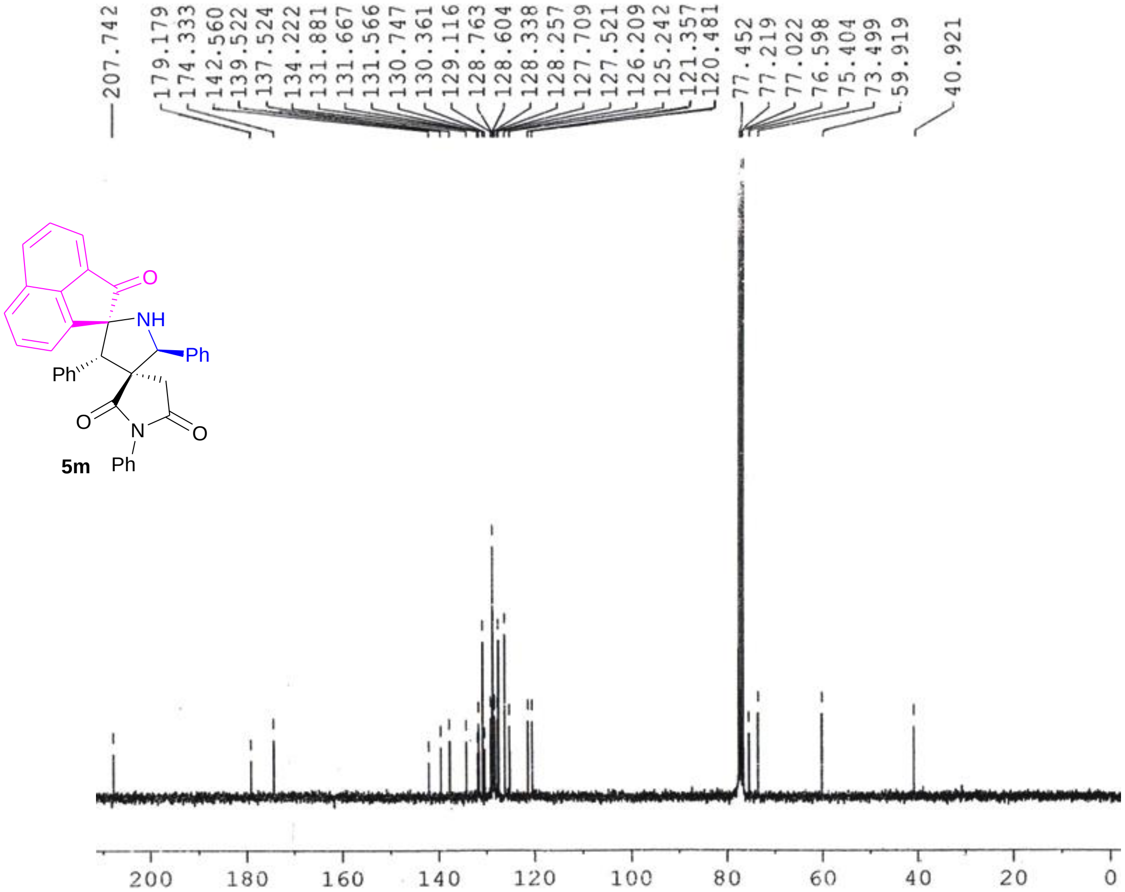

Fig. 38.^{13}C NMR spectrum of **5m** in $CDCl_3$

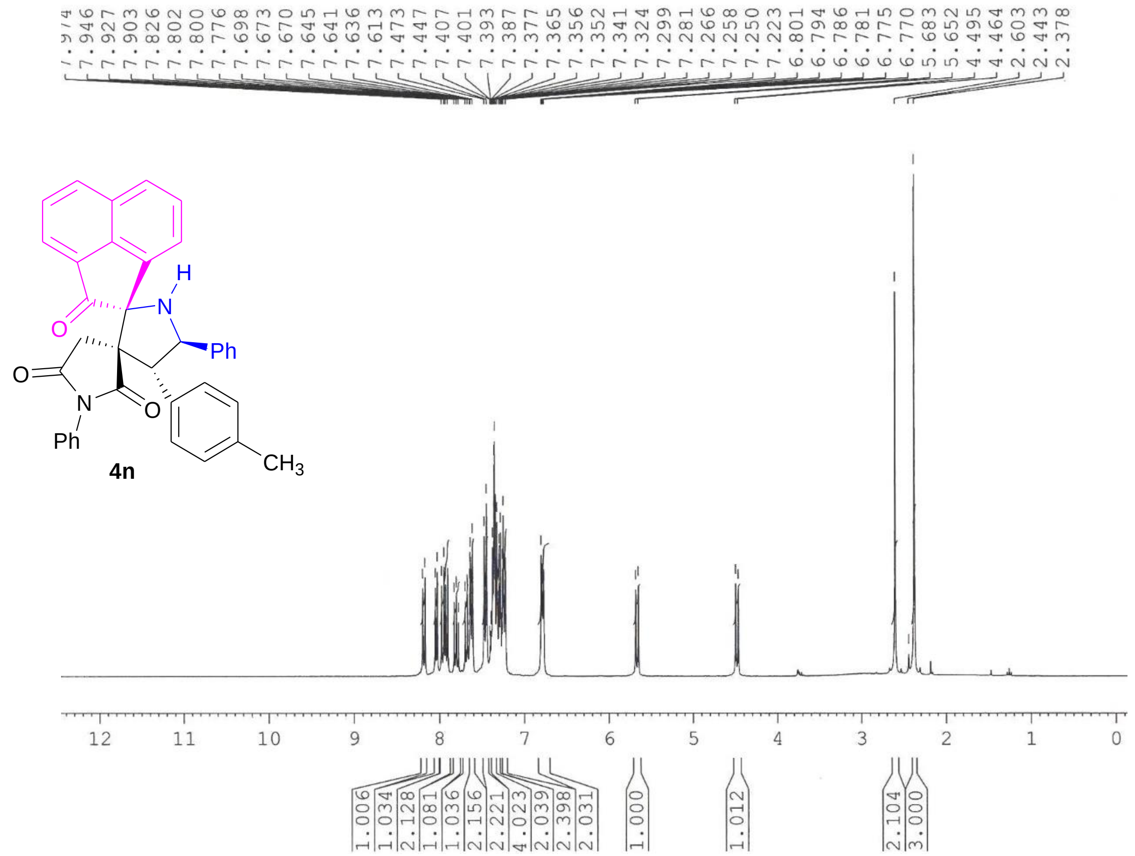

Fig. S39.^{1}H NMR spectrum of **4n** in $CDCl_3$

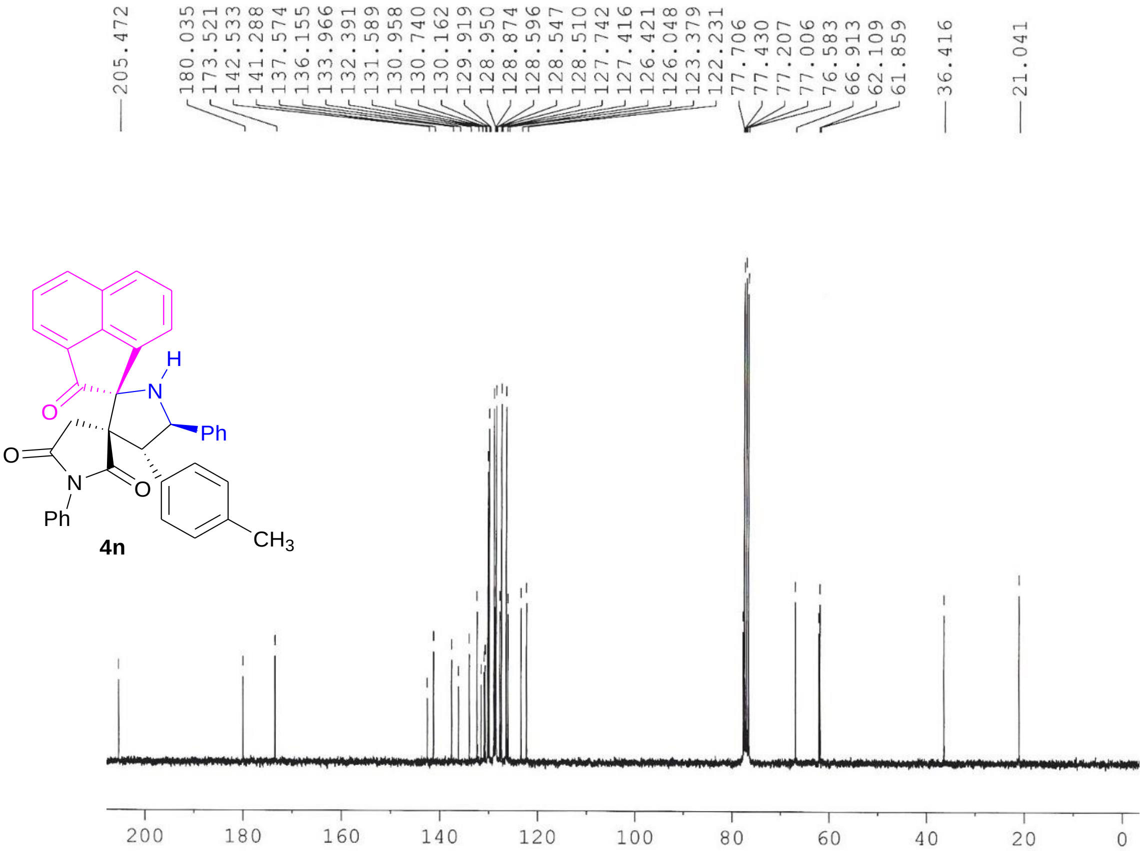

Fig. 40.^{13}C NMR spectrum of **4n** in $CDCl_3$

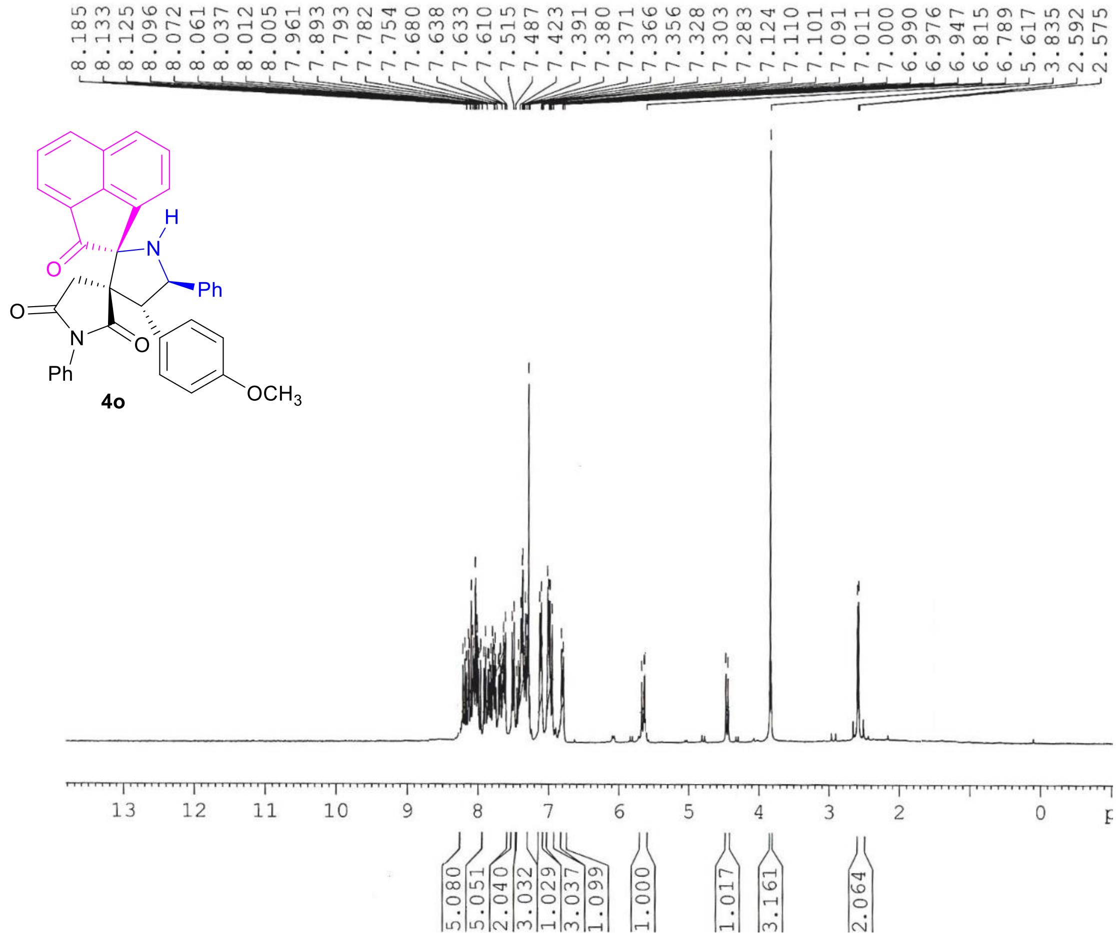

Fig. S41.^{1}H NMR spectrum of **4o** in $CDCl_3$

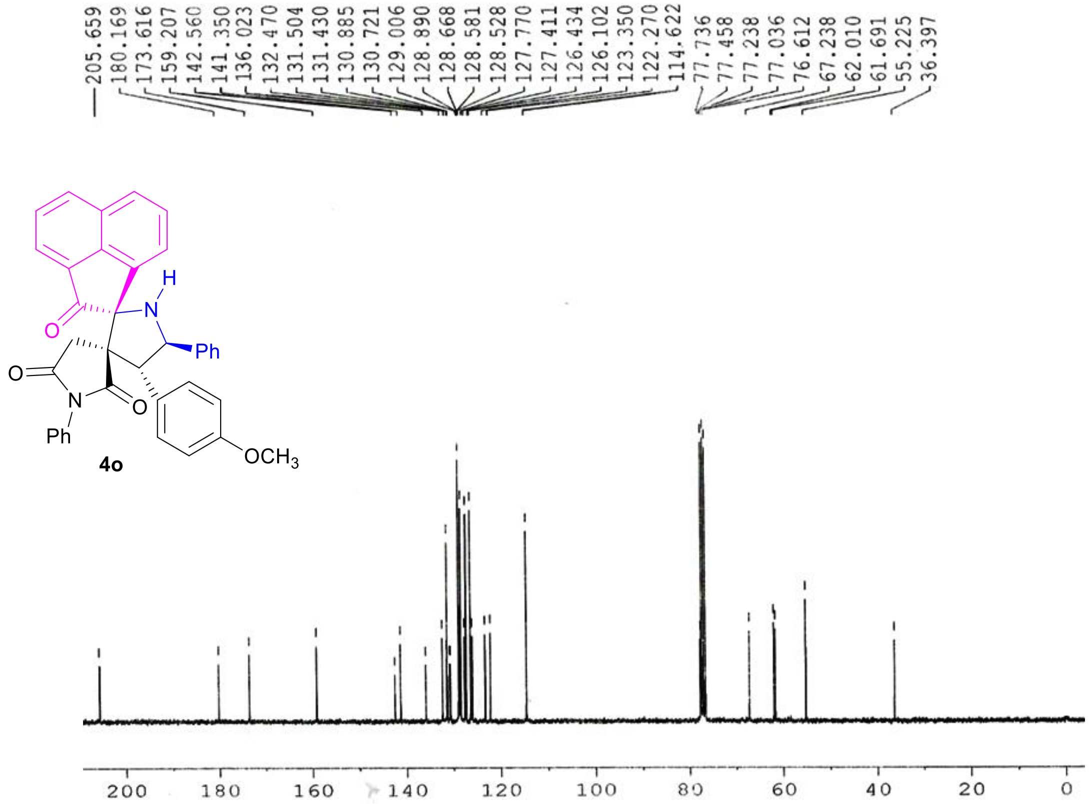

Fig. S42.^{13}C NMR spectrum of **4o** in $CDCl_3$

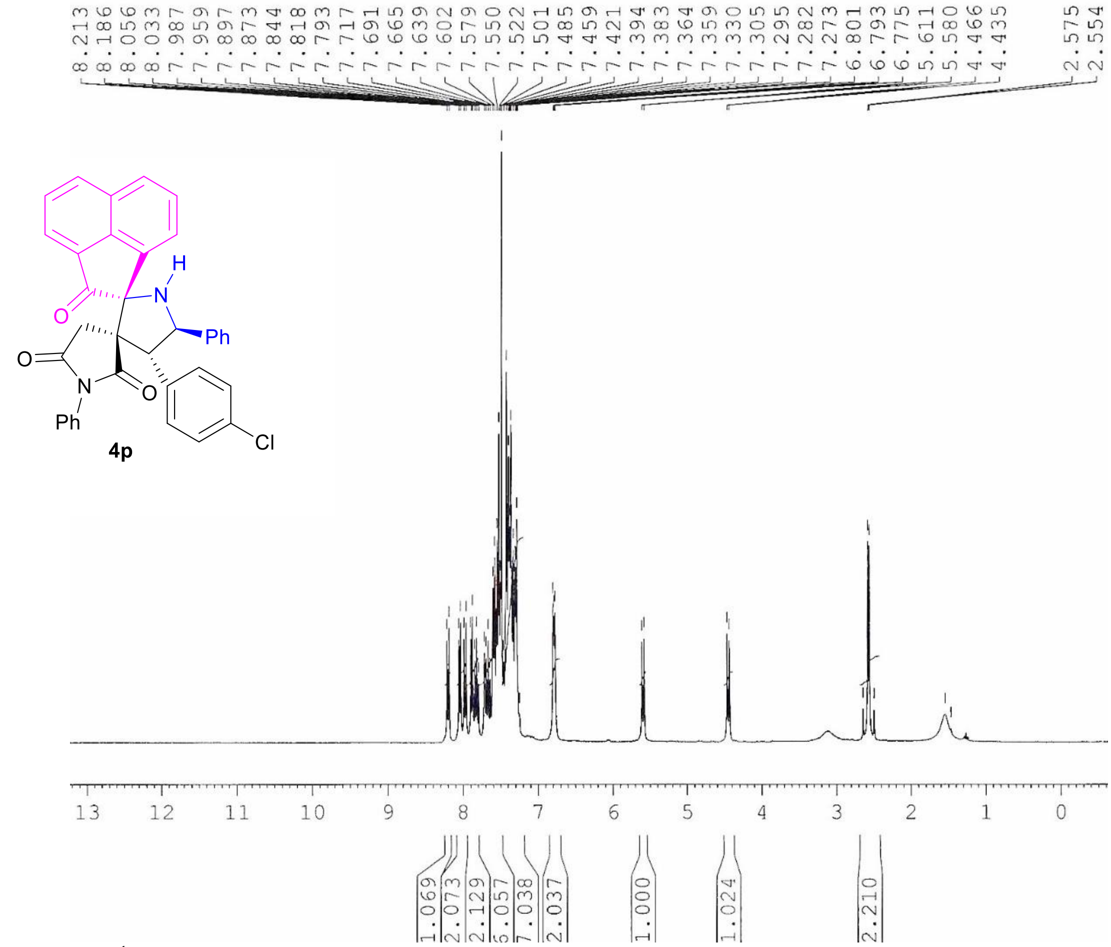

Fig. S43.^{1}H NMR spectrum of **4p** in $CDCl_3$

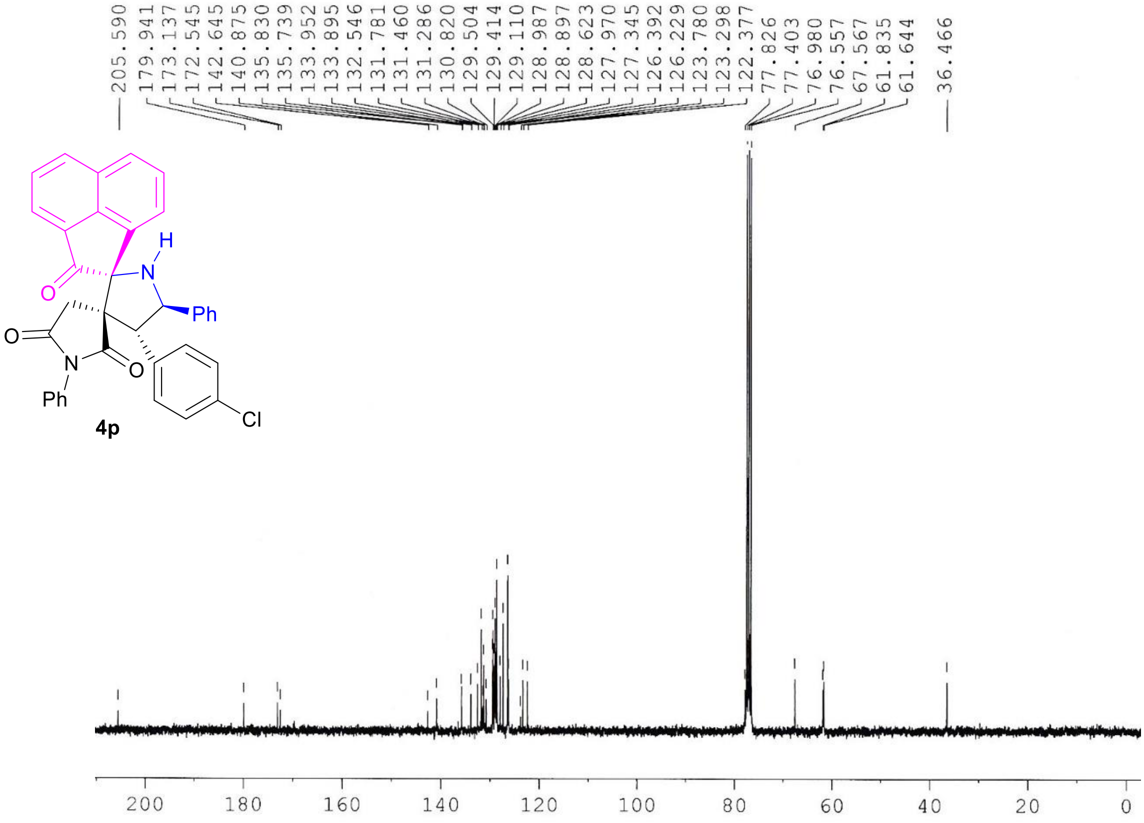

Fig. S44.^{13}C NMR spectrum of **4p** in $CDCl_3$

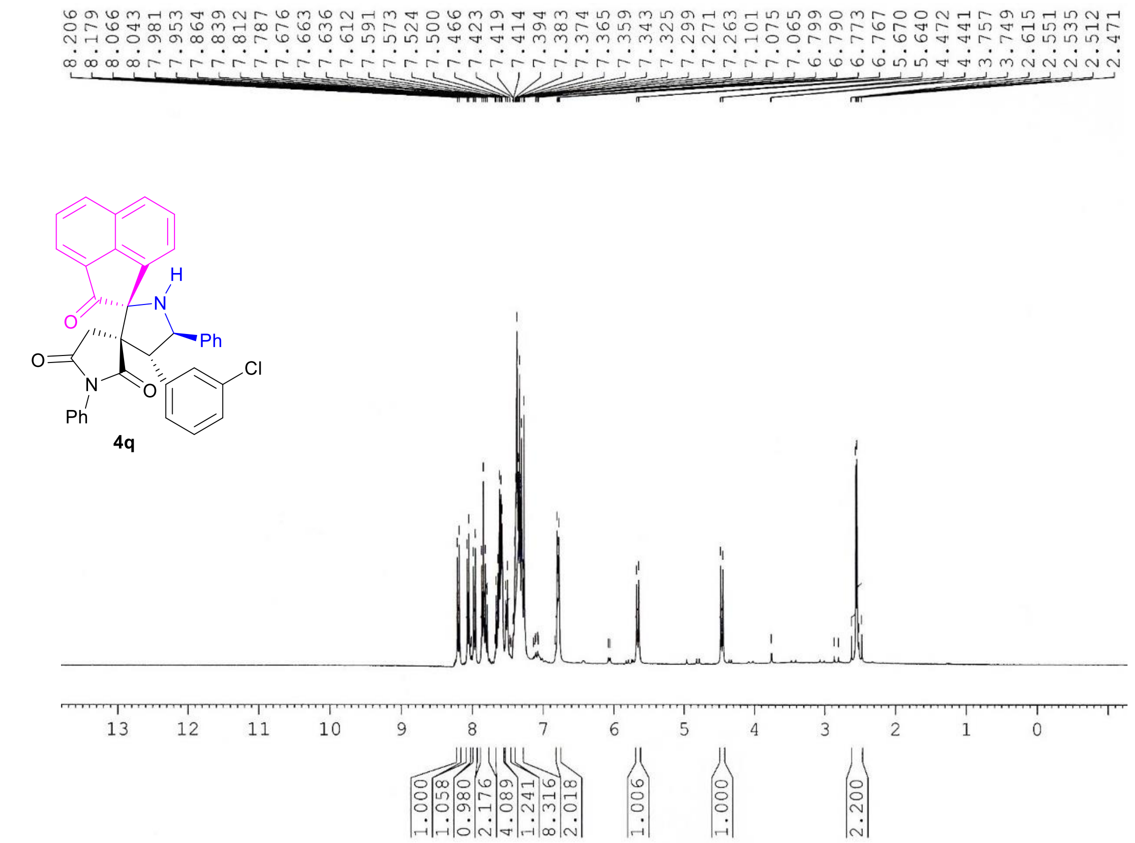

Fig. S45.^{1}H NMR spectrum of **4q** in $CDCl_3$

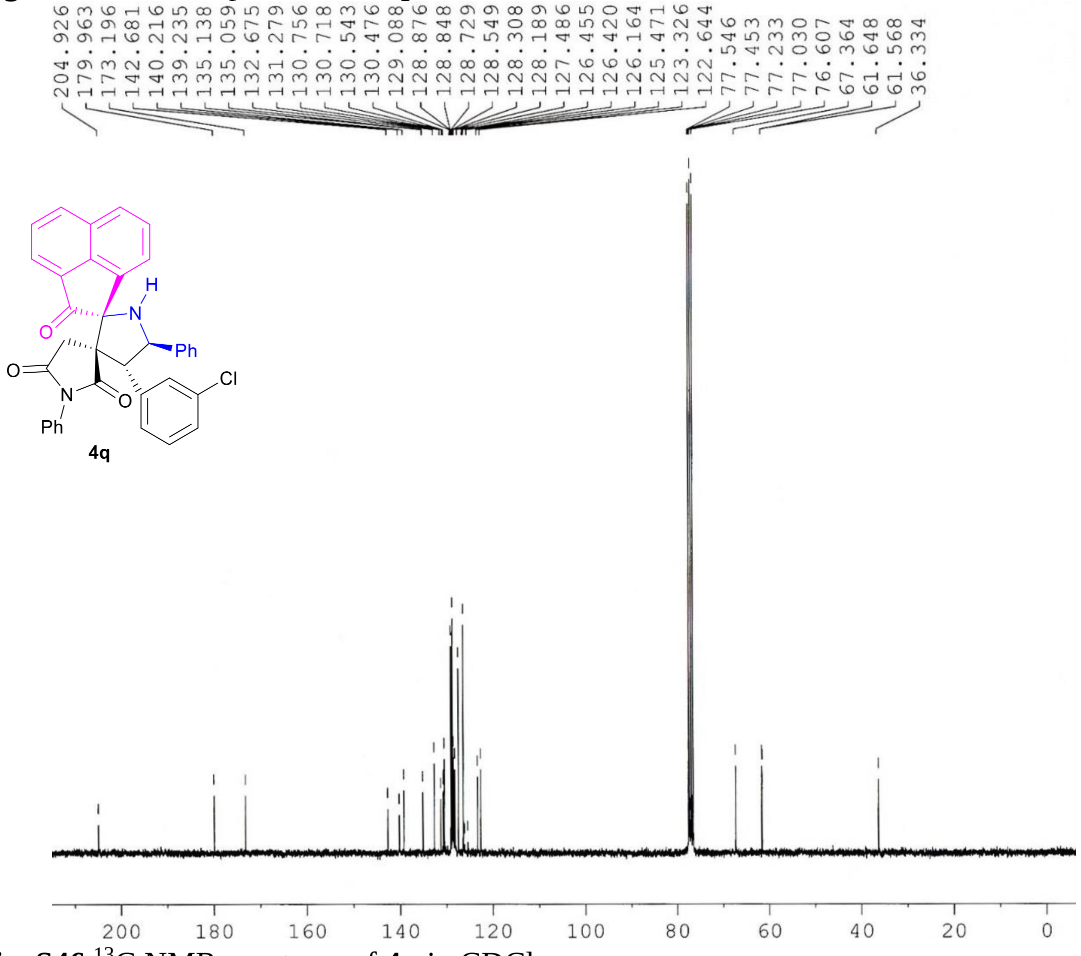

Fig. S46.^{13}C NMR spectrum of **4q** in $CDCl_3$

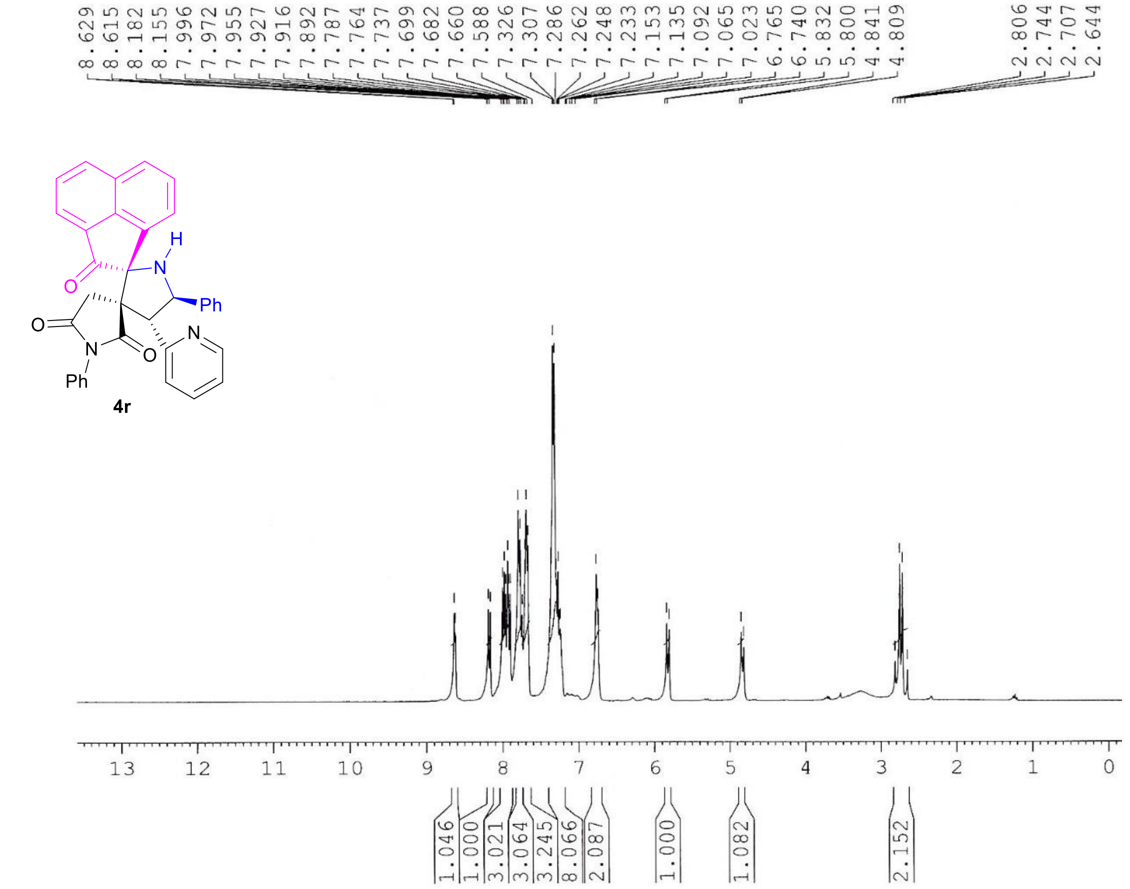

Fig. S47.^{1}H NMR spectrum of **4r** in $CDCl_3$

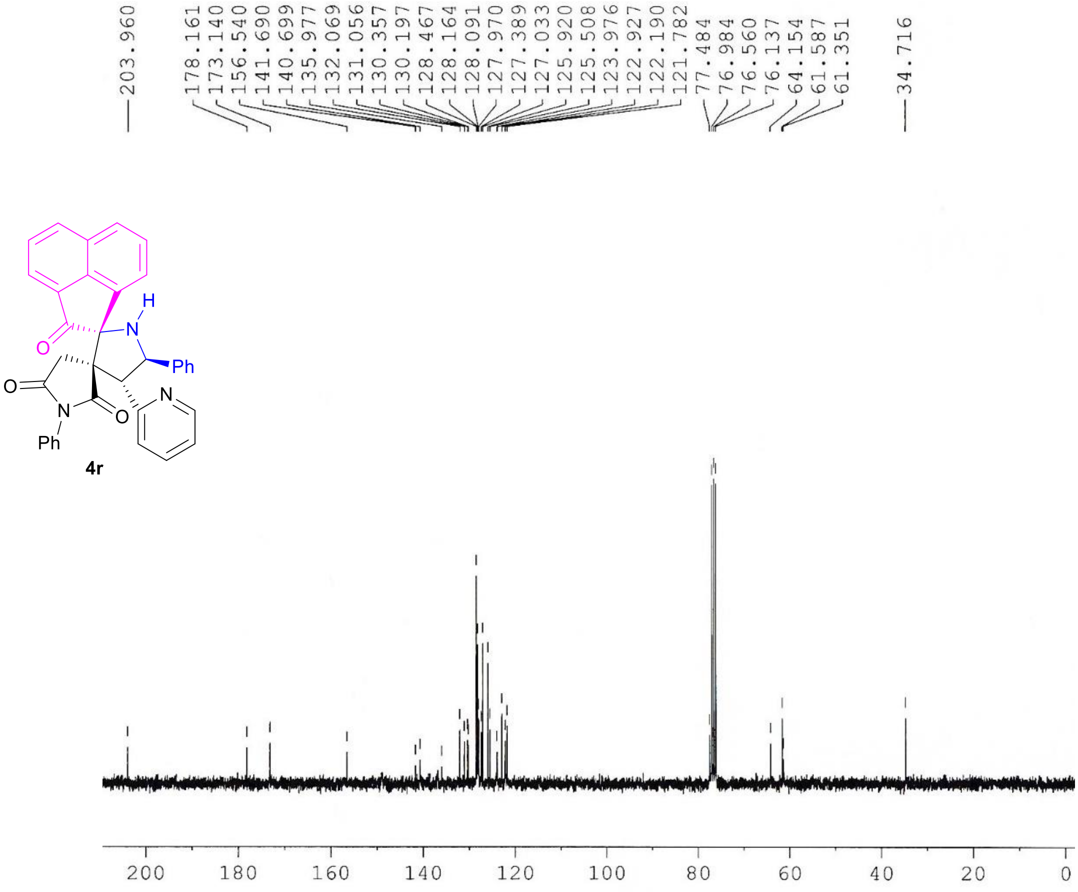

Fig. S48.^{13}C NMR spectrum of **4r** in $CDCl_3$

2. ^{1}H- and ^{13}C-NMR Spectra of compounds 7a-h

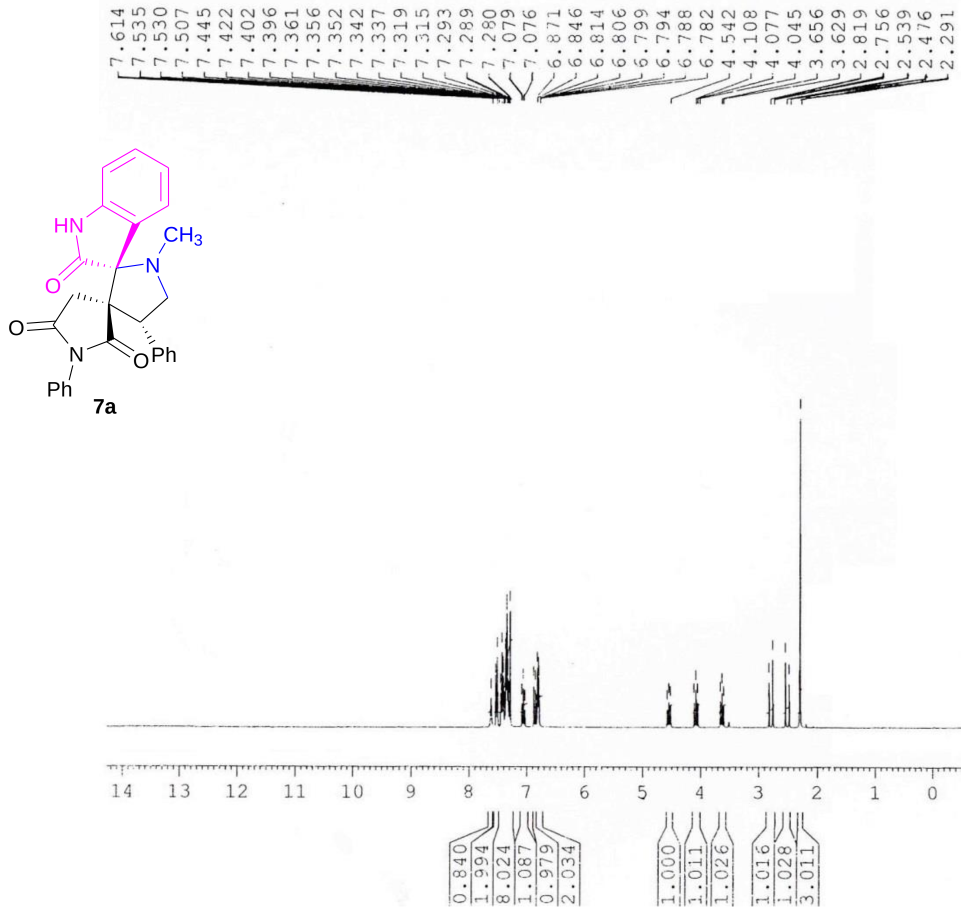

Fig. S49. ^{1}H NMR spectrum of **7a** in $CDCl_3$

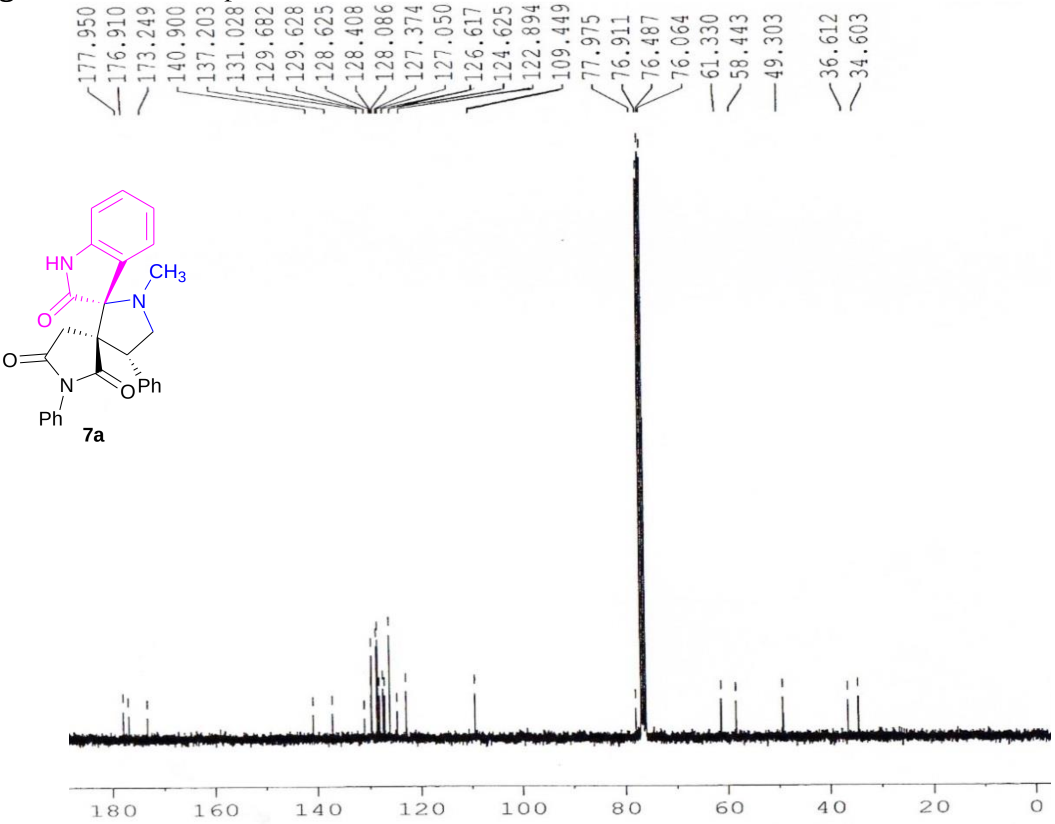

Fig. S50. ^{13}C NMR spectrum of **7a** in $CDCl_3$

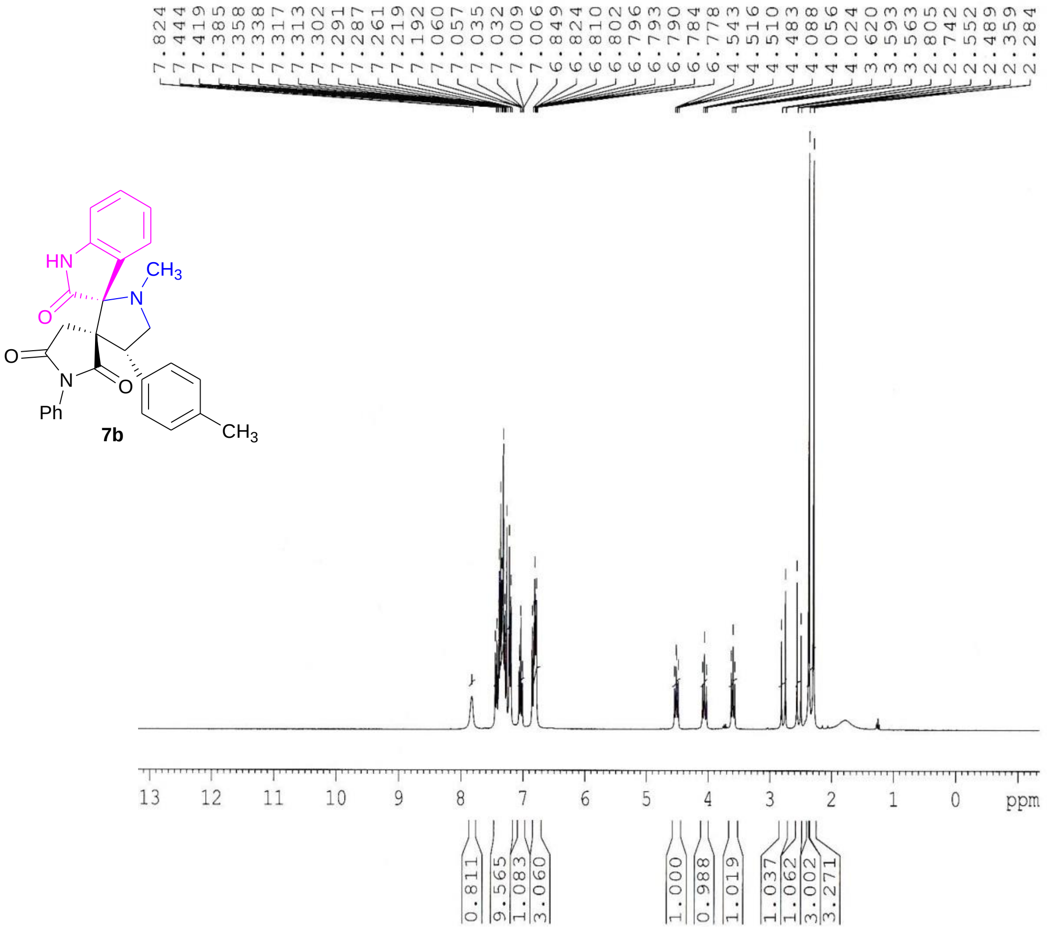

Fig. S51.^{1}H NMR spectrum of **7b** in $CDCl_3$

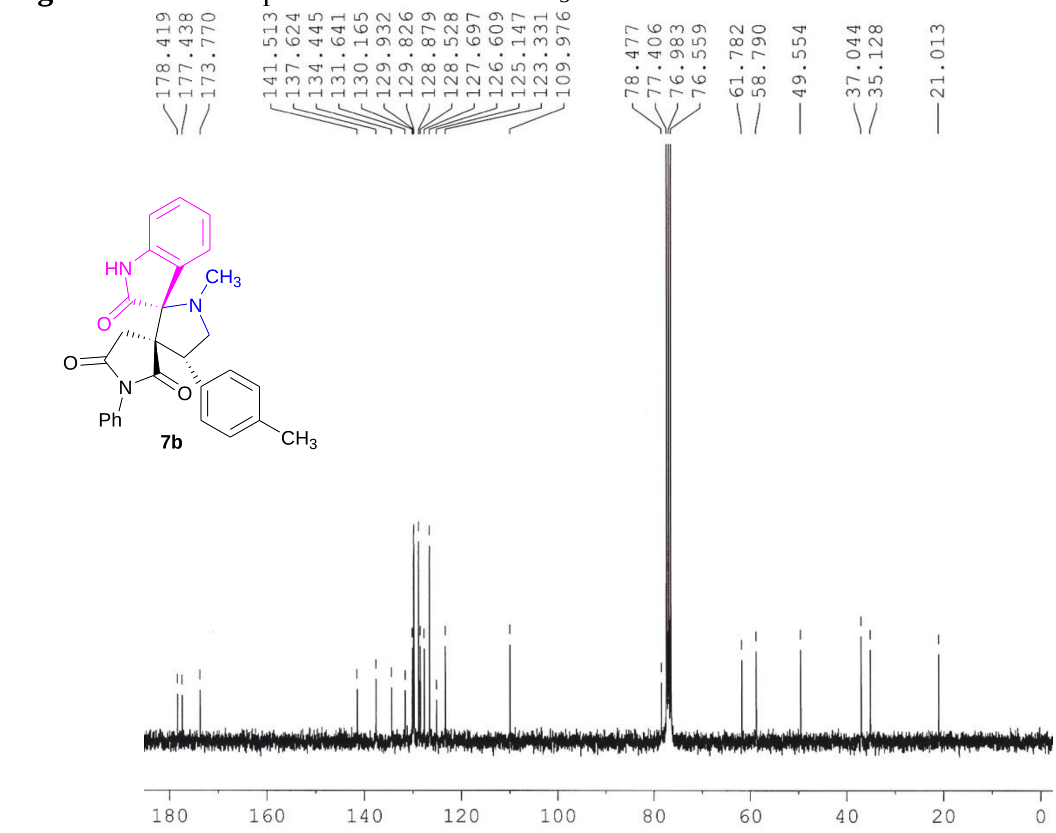

Fig. S52.^{13}C NMR spectrum of **7b** in $CDCl_3$

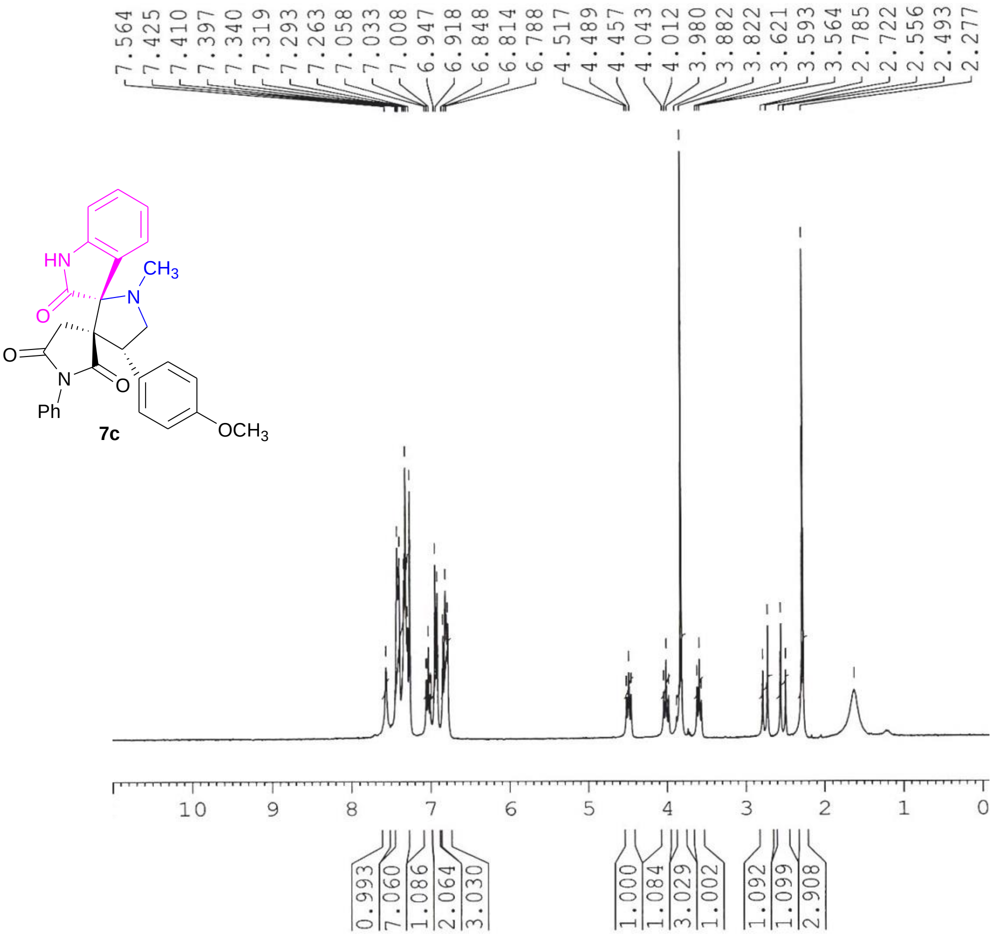

Fig. S53.^{1}H NMR spectrum of **7c** in $CDCl_3$

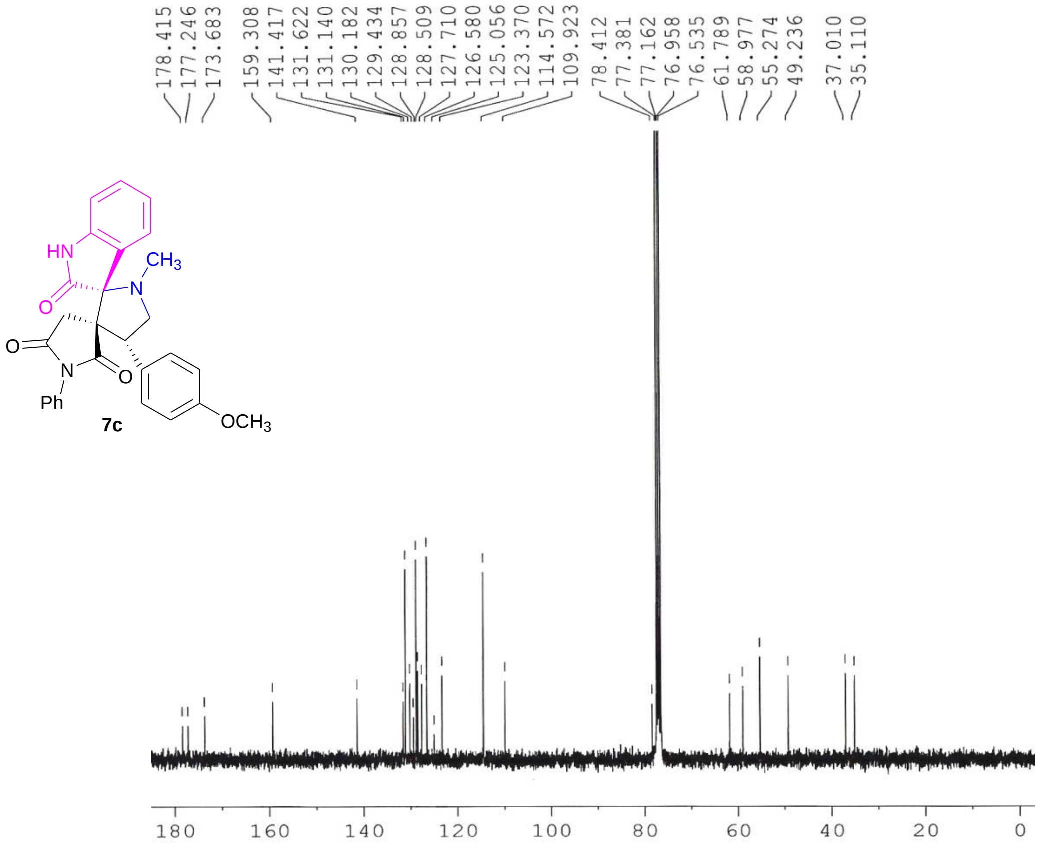

Fig. S54.^{13}C NMR spectrum of **7c** in $CDCl_3$

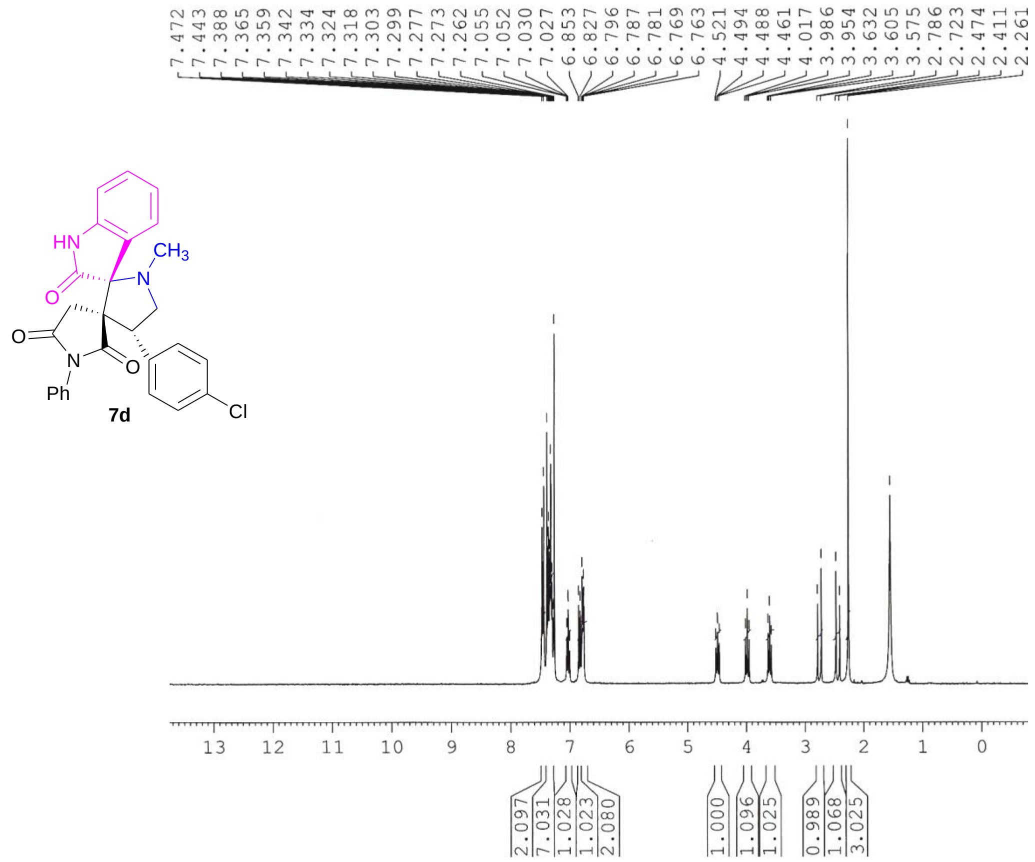

Fig. S55. ^{1}H NMR spectrum of **7d** in $CDCl_3$

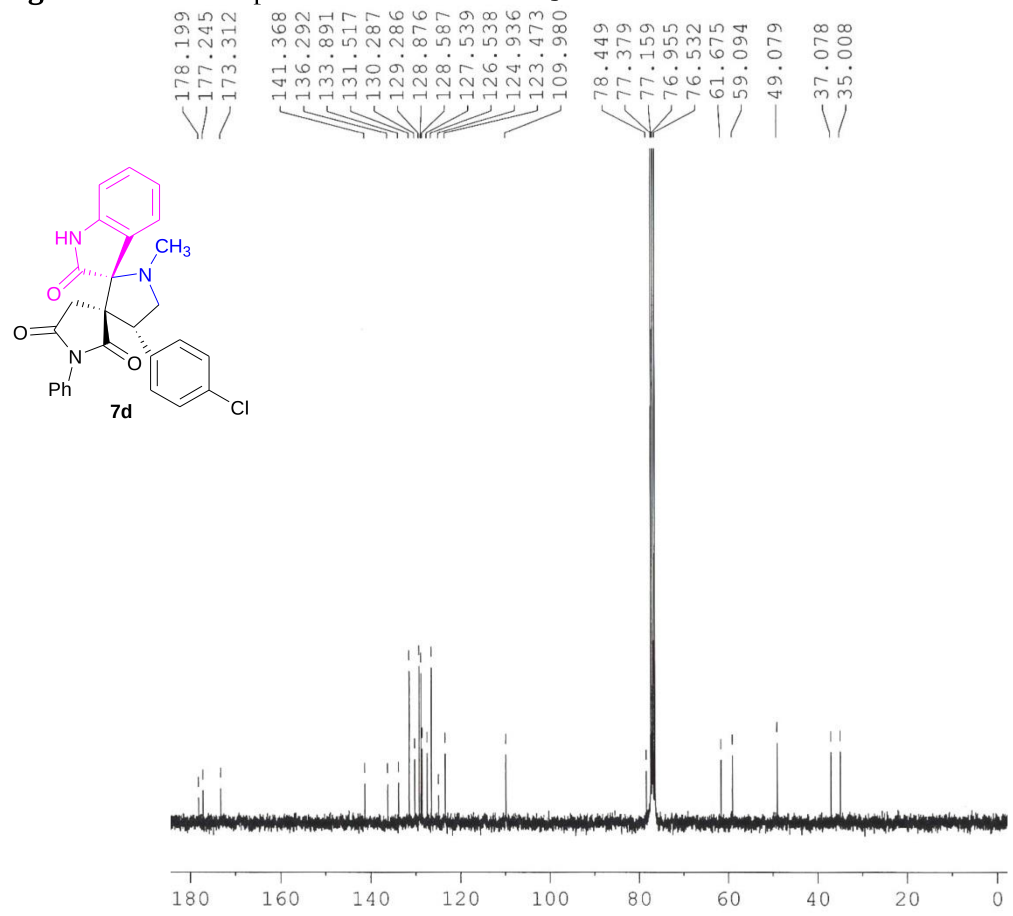

Fig. S56. ^{13}C NMR spectrum of **7d** in $CDCl_3$

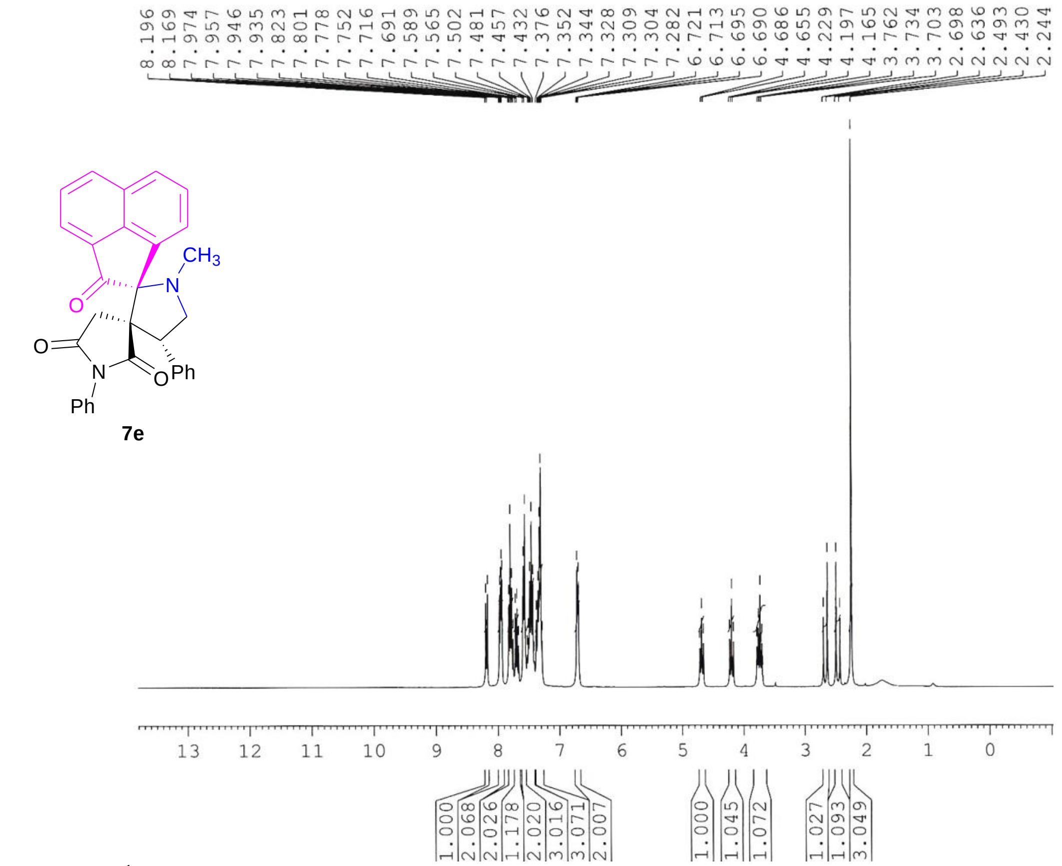

Fig. S57.^{1}H NMR spectrum of **7e** in $CDCl_3$

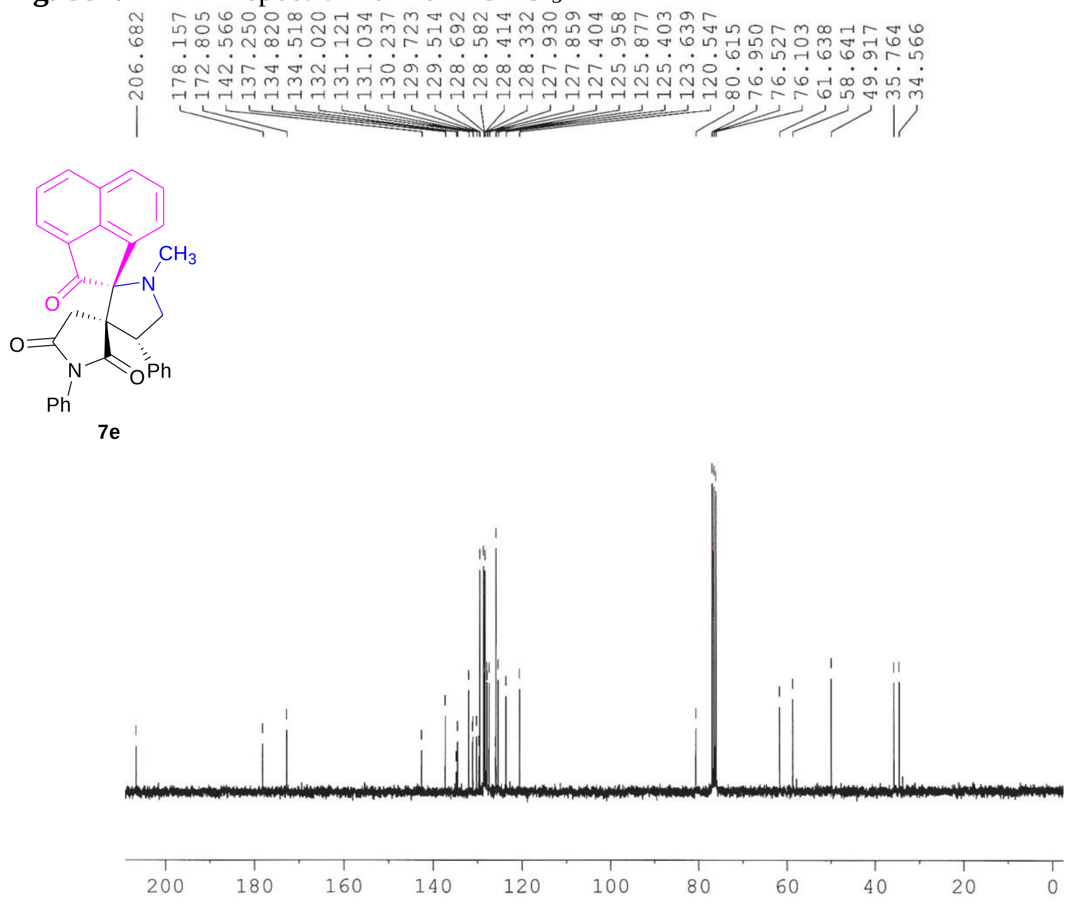

Fig. S58.^{13}C NMR spectrum of **7e** in $CDCl_3$

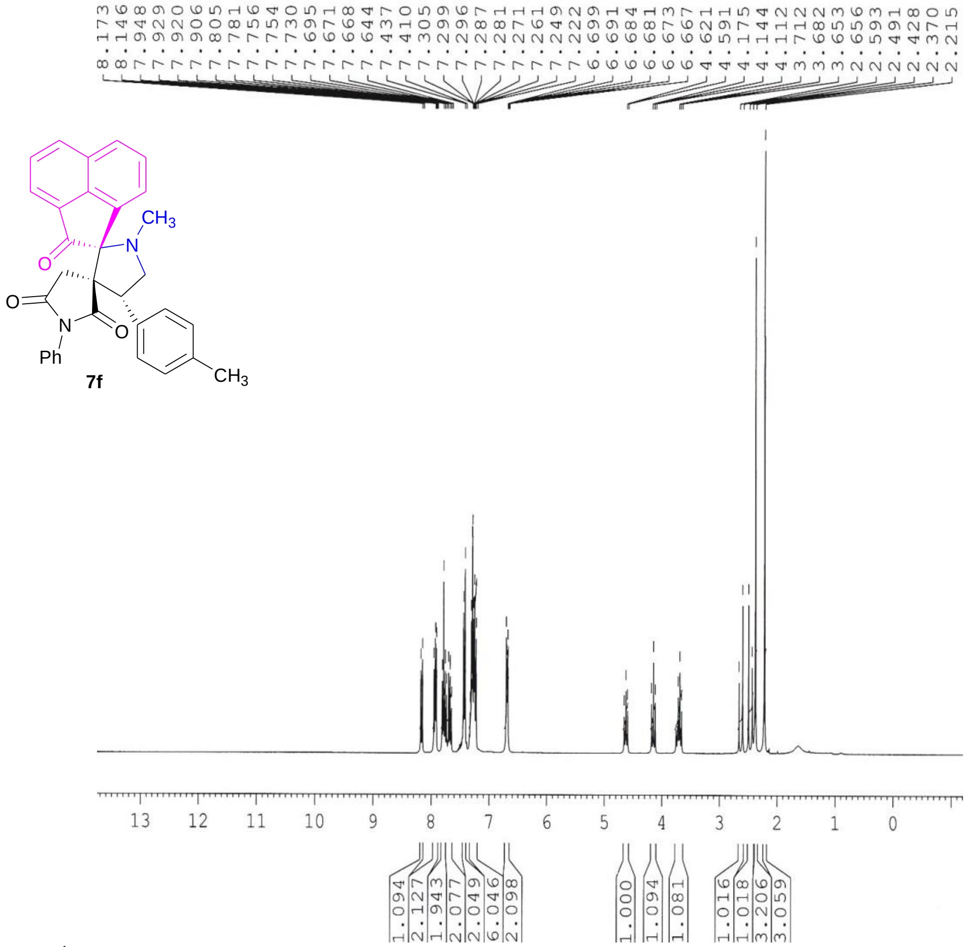

Fig. S59. ^{1}H NMR spectrum of **7f** in $CDCl_3$

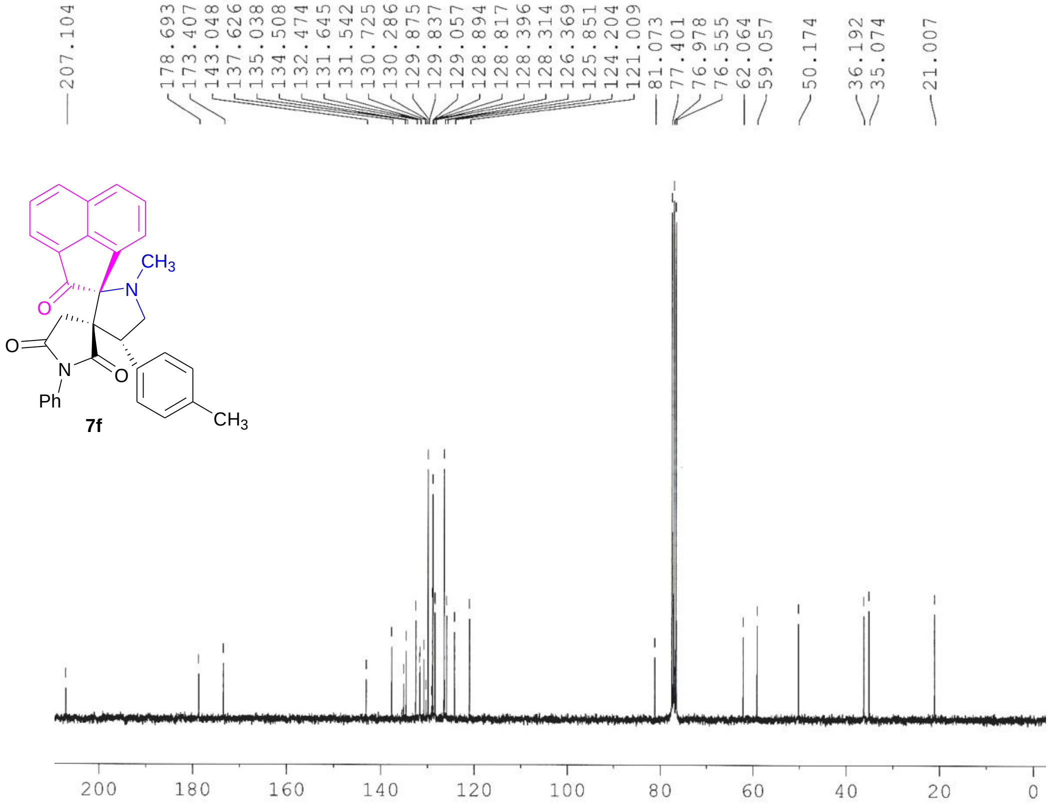

Fig. S60. ^{13}C NMR spectrum of **7f** in $CDCl_3$

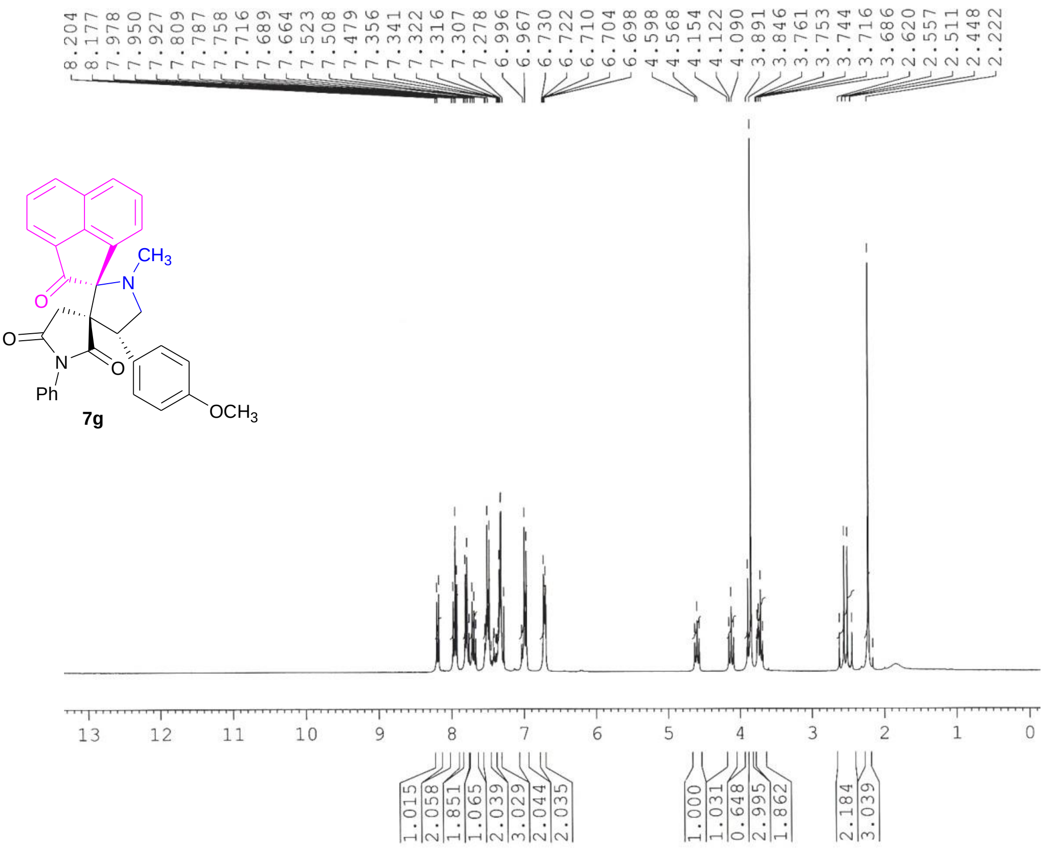

Fig. S61.^{1}H NMR spectrum of **7g** in $CDCl_3$

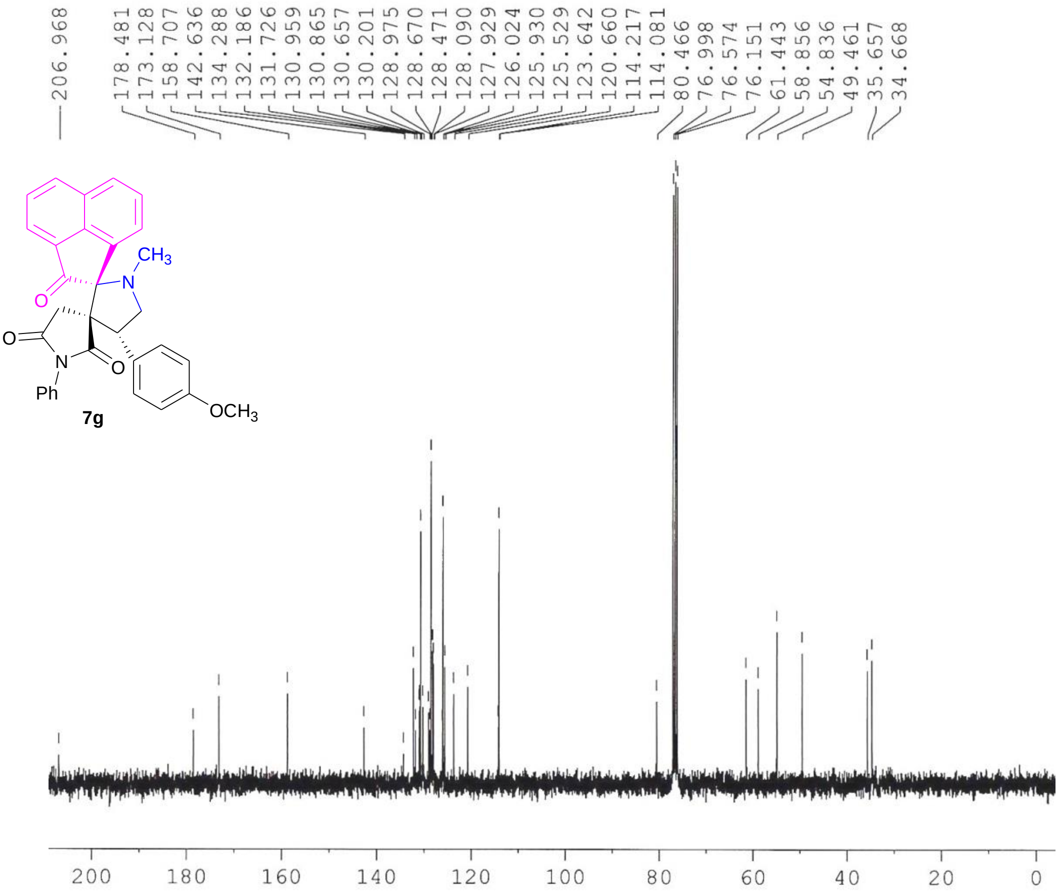

Fig. S62.^{13}C NMR spectrum of **7g**in $CDCl_3$

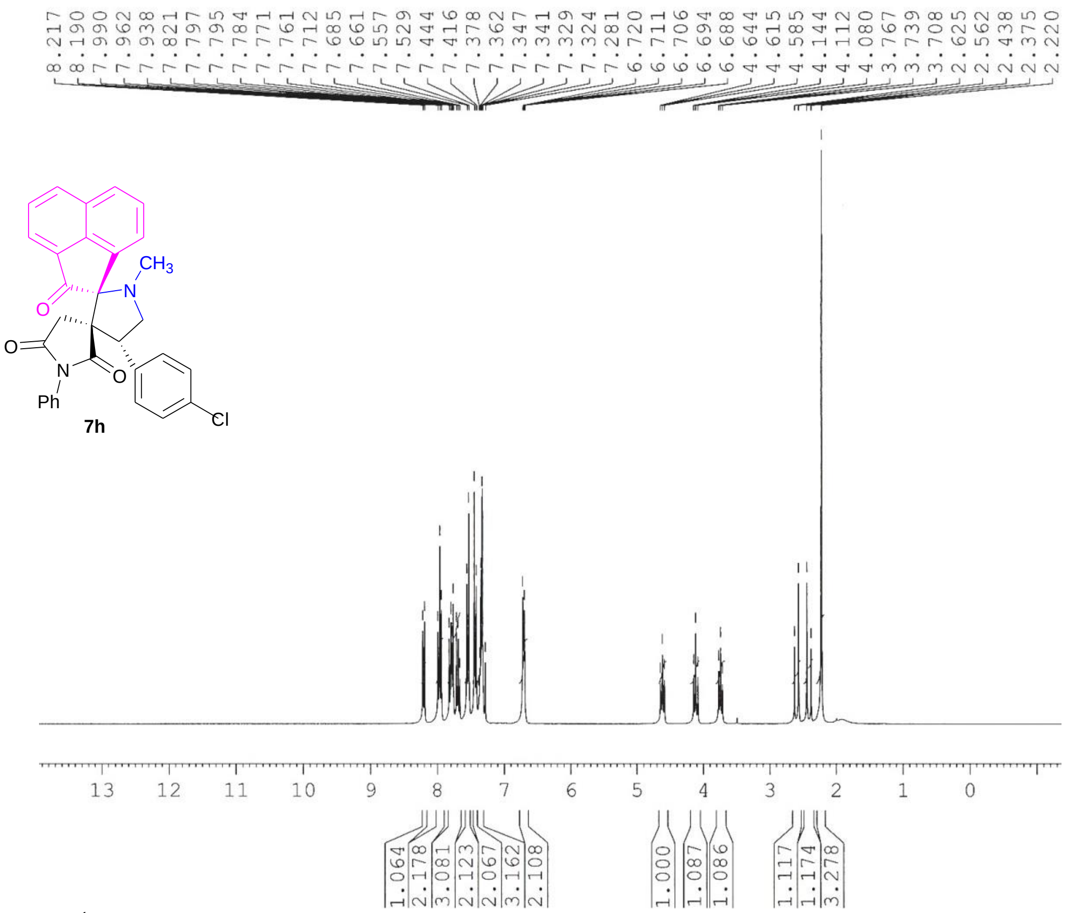

Fig. S63.^{1}H NMR spectrum of **7h** in $CDCl_3$

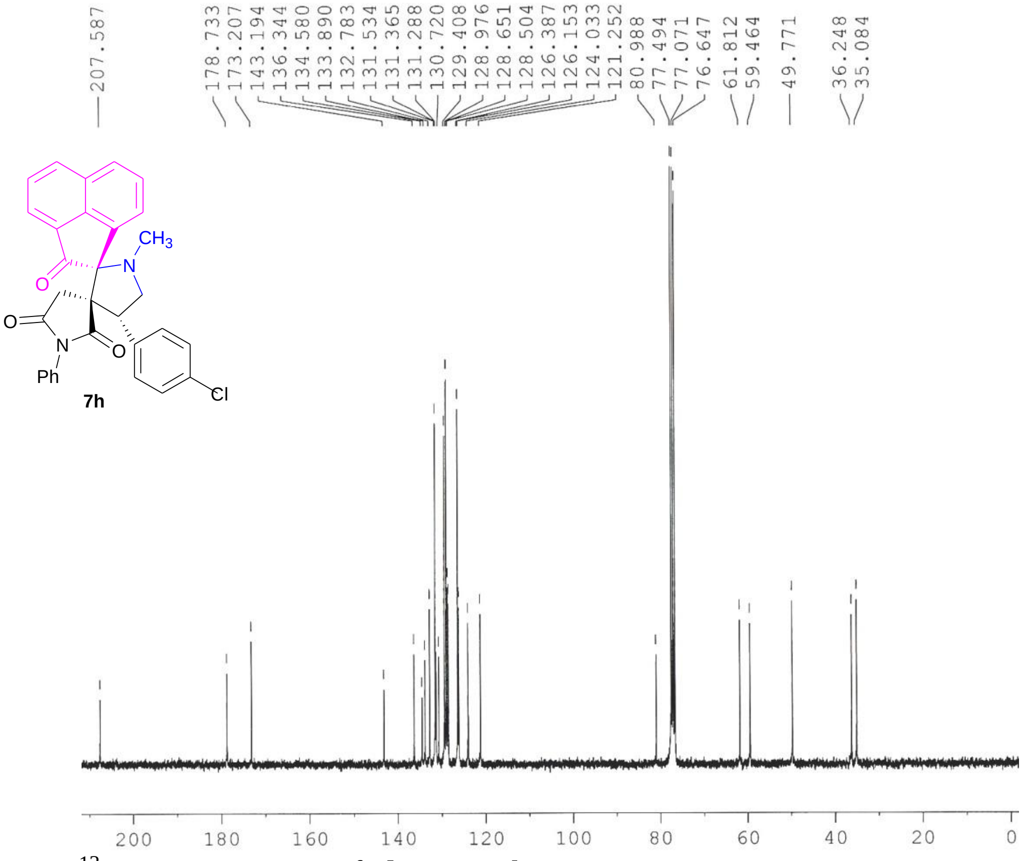

Fig. S64.^{13}C NMR spectrum of **7h** in $CDCl_3$

3. Cholinesterase inhibitory assay

Acetylthiocholine iodide (ATCl), acetylcholinesterase (AChE, from electric eel), butyrylcholinesterase (BChE, from equine serum), *S*-butyrylthiocholine chloride, and 5, 50-dithiobis(2-nitrobenzoicacid) (Ellman's reagent, DTNB) were purchased from Sigma–Aldrich (USA).

Cholinesterase enzyme inhibitory potential of the test samples was determined following method of Khaw et al. (2014) with slight modifications on the vehicle used. Briefly, test samples and galantamine were prepared in DMSO at the initial concentration of 0.5 mg/mL. The final concentration of DMSO in reaction mixture was 1%. At this concentration, DMSO has no inhibitory effect on both AChE and BChE enzymes. For AChE inhibitory assay, 140 µL of 0.1 M sodium phosphate buffer (pH 8) was added to a 96 wells microplate followed by 20 µL of test samples and 20 µL of 0.09 units/mL AChE enzyme. Then, 10 µL of 10 mM (DTNB was added into each well followed by 10 µL of 14 mM of acetylthiocholine iodide. The absorbance of the coloured end product was measured using Tecan Infinite 200 Pro Microplate Spectrophotometer at 412 nm for 30 min. Each test was conducted in triplicate. For BChE inhibitory assay, the same procedures were applied as AChE except for the use of enzyme and substrate, which were BChE from equine serum and S-butyrylthiocholine chloride. Absorbencies of the test samples were corrected by subtracting the absorbance of their respective blank (test samples in DMSO with substrate and DTNB, but without enzyme). A set of five concentrations was used to estimate the 50% inhibitory concentration (IC_{50}) for the compounds showing more than 50% inhibition at 5µg/mL concentration.

4. Molecular Docking of compound 4n

Molecular docking for **4m** was performed using Autodock 3.0.5 along with AutoDockTools (ADT) [1]. **4m** was built using Hyperchem 8 and energy minimization was performed with convergence criterion of 0.05 kcal/molA. Crystal structure of AChE from *Torpedo*

californica in complex with galanthamine was obtained from Protein Data Bank with PDB ID: 1W6R [2]. The protein was edited using ADT to remove all water molecules and all hydrogen atoms were added. Nonpolar hydrogen atoms and lone pairs were then merged and each atom was assigned with Gasteiger partial charges. A grid box of 41 × 53 × 41 points, with a spacing of 0.375A° was positioned at the center of active-site gorge. One hundred independent dockings were carried out for each docking experiment. The lowest docked energy of each conformation in the most populated cluster was selected. Analysis and visualization of the docking results was done using BIOVIA Discovery Studio visualizer.

[1] G. M. Morris, D. S. Goodsell, R. S. Halliday, R. Huey, W. E. Hart, R. K. Belew, A. J. Olson, *J. Comput. Chem.* 1998, 19, 1639–1662.

[2] H. M. Greenblatt, C. Guillou, D. Guenard, A. Argaman, S. Botti, B. Badet, C. Thal, I. Silman, J. L. Sussman, *J. Am. Chem. Soc.* 2004, 126, 15405–15411.
